# Digital Health Education and Training for Undergraduate and Graduate Nursing Students: Scoping Review

**DOI:** 10.2196/58170

**Published:** 2024-07-17

**Authors:** Manal Kleib, Antonia Arnaert, Lynn M Nagle, Shamsa Ali, Sobia Idrees, Daniel da Costa, Megan Kennedy, Elizabeth Mirekuwaa Darko

**Affiliations:** 1 Faculty of Nursing University of Alberta Edmonton, AB Canada; 2 Ingram School of Nursing McGill University Montreal, QC Canada; 3 Faculty of Nursing University of New Brunswick Fredericton, NB Canada; 4 Ingram School of Nursing McGill University Montreal, QC Canada; 5 Geoffrey & Robyn Sperber Health Sciences Library, University of Alberta Edmonton, AB Canada

**Keywords:** curriculum, digital health, health informatics, nursing education, nursing students, undergraduate, graduate

## Abstract

**Background:**

As technology will continue to play a pivotal role in modern-day health care and given the potential impact on the nursing profession, it is vitally important to examine the types and features of digital health education in nursing so that graduates are better equipped with the necessary knowledge and skills needed to provide safe and quality nursing care and to keep abreast of the rapidly evolving technological revolution.

**Objective:**

In this scoping review, we aimed to examine and report on available evidence about digital health education and training interventions for nursing students at the undergraduate and graduate levels.

**Methods:**

This scoping review was conducted using the Joanna Briggs Institute methodological framework and the PRISMA-ScR (Preferred Reporting Items for Systematic Reviews and Meta-Analyses extension for Scoping Reviews). A comprehensive search strategy was developed and applied to identified bibliographic databases including MEDLINE (Ovid; 1946 to present), Embase (Ovid; 1974 to present), CINAHL (EBSCOhost; 1936 to present), ERIC (EBSCOhost; 1966 to present), Education Research Complete (EBSCOhost; inception to present), and Scopus (1976 to present). The initial search was conducted on March 3, 2022, and updated searches were completed on January 11, 2023, and October 31, 2023. For gray literature sources, the websites of select professional organizations were searched to identify relevant digital health educational programs or courses available to support the health workforce development. Two reviewers screened and undertook the data extraction process. The review included studies focused on the digital health education of students at the undergraduate or graduate levels or both in a nursing program. Studies that discussed instructional strategies, delivery processes, pedagogical theory and frameworks, and evaluation strategies for digital health education; applied quantitative, qualitative, and mixed methods; and were descriptive or discussion papers, with the exception of review studies, were included. Opinion pieces, editorials, and conference proceedings were excluded.

**Results:**

A total of 100 records were included in this review. Of these, 94 records were identified from database searches, and 6 sources were identified from the gray literature. Despite improvements, there are significant gaps and limitations in the scope of digital health education at the undergraduate and graduate levels, consequently posing challenges for nursing students to develop competencies needed in modern-day nursing practice.

**Conclusions:**

There is an urgent need to expand the understanding of digital health in the context of nursing education and practice and to better articulate its scope in nursing curricula and enforce its application across professional nursing practice roles at all levels and career trajectories. Further research is also needed to examine the impact of digital health education on improving patient outcomes, the quality of nursing care, and professional nursing role advancement.

**International Registered Report Identifier (IRRID):**

RR2-10.11124/JBIES-22-00266

## Introduction

### Background

The World Health Organization (WHO) emphasized the important role of information and communication technologies (ICTs) in facilitating eHealth services and urged health systems to embrace emerging technologies such as artificial intelligence (AI) and big data analytics, considering their potential to radically change health outcomes. However, this requires intentional investments in people and processes as well as national-level strategies to realize the vision of a digitalized health sector [1]. Correspondingly, as the largest group of health care professionals, “there is an urgent need for the nursing workforce to acquire the skills and competencies to deliver high-quality, safe, optimized person-centred care in a digital health environment and to lead and participate in digital health initiatives, decision-making, and evaluation” [2].

*Digital health* is a new and evolving term that is often used interchangeably with other terms including *eHealth*, *mobile health*, *virtual care*, and *telehealth* to name a few [3]. These terms have evolved over time and can be understood by examining the eras of the industrial revolution impacting society, including health care. During the periods from 1950 to 1960 (Mainframe Computer Era) and 1970 to 2000 (Health IT Era), technological development was in its infancy, as such health care systems focused on the basic use of IT systems to manage enterprise information and logistics. The eHealth Era (2000-2020) witnessed an expansive use of ICTs such as electronic health records (EHRs) and increased consumer engagement in decision-making and self-care through digital technologies such as apps and personal health records [3]. In this period, terms such as *mHealth* and *eHealth* were popular. Services such as telehealth were also available but mostly as specialized and organization-based platforms. Telehealth refers to the “delivery and facilitation of health and health-related services including medical care, provider and patient education, health information services, and self-care via telecommunications and digital communication technologies. Examples of the technologies used in telehealth include but are not limited to live video conferencing, mobile health apps, ‘store and forward’ electronic transmission, and remote patient monitoring” [4]. The period between 2020 and beyond marked the Digital Health Era, which is anticipated to revamp health care as a result of the integration of more sophisticated technologies including AI, robotics, machine learning, the Internet of Things, virtual reality, and wearables. These advancements are shifting the focus of health care from the provider to a person-centered model and creating opportunities to improve health services modalities, system performance, therapeutics and treatments, and all aspects of health care [3]. During this period, the term *virtual care* emerged during the COVID-19 pandemic. Virtual health denotes the facilitation of the delivery of care services through any remote interactions between patients and health care providers and between health care providers themselves, whether synchronous or asynchronous, using ICTs [5].

Although some progress has been achieved in increasing nurses’ digital health capacity, the expanded and rapid integration of technological innovations in health care has created challenges for nursing educators and nursing programs to keep pace and ensure that nurses are well prepared to lead the digital transformation impacting professional practice roles and patient care [6-9]. In addition, while most nursing students have strong basic digital literacy skills, these skills do not necessarily translate into effective use of digital health technologies in the context of patient care [8,10,11]. Sometimes, assumptions about the use of technology in the academic setting put nursing students at a disadvantage, resulting in missed learning opportunities for students to develop competency in working with digital health technologies available in the clinical environment [8,12]. In Canadian nursing, approaches currently applied for preparing students at the undergraduate level in digital health are mainly focused on integrating informatics within existing courses; however, this integration is mostly inconsistent and sporadic [8,13,14]. Similar to the Canadian context, in other countries, the nursing informatics (NI) competencies, which should serve as a guiding framework for content integration in nursing curricula and as standards for professional practice requirements in the workplace, have limited to no focus on emerging technologies [15-17]. Furthermore, the adoption of these NI competencies in the workplace and their impact on patient outcomes remain largely unknown [8,18].

Nursing education is a key pathway for preparing nurses to assume professional roles in diverse practice settings. Providing nurses and nursing students with a comprehensive education in digital health should be an urgent priority, so they are better equipped with the necessary knowledge and skills needed to provide safe and quality nursing care and to keep abreast of the rapidly evolving technological revolution. This is also important so that nurses are better able to support patients and families as they navigate the health system and make decisions about using these technologies for health promotion and chronic disease management and to ensure that digital health services and technologies brought into the health care system are equitable, bias free, and accessible [2,9].

To identify current approaches for digital health education at the undergraduate and graduate nursing education levels, a preliminary search of available literature was conducted to identify prior work on this topic, and several reviews were retrieved. Some reviews focused on NI and digital health competency frameworks and the integration of NI into nursing curricula [15,16,18,19]. Other reviews addressed the learning outcomes of digital learning interventions in higher education [20] and technological literacy in nursing education [21]. The remaining reviews examined the influence of AI on different domains of nursing [22] and the effectiveness of telehealth educational interventions in graduate nursing education [23]. Another search was conducted on December 1, 2023, to identify if new reviews have been published since the initial search was conducted on January 5, 2022, and a scoping review protocol was found in CINAHL Plus database that focused on NI education in undergraduate nursing education [24].

On the basis of the evidence available on the digital health education for nursing students, we believe a gap exists in the literature, particularly assessing the current state with respect to how nursing education at the undergraduate and graduate nursing levels addresses digital health education about existing and emerging technologies. Therefore, this scoping review aimed to report on evidence available about digital health education and training interventions for nursing students at the undergraduate and graduate levels.

### Review Question

The review aimed to answer the following question: what are the types and features of digital health education and training interventions currently available to guide teaching and curricular integration or education about digital health for nursing students at the undergraduate and graduate levels? More specifically, this review analyzed and synthesized information on the following elements: (1) the definitions of digital health and learning objectives and topic content addressed in the digital health intervention; (2) the instructional strategies used and their delivery processes; (3) the pedagogical theories or frameworks used; and (4) the outcomes measured and evaluation or assessment strategies used for measuring them.

## Methods

The scoping review was conducted following the Joanna Briggs Institute methodology [25] and in line with the PRISMA-ScR (Preferred Reporting Items for Systematic Reviews and Meta-Analyses extension for Scoping Reviews) [26]. The review followed a priori protocol [27].

### Search Strategy

A health sciences librarian developed a comprehensive search strategy according to the PRISMA-S (Preferred Reporting Items for Systematic Reviews and Meta-Analyses for Searching) [28]. To ascertain the feasibility and testability of the search strategy, an initial search was conducted in CINAHL (EBSCOhost) as published in the study protocol [27]. All identified databases were searched, and the search strategy was adapted as appropriate. The following bibliographic databases were searched from inception to present: MEDLINE (Ovid; 1946 to present), Embase (Ovid; 1974 to present), CINAHL (EBSCOhost; 1936 to present), ERIC (EBSCOhost; 1966 to present), Education Research Complete (EBSCOhost; inception to present), and Scopus (1976 to present; Multimedia Appendix 1). The search used subject headings, wherever available, and appropriate keywords to capture relevant peer-reviewed literature. The search strategy was derived from two main concepts: (1) *digital health*, applying descriptors associated with the term such as virtual, telehealth, or remote delivery to capture the most relevant literature and (2) *nursing education*, both undergraduate and graduate level, as well as competencies and curricula. A multidatabase search was completed for ERIC and Education Research Complete, as these databases were available on the same platform, and the search strategy for these databases did not include any subject headings.

Only studies published from 2012 to 2023 were included because the authors wanted to capture current and relevant articles. Also included were studies published in the English language, as the authors speak only English. The non–peer-reviewed materials such as notes, editorials, letters, books, and book chapters were removed from the results, as they had limited information to contribute to the findings and discussion. The initial database searches were conducted on March 3, 2022, and updated searches were completed on January 11, 2023, and October 31, 2023. The same search strategy was used for each updated search to ensure consistency and identify any recently published papers. For gray literature sources, select relevant organizational sources were identified and searched for information regarding the digital health education programs that were being offered to gain insights on which topics were addressed and the target audiences for such education. Limiting this search to a few organizations was intentional, considering the volume of information that can be found on the web.

### Eligibility Criteria

#### Participants

This scoping review considered studies that included nursing students at the undergraduate or graduate levels admitted to public or private institutions. Furthermore, the review included students enrolled in undergraduate or graduate nursing programs, qualifying graduates for various nursing roles such as generalist entry-to-practice programs for registered nurses, licensed practical nurses or registered practical nurses, nurse practitioners (NPs), and registered psychiatric nurses. Empirical studies that reported on digital health education for qualified nurses working in practice settings and studies that reported on students in other health-related professions (eg, medicine, pharmacy, physiotherapy) or allied health staff (eg, health care aids) were excluded because these professions are not the subject of interest in this review.

#### Concept

The primary concepts of significance to the review are digital health education and training for nursing students. Other related concepts included are instructional strategies, delivery processes, pedagogical theory and frameworks, and evaluation strategies. Since the use of different technologies in health professionals’ education exists, the authors contend that the use of technologies for learning and teaching purposes differs from the use of digital health technologies for care delivery. Consequently, studies that focused on the use of instructional technologies, such as PowerPoint, simulation, and virtual reality for teaching or learning purposes in the classroom or laboratory or for learning about general clinical nursing skills, as opposed to education, learning, and training for acquisition of digital health knowledge and competence, were excluded. Studies that only examined an aspect of digital capabilities or NI competency, such as computer or information literacy, and focused on NI competency without explicitly linking the concept to digital health were excluded. Studies that focused on aspects of medical technologies, such as computed tomography scans and in vitro fertilization, were also excluded.

#### Context

This review considered studies that examined digital health education for nursing students admitted to educational institutions (colleges or universities) at undergraduate and graduate programs or both. There was no limit on the geographical location of the published studies, as the authors wanted to be comprehensive in their search and provide an in-depth analysis of the literature from multiple geographical locations.

#### Types of Sources

This scoping review considered all methodological and theoretical papers, including quantitative, qualitative, and mixed methods study designs. Systematic, scoping, integrative, umbrella, and narrative review studies were excluded but these types of studies were referred to in the *Discussion* section to provide further information. In addition, opinion pieces, editorials, and conference proceedings were also excluded due to the insufficient information these sources offer to contribute to the evidence available. Furthermore, a hand-search of reference lists of the included studies to search for missing studies was planned but not completed due to the large volume of searches retrieved. Selected gray literature sources were searched for additional evidence and insights.

### Study and Source of Evidence Selection

**Figure 1:**
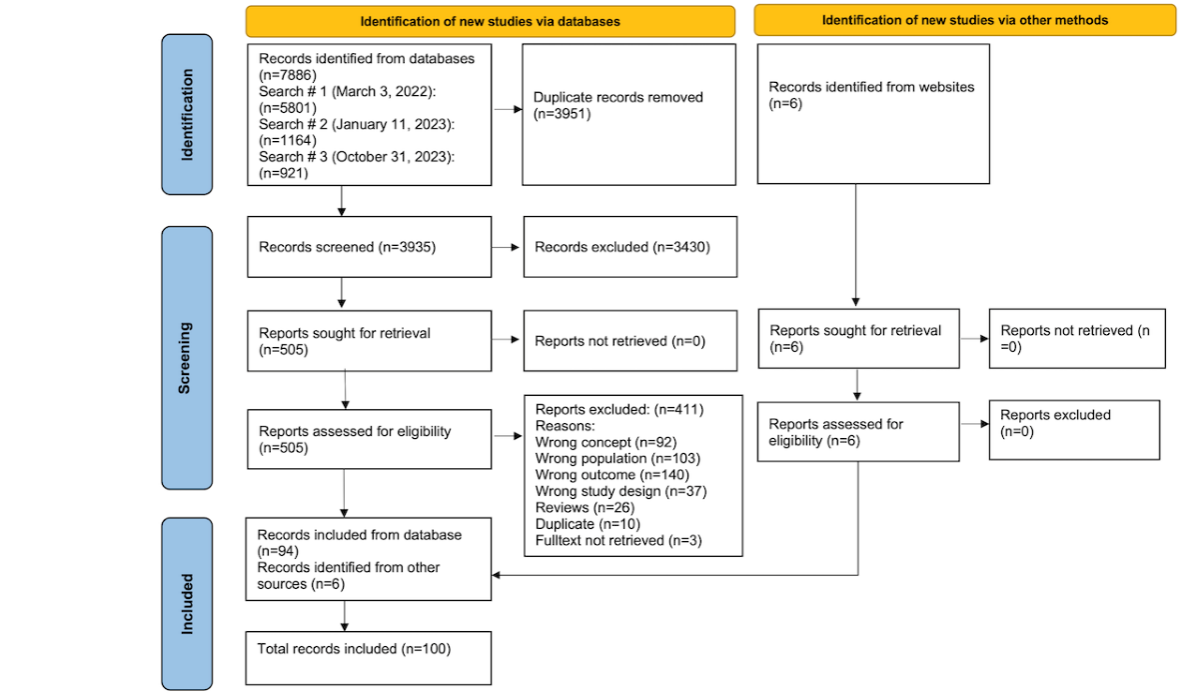
PRISMA (Preferred Reporting Items for Systematic Reviews and Meta-Analyses) chart.

Retrieved records were exported in complete batches into the Mendeley reference manager software (Mendeley Ltd) to generate bibliographies and the Covidence software (Veritas Health Innovation) for deduplication and to enable the screening process. To increase the reliability of the screening process, 2 reviewers (SI and SA) independently determined the eligibility of articles against the inclusion and exclusion criteria using a 2-stage screening process consisting of a title and abstract scan followed by a full-text review. All disagreements were resolved in consultation with other reviewers (M Kleib and EMD). The PRISMA (Preferred Reporting Items for Systematic Reviews and Meta-Analyses) chart was used to document inclusion and exclusion decisions and ensure transparency and rigor in reporting (Figure 1) [29,30]. Where full-text articles were excluded, reasons were provided for the exclusion (Multimedia Appendix 2).

### Data Extraction

Two independent reviewers (SI and SA) extracted data from the included studies and recorded it into an open-access Google spreadsheet. The included studies were extracted based on the extraction table as published in the a priori protocol [27]. To enhance reliability, the independent reviewers piloted the extraction table on 10 records (qualitative, quantitative, and mixed method study designs), and after comparing results, no further modifications were made to the extraction table. For each record, the following information was extracted: record information (ie, author or authors, year of publication, and study aim or purpose); population or sample (nursing students or level); context (country or program); concept (digital health existing and emerging technologies, the definition of digital health if provided, instructional strategy used and delivery process [eg, lecture, video, case-based scenario, pedagogical theory or framework, outcomes measured [eg, knowledge gain], assessment methods, or approaches [eg, instrument or tool, quizzes]); key findings; and recommendations. Any disagreements were resolved with other reviewers (M Kleib and EMD).

### Data Analysis and Interpretation

Basic descriptive statistics (ie, percentages or proportions) were applied to analyze and report key characteristics of studies included in the review. Using an iterative, descriptive approach, abstracted data from the included studies were examined for similarities and differences to identify patterns and facilitate thematic grouping of findings to answer the research questions. All members of the research team engaged in the discussion of the results and agreed upon the adequacy of the proposed thematic grouping. Where appropriate, a tabular format was used to provide a visual representation of the findings [25,31]. Appendices were used to provide access to information relevant to the conduct of this review and facilitate future research. Quality appraisal of the included studies was not completed, as it is not a requirement for scoping reviews.

## Results

### Study Inclusion

As shown in the PRISMA chart (Figure 1), combining all 3 searches together, we identified a total of 7886 studies. After removing duplicates and completing the first-level and second-level screening, 94 (1.19%) records were included from the database searches. Furthermore, 6 sources from the gray literature were included. These pertained to educational resources published on the websites of select organizations, including Digital Health Canada, Coursera, Healthcare Information and Management Systems Society, Canadian Nursing Informatics Association, Canadian Association of Schools of Nursing, and the Open WHO (Multimedia Appendix 3 [12,13,32-123]). In total, 100 records were included in this review.

### Characteristics of Included Studies

Of the 94 included studies from databases, the majority (n=67, 71%) were published within the last 5 years (2019-2023). Studies were mainly conducted in the United States (n=69, 73%) followed by Canada (n=8, 9%). The remaining were a few studies (n=17, 18%) from New Zealand, the United Kingdom, Australia, South Korea, Japan, Singapore, Norway, Democratic People's Republic of Korea, and Saudi Arabia. The research populations in these studies included representations from nursing students at the undergraduate and graduate levels and decision makers in charge of planning educational offerings. There were no studies involving licensed practical nurse students or their educational preparation in digital health.

### Review Findings

Table 1 provides a visual presentation of the range of studies included in this review classified according to the type and focus of the research study and the level of nursing education (undergraduate and graduate) in order to facilitate the reporting of the findings according to the review questions. Detailed abstraction tables of all the included studies from databases are available in Multimedia Appendix 4. The review of the 6 websites comprising the gray literature sources is also provided in Multimedia Appendix 3.

The included literature sources revealed a proliferation of educational offerings (Multimedia Appendix 4), and these sources were examined to identify whether or not authors have included a definition of digital health. A few studies involving undergraduate-level [13,104] and graduate-level education [83,84] cited the definition by Healthcare Information and Management Systems Society, which defined *digital health* as a health care delivery system that “connects and empowers people and populations to manage health and wellness through technology. Care is augmented by accessible and supportive provider teams working within flexible, integrated, interoperable, and digitally enabled care environments that strategically leverage digital tools, technologies, and services to transform care delivery” [124]. The definition of digital health proposed by the WHO, including the initial one in the draft of the global digital health strategy document published in 2019 or the one reported in final document, is as follows: “the field of knowledge and practice associated with the development and use of digital technologies to improve health. Digital health expands the concept of eHealth to include digital consumers, with a wider range of smart and connected devices. It also encompasses other uses of digital technologies for health such as the Internet of Things, advanced computing, big data analytics, artificial intelligence including machine learning, and robotics” [1].

**Table 1:** Overview of the records included in the review (n=100).

						Reference
**Database sources (n=94)**						
	**Interventional studies (n=61)**					
		**Undergraduate**				
			Telehealth (n=11)	[32-42]		
			EHR^a^ training (n=10)	[73-82]		
			NI^b^ (n=2)	[85,86]		
		**Graduate**				
			Telehealth (n=30)	[43-72]		
			EHR (n=2)	[87,88]		
			Digital health (n=2)	[83,84]		
			NI (n=4)	[89-92]		
	Curriculum status and integration (n=14)				[13,93-105]	
	Proposed strategies for integration (n=19)				[12,106-123]	
Gray literature sources (n=6)						—^c^

^a^EHR: electronic health record.

^b^NI: nursing informatics.

^c^Not applicable.

### Digital Health and NI Educational Interventions at the Undergraduate Level

#### Overview

At the undergraduate level, the main focus of the education delivered was on telehealth and telenursing [32-42] and competency development in using the EHR through simulated EHRs [73-82]. In total, 2 studies focused on NI education [85,86]. Of note, some of these interventions were implemented or developed in response to the COVID-19 pandemic [32-36,40].

#### Interventions Focused on Telehealth Education

The scope of the telehealth theoretical education in the included studies varied but mainly focused on the prepreparation for the telehealth simulation. In 1 study, a range of topics including telehealth etiquette, professionalism, peripherals, technologies, documentation, billing, collaboration, and history taking were taught [33], using different teaching modalities to deliver the content such as online modules [33], e-book, and video [32]. One study reported the use of a telehealth clinical placement experience [34], another study applied a web-based clinical experience [36], and the remaining studies applied different simulation activities [32,33,35,37-42].

Integration of simulation experiences was mostly as a stand-alone intervention; a few studies reported integration as part of an existing course. These studies integrated the simulation experiences as part of a rotation practice [38], course assignment [33], and as part of a clinical course [37,40]. Simulation was delivered mainly via teleconferencing and online technologies such as Zoom and Google Hangouts [32,33,35,36,40-42]. A few studies used a telehealth robot [33] or a telepresence robot [38,39]. The simulation experiences varied in length from 1 hour to a few hours, facilitated by using scenarios, standardized patients, briefing, and debriefing. Authors used different professional practice frameworks such as the American Association of Colleges of Nursing Essentials [33,40], National Organization of Nurse Practitioner Faculties [33], and Quality and Safety Education for Nurses [37] and best practices and theoretical frameworks for conducting simulation such as the International Nursing Association of Clinical Simulation and Learning and Promoting Excellence and Reflective Learning through Simulation method for debriefing [33,36,37].

A quantitative quasi-experimental design with a pretest-posttest [32,33,38] or posttest-only [39] and mixed methods research [34] approaches were mostly used to measure a variety of outcomes including knowledge, confidence, attitudes, communication, and overall experiences. Some studies also sought to determine the usability of the telehealth robot [33,39] or feasibility of the telehealth experience [35,36]. In addition to using pretest-posttest assessments, some authors used Objective Structured Clinical Examinations [32,35], knowledge tests [33,40], reflection [34,36,41], and focus group interviews [34,37,42]. Despite the inherent limitations of the study designs used, the interventions delivered yielded positive outcomes and students’ experiences and feedback. Two studies [32,38] reported a statistically significant change in the outcomes measured.

#### Interventions Focused on EHR Education

For studies involving EHR-related education, authors applied a variety of research designs mainly to pilot academic EHRs including mixed methods [74,75,80], case study [77], correlational design [81], surveys [73,76,78], think-aloud method [79], and focus groups [82]. Integration was mostly as a stand-alone intervention; a few studies reported integration as part of the first-year nursing clinical course [73], a clinical course [76], or as part of a fundamentals of nursing course [81]. A key aspect of using the simulated EHRs focused on developing documentation skills [73-75,79,82]. Case scenarios were used in most interventions; some included an additional didactic content [73] or provided orientation through videos, webinars, and opportunities to practice [74,75,81,82]. A few studies related the intervention to the required professional NI competencies or educational theories supporting simulation activities [75,80]. Outcomes of interest in these studies included knowledge, confidence, attitudes, satisfaction, experience, and perceived NI competency. Statistically significant findings were reported in some of these interventions [73,75,76]. Some studies also sought to evaluate the feasibility and suitability of the simulated record for use in a nursing program [74,75,78,79].

#### Interventions Focused on NI Education

In total, 2 studies addressed NI education [85,86]. Of these, 1 study applied a controlled interventional design to measure knowledge gain, attitudes toward the EHR, and perceived confidence following the completion of 2 learning modules on NI delivered via in-person lectures and online using vodcasts [86]. The other study used a 1-group pretest-posttest design following a 2-day online NI educational program and measuring perceived NI competency [85]. Both interventions were informed by professional practice standards or competencies and relevant educational theory, yielding a statistically significant improvements in the outcomes measured [85,86].

### Digital Health and NI Educational Interventions at the Graduate Level

#### Overview

Of the 38 studies identified in this category, 30 (79%) focused on telehealth interventions [43-72], 2 (5%) addressed digital health [83,84], 2 (5%) focused on EHRs [87,88], and 4 (11%) studies focused on NI education [89-92]. Of note, interventions focused on telehealth education were mostly delivered without situating this knowledge or skills within the broader digital health or NI context, despite some studies indicating the increased use of technology in the context of nursing practice. In addition, some of these studies were implemented or developed in response to the COVID-19 pandemic [43-45,47-49,51,53,55,57-59,71,83,84].

Most studies (32/38, 84%) enhanced the intervention design by incorporating theoretical and pedagogical frameworks such as Bandura’s self-efficacy theory [66,70]; Kolb’s Cycle of Experiential Learning [49,72]; Ericsson’s and Smith Expertise theory [68]; the Ottawa Model for Research [71]; the Plan-Do-Study-Act cycle [66]; the Technology Acceptance Model [60]; Roy’s Adaptation Model [59]; problem-based learning [62], Bloom’s Taxonomy [66,91]; Adult Learning Theories [89]; Nursing Education Healthcare Informatics Framework [92]; Technology Informatics Guiding Education Reform Competencies [92]; and professional standards and competencies, telehealth competencies, and best practices for simulation-based research [43-45,47,49,51-53,55-59, 61,63-65, 67,72,83,84,91].

#### Interventions Focused on Telehealth Education

Integration was mostly as a stand-alone intervention; however, a good number of studies reported integration as part of an existing theory or clinical course [44,45,47,48,50,51,53, 61-63,66,68,72]; theory courses included health policy, role transition course, advanced health assessment course, and bio-physical and integrated clinical diagnosis course. Of note, only a few studies engaged students in their final clinical practicum course in a telehealth clinical rotation experience [61]. Other students experienced telehealth during a clinical rotation [46] or as part of a clinical experience [69]. Telehealth education was delivered using a variety of educational modalities or strategies including didactic education (online modules and lectures, reading materials, videos, narrated lectures, and self-directed modules) with simulation scenarios and standardized patients [43-45,47,49,51,52,55,57, 58,62-64,67-70,72]; asynchronous and synchronous simulation using teleconferencing tools and interactivities with or without didactic education [51,52,58,59,63,65,71]; telehealth self-paced learning with discussion [48]; guest speaker lectures with self-paced modules, lectures, and videoconferencing demos [66,125]; simulation with a telehealth robot with an iPad and or a telehealth cart [54,55,64]; simulation with students acting as patients or providers [54,55,58,60]; telehealth as a clinical rotation [46,72]; telehealth Objective Structured Clinical Examinations and clinical examinations [47,63,65,67]; telehealth curriculum with supporting competencies [56]; telehealth focused on specific skills (eg, consultation, e-visit, triage) [50,54,55,60,64,65,68]; and partnership with clinical organizations [61,69].

The duration of telehealth simulation intervention varied from a few hours to days. Regarding topics covered in telehealth education, some studies reported on topics included, such as a broad overview of telehealth, technologies used in delivering telehealth, ways to engage with patients, telehealth competencies, laws and regulations related to telehealth practice, digital professionalism, and licensure requirements [56-58,64,66-72].

Researchers designed the interventional studies using different methods including mixed methods [43,55], pretest-posttest design [71], descriptive design [54], quasi-experimental design [44,50], formative and summative evaluation introduced as educational activities [45,48,51,53], program evaluations [46,47,49,56,63,65,69], pilot studies [43,52,55,57,59,60, 62,64,67,68], and quality improvement projects [58,66,71]. Some studies applied pretest-posttest assessments or pretest-posttest surveys to measure a variety of outcomes including knowledge, beliefs, confidence and comfort levels, interest in telehealth, attitudes, preparedness, and satisfaction with the learning [44-48,50,52,56,64,66,68,72], proficiency and competence in performing skills, clinical decision-making, working collaboratively with members of the health team, communication, providing care virtually, opinions, and experience of learning. In addition, some researchers evaluated the usability of telehealth technologies applied in simulation and simulation effectiveness [55]. Majority of these studies reported improvements after the intervention and positive student feedback; however, these measurements were either limited by the study design applied or the small sample size due to the heterogeneity of designs used (see Multimedia Appendix 4 for more details).

#### Interventions Focused on Digital Health

Only 2 studies [83,84] have actually used the term *digital health* and intentionally developed educational strategies to deliver such education to the students of the doctor of nursing practice program [83]. Of these studies, 1 study reported on developing an elective course on digital health that comprised 5 units [83], and the second study incorporated mind maps within a practicum experience to expose students to digital health technologies used in practice [84]. In this same study, the authors also reported that prior NI courses existed in the curriculum.

#### Interventions Focused on EHR Education

In total, 2 studies published by the same authors [87,88] reported on EHR-related learning by exposing students to simulated EHRs using an assignment strategy, with case scenarios integrated within health IT and NI courses, and assessing perceived NI competency. The initial evaluation [87] included an assessment of NI competence within the same group following the intervention; however, the second evaluation incorporated a control group [88].

#### Interventions Focused on NI Education

In total, 4 studies [89-92] reported on NI education; 1 (25%) study provided didactic education in the form of an online learning module [89]; 1 (25%) study developed a 4-week clinical practicum experience for using the electronic medical record [90]; and 2 (50%) studies reported on developing online courses in NI [91,92], but these were conducted in 2013 and 2014.

### Studies Reporting on Status of Curricular Integration

In total, 14 studies [13,93-105] focused on determining the status of digital health or NI integration in undergraduate and graduate nursing curricula using mainly survey designs [94,96,97,100,101,103,105]. Four studies focused on telehealth integration [93-96]. Of these, 2 studies included NP curricula [93,95], one applying an evaluation of a web-based telehealth (module-based) course [93] and the other reporting on program evaluation [95] following curriculum mapping, integration, and obtaining students’ feedback through comparing pretest-posttest surveys. The remaining 2 studies [94,96] used cross-sectional surveys of nursing programs involving both undergraduate- and graduate-level students, and both studies revealed variable levels of integration.

A total of 9 studies examined NI integration in nursing curricula using different methods including Delphi survey [97], internet search [98,100,103], case study [99], questionnaires [101], curriculum review [102], and mixed methods approach examining both NI and digital health [13,104], and 1 study examined the status of academic EHR use [105]. Variability was noted across these studies, but overall, some studies (4/9, 44%) that have implemented NI or digital health have noted improvement in students’ learning outcomes after integration [93,95,98,99].

### Proposed Strategies for Enhancing Digital Health and NI Education

A total of 14 studies [12,106-123] provided a discussion of strategies that could be used by nurse educators or nursing programs to strengthen undergraduate and graduate nursing education including AI competencies to inform undergraduate and graduate education [106], role of clinical preceptors in helping students learn about digital health [12], guidelines for health informatics [107], different telehealth educational strategies including how to incorporate or level telehealth competencies in the curriculum [108-112], strategies for NI education or curricular leveling [113-120], and EHR simulations [121,122]. Furthermore, 1 study focused on digital health and technology competency [123].

### Gray Literature Findings

A review of the websites of 6 organizations revealed an increase in the offering of educational programs and courses related to digital health, with some of them focused on emerging technologies. An overview of these findings is available in Multimedia Appendix 3.

## Discussion

### Principal Findings

The purpose of this review was to map the literature on digital health education, training courses, or other pedagogical interventions used for undergraduate and graduate nursing students and to inform the development of future educational interventions. Despite improvements, there are significant gaps and limitations in the scope of digital health education at the undergraduate and graduate levels, consequently posing challenges for nursing students to develop competencies needed in modern-day nursing practice.

In defining digital health, a few studies used the term *digital health* or provided educational strategies and content that capture the broad focus of digital health in nursing education. Several studies included in this review were conducted in the wake of the COVID-19 pandemic. The increased use of virtual care and telehealth practice mainly occurred at the graduate NP practice level, and both modalities are subsumed under digital health. Yet, the authors of these studies did not situate this education within the broader area of digital health or eHealth. Furthermore, the current educational approaches regarding digital health education are primarily focused on developing dimensions of NI competencies, that is, skills for using digital health technologies such as EHRs and telehealth.

According to the 2022 Nurse Practitioner Role Core Competencies in the United States, domain 8 Technology and Information Literacy includes 5 indicators focused on the application of ICTs [126]. These are also aligned to the American Association of Colleges of Nursing Essentials and Advanced-level Nursing Education, which emphasizes ICTs and informatics processes under domain 8 and provides 5 indicators, for example, indicator 8.4f: “employ electronic health, mobile, health, and telehealth systems to enable quality, ethical, and efficient patient care” may explain the focus on telehealth education at the NP education level within the included studies [126]. These guidelines also do not use the terminology of *digital health*. In Canada, the Canadian Nurse Practitioner Core Competency Framework, published in 2010, has an indicator 1.11: “Adheres to federal and provincial/territorial legislation, policies, and standards related to privacy, documentation, and information management (this also applies to verbal, written or electronic records)” [127], but no reference to digital health or NI was made.

Only two studies (2/38, 5%) focused on digital health as a concept to teach nursing students, with an increasing volume of studies on telehealth or telenursing education (30/38, 79%). Contrary to the findings of this review, the review by Foster and Adams [128] indicated inadequate research studies on telehealth education. The disparity in the findings could partly be due to the difference in the search periods, as our review included studies conducted before, during, and after the COVID-19 pandemic when the use of telehealth began to increase. Despite the difference in the findings, both reviews address the importance of timing and indicate the attention and relevance of telehealth education within nursing. Although most of the educational interventions about digital health at the graduate level addressed telehealth, the integration and education about telehealth are still inadequate as identified in this review and prior reviews [129]. In addition, a recent national survey conducted by Eckoff et al [94], which examined the telehealth education in both prelicensure and graduate nursing education, also revealed inconsistency and limited education about telehealth.

No studies indicated teaching NI or digital health as a unique course within the nursing curriculum. In addition, despite the increased attention to the potential impact of AI on nursing education and practice, no interventional studies that addressed AI education at the undergraduate or graduate levels were found. Only 1 study [106] identified AI competencies to guide nursing education in Canada despite urgent calls for providing formal training and education of health care providers and nurses at the basic and advanced levels in AI. The proliferation of AI technologies in nursing education and clinical practice shows the need for proactive measures to integrate AI education and its related competencies in nursing education [22,130].

With respect to the scope of educational strategies currently being used, these included didactic approaches such as training sessions or webinars, online modules, prerecorded lectures, PowerPoint presentations, and video clips; experiential approach that mostly applies simulation encounters along with debriefing, guided exercises, and opportunities for question or answer; or a combination of both. To enhance students’ experiential learning, the educational strategies were delivered through online and face-to-face means [131]. The choice of these strategies was largely based on the objectives of the study and the interventional design applied. More advanced educational strategies, such as virtual and augmented reality, are limited in teaching nursing students about digital health and NI. As these technologies become more mainstream, it is anticipated that future research will shed light on the value and effectiveness of these strategies in nursing education, specifically in the digital health education [8,9]. It is promising to see that educators and scholars have shared their expertise and the strategies that they have applied in their programs for integrating digital health and NI; this could serve to encourage educators to consider applying these strategies within their programs or day-to-day teaching in order to improve graduate outcomes and increase their capacity for optimal practice in digital health care environments.

It is noted that the theoretical education about digital health and NI as core concepts in the nursing curricula remains variable and is mostly focused on the skills component as opposed to providing comprehensive and foundational knowledge that would help students understand the full picture of the digital health revolution. This was also corroborated by the findings from studies that examined the status of NI and digital health integration in nursing curricula, demonstrating variable levels of integration at both the undergraduate and graduate levels. These findings suggest that digital health and NI are not yet a priority in nursing education; however, studies reviewed were mostly survey studies examining the state of education at a point in time.

Several papers in the included studies have incorporated theoretical, conceptual, pedagogical, and professional standards in designing their interventions. Using such frameworks is highly recommended because it provides an evidence-based approach for planning, implementation, and evaluation of the educational intervention [132]. It also enables researchers to expand on the body of knowledge available to inform nursing education and practice based on best practices for knowledge generation. Incorporating theory in the intervention design also increases the intervention fidelity; however, the effectiveness of these interventions can be limited by a small sample size and the lack of experimental control in measuring the outcomes of interest.

The evaluative strategies used in these studies depended on the study design and the expected outcomes. Although not all the included studies assessed an intervention, some studies used a single, multiple, or a combination of assessment strategies or tools to undertake summative or formative assessment to determine the effectiveness of the intervention. Therefore, the assessment served as the measurement of the intervention done or the process instituted. Similar to the findings of Hui et al [131], authors of studies included in this scoping review also identified multiple and written assessment tasks as evaluative strategies used in assessing telehealth education implemented in a health curriculum. Of note is that some studies (35/61, 57%) that used theoretical frameworks for the intervention design also developed assessment strategies or used existing validated instruments to evaluate or measure outcomes.

Concerning the outcome measures examined, the outcome measure for undergraduate and graduate levels had some similarities and differences. Regarding the similarities, students at the undergraduate and graduate levels were assessed on their levels of competencies related to an educational intervention. However, at the graduate level, in addition to the competencies identified at the undergraduate level, the expectations and outcomes measured were more complex and advanced. After completing graduate-level education, graduate students are expected to assume leadership with the delivery and implementation of telehealth; hence, the education and training at the graduate level on telehealth is more comprehensive, and the educational interventions were often designed in alignment with advanced practice standards and competencies as well as frameworks such as the telehealth competence framework [133].

With respect to the results from the gray literature, it is interesting to note that different organizations in the United States and Canada provided a wide range of courses in different areas of practice related to digital health (Multimedia Appendix 3). This may reflect an increased interest in digital health or a demand among health care professionals or their employers for such information. This is encouraging and can be used as a guidepost for nursing educational programs with respect to the importance and scope of content that can be incorporated into formal nursing education at the undergraduate and graduate levels. While these educational offerings primarily target health care professionals in practice, they can also be used by students particularly at the graduate level. It can also serve as a resource for nurse educators or practitioners to pursue continuing education or increase their knowledge in areas that are evolving rapidly in health care, such as AI; for example, the WHO course named *Ethics and Governance of AI for Health* is one example.

The limitations in the scope of the digital health education both at the undergraduate and graduate levels could be attributed, in part, to the fact that digital health as a field is still evolving. The definition of digital health may not necessarily be known or used by nursing scholars, and the term is also new and will likely further evolve as technology advances in the years to come. Although this may pose challenges for nurse educators and programs to clearly articulate the scope of digital health education in nursing curricula, standardization in either the definition or the dimensions of digital health education should not be perceived as a barrier for nursing programs and educators to begin teaching their students about digital health.

Nursing education programs and educators are encouraged to integrate what is known about digital health as it applies to nursing in their curricula and to keep abreast of the developments in this field so that nurses are not left behind. In addition, upgrading existing NI competency standards, particularly, from the entry level to practice level, in order to account for developments in the field of digital health and providing resources for nurse educators on how to operationalize these indicators in their day-to-day teaching are needed to expedite this process of integration. As the field will continue to evolve, periodic revisions of the NI competency standards should also be considered.

### Implications

The dynamic nature of the health care system continuously evolving as a result of technological advancement demands that nursing students have opportunities to develop a baseline knowledge and competency in digital health and to cultivate this knowledge through continuing education upon becoming independent practitioners. From an equity perspective, all nursing students should have the opportunity to receive comprehensive digital health education because they represent the future health care workforce that is already faced with significant challenges to overcome including aging population, technological disruption, globalization, population displacement, and climate change to name a few [2]. As such, digital health education should not be a side topic in the nursing curriculum or be taught on a need-to-know basis, but rather it should be comprehensively embedded throughout all levels of nursing education and nursing career trajectories.

This scoping review provided important insights into the current state of digital health education and the modalities available for teaching nursing students. In light of the gaps and limitations identified in this review, enhancing the digital health education for nurses and nursing students should be a policy priority. A comprehensive education about digital health should provide foundational knowledge in core concepts relative to the existing and new digital health care technologies and create opportunities for learners to continuously reflect on their practice as well as be able to identify areas for growth and development as the digital health ecosystem evolves. Upgrading nursing education by introducing new strategies, such as virtual and augmented reality and AI generative platforms, to deliver and augment learning allows nursing students to think critically about these technologies and, by extension, other similar applications that will eventually make their way into clinical practice [124,134]. Follow-up studies may also be beneficial to determine the impact of digital health and informatics education in the workplace.

### Conclusions

As the digital health ecosystem continues to evolve, nursing education and practice must evolve too. There is an urgent need to expand the understanding of digital health in the context of nursing education and practice and to better articulate its scope in nursing curricula and enforce its application across professional nursing practice roles at all levels and career trajectories. Further research is also needed to examine the impact of digital health education on improving patient outcomes, the quality of nursing care, and professional nursing role advancement.

## Appendix

Multimedia Appendix 1 Search strategy.

Multimedia Appendix 2 List of excluded studies.

Multimedia Appendix 3 Abstraction tables.

Multimedia Appendix 4 Organizations providing digital health education.

Multimedia Appendix 5 PRISMA-ScR (Preferred Reporting Items for Systematic Reviews and Meta-Analyses extension for Scoping Reviews) checklist.

## Abbreviations

AI: artificial intelligence
EHR: electronic health record
ICT: information and communication technology
NI: nursing informatics
NP: nurse practitioner
PRISMA: Preferred Reporting Items for Systematic Reviews and Meta-Analyses
PRISMA-S: Preferred Reporting Items for Systematic Reviews and Meta-Analyses for Searching
PRISMA-ScR: Preferred Reporting Items for Systematic Reviews and Meta-Analyses extension for Scoping Reviews
WHO: World Health Organization
