# Supplementary material for: Digital Health Education and Training for Undergraduate and Graduate Nursing Students: Scoping Review
Source: JMIR Nurs. 2024 Jul 17;7:e58170. doi: 10.2196/58170 (PMC11292154; doi:10.2196/58170)
Supplement: Multimedia Appendix 1 [file nursing_v7i1e58170_app1.docx]

**Appendix I: Search strategy**

| **Database** | **Platform** | **Date of Search** | **Number of Results** |
| --- | --- | --- | --- |
| CINAHL Plus with Full Text | EBSCOhost | March 3, 2022  January 11, 2023  October 31, 2023 | 1245  254  187 |
| ERIC | EBSCOhost | March 3, 2022  January 11, 2023  October 31, 2023 | 22  3  2 |
| Education Research Complete | EBSCOhost | March 3, 2022  January 11, 2023  October 31, 2023 | 426  100  72 |
| Medline | OVID | March 3, 2022  January 11, 2023  October 31, 2023 | 1144  206  161 |
| EMBASE | OVID | March 3, 2022  January 11, 2023  October 31, 2023 | 1252  235  188 |
| Scopus | Elsevier | March 3, 2022  January 11, 2023  October 31, 2023 | 1712  366  311 |
|  | Total results before deduplication using Covidence | | 7886 |

**Database: CINAHL Plus with Full Text (1936 - Present) via EBSCOhost**

**Date of search: October 31, 2023**

| **S/N** | **Search Words** | **Results** |
| --- | --- | --- |
| S1 | (MH "Telehealth") OR (MH "Telenursing") OR (MH "Telemedicine") OR (MH "Remote Consultation") | 29,451 |
| S2 | (MH "Nursing Informatics") OR (MH "Health Informatics") OR (MH "Medical Informatics") | 12,614 |
| S3 | (digital N3 (health or care or healthcare or consult* or visit* or medicine or nursing)) | 3,764 |
| S4 | (virtual N3 (health or care or healthcare or consult* or visit* or medicine or nursing)) | 5,571 |
| S5 | (("remote* deliver*" or "online deliver*") N3 (health or care or healthcare or consult* or visit* or medicine or nursing)) | 45 |
| S6 | telehealth or tele-health or telemedicine or tele-medicine or telenursing or tele-nursing | 31,767 |
| S7 | eHealth or e-health or mhealth or m-health or (mobile N2 (health or care or healthcare or consult* or medicine or nursing or app# or application*)) | 36,395 |
| S8 | (nursing or medicine or medical or health) N2 informatics | 19,682 |
| S9 | "health* information technolog*" | 3,346 |
| S10 | "technolog* driven health*" | 10 |
| S11 | S1 OR S2 OR S3 OR S4 OR S5 OR S6 OR S7 OR S8 OR S9 OR S10 | 76,771 |
| S12 | (MH "Education, Nursing") OR (MH "Education, Nursing, Baccalaureate+") OR (MH "Education, Nursing, Graduate+") OR (MH "Education, Nursing, Diploma Programs") | 69,051 |
| S13 | ((MH "Curriculum+") OR (MH "Education")) AND AB (nurs*) | 9,663 |
| S14 | TI (nurs* N3 (educat* or curricul* or learn* or teach* or instruct*)) OR AB (nurs* N3 (educat* or curricul* or learn* or teach* or instruct*) | 65,123 |
| S15 | (MH "Professional Competence") | 19,157 |
| S16 | TI ( (competen* or skill* or knowledge) N3 ( "nurs* student*" or "nurs* educat*")) OR AB ( (competen* or skill* or knowledge) N3 ( "nurs* student*" or "nurs* educat*")) | 3,785 |
| S17 | S12 OR S13 OR S14 OR S15 OR S16 | 129,697 |
| S18 | S11 AND S17 "Limiters - Scholarly (Peer Reviewed) Journals; English Language; Published Date: 20120101-20231031 | 1671 |

**Database: ERIC and Education Research Complete (multi-database search; inception to Present) via EBSCOhost**

**Date of search: October 31, 2023**

| **S/N** | **Search Words** | **Results** |
| --- | --- | --- |
| S1 | (telehealth* or tele-health* or telemedicine or tele-medicine or telenursing or tele-nursing or teleconsultation* or tele-consultation*) | 2,177 |
| S2 | ((nursing or medicine or medical or health*) N2 informatics) | 4,200 |
| S3 | (digital* N3 (health or care or healthcare or consult* or visit* or medicine or nursing)) | 884 |
| S4 | (virtual* N3 (health or care or healthcare or consult* or visit* or medicine or nursing)) | 686 |
| S5 | ((internet* or web*) N3 (health or care or healthcare or consult* or visit* or medicine or nursing)) | 127,044 |
| S6 | (("remote* deliver*" or "online deliver*") N3 (health or care or healthcare or consult* or visit* or medicine or nursing)) or "video consult*" | 109 |
| S7 | (eHealth or e-health or mhealth or m-health or (mobile N2 (health or care or healthcare or consult* or medicine or nursing or app# or application*))) | 10,429 |
| S8 | "health* information technolog*" | 373 |
| S9 | "technolog* driven health*" | 1 |
| S10 | S1 OR S2 OR S3 OR S4 OR S5 OR S6 OR S7 OR S8 OR S9 | 140,682 |
| S11 | TI ( (nurs* N3 (educat* or curricul* or learn* or teach* or instruct* or student* or program* or course*)) ) OR AB ( (nurs* N3 (educat* or curricul* or learn* or teach* or instruct* or student* or program* or course*)) ) | 27,022 |
| S12 | TI ( ((competen* or skill* or knowledge) N3 ("nurs* student*" or "nurs* educat*" or "nurs* school*" or "nurs* graduat*")) ) OR AB ( ((competen* or skill* or knowledge) N3 ("nurs* student*" or "nurs* educat*" or "nurs* school*" or "nurs* graduat*")) ) | 1,482 |
| S13 | competen* AND nurs* | 8,233 |
| S14 | S11 OR S12 OR S13 | 30,894 |
| S15 | S10 AND S14 "Limiters - Peer Reviewed; Published Date: 20120101-20231031  Narrow by Language: - english | 664 |

Results from ERIC = 30

Results from Education Research Complete = 634

**Database: Ovid MEDLINE(R) ALL <1946 to October 30, 2023>**

**Date of search: October 31, 2023**

| **S/N** | **Search Words** | **Results** |
| --- | --- | --- |
| 1 | Telemedicine/ | 38070 |
| 2 | Remote Consultation/ | 5771 |
| 3 | (telehealth* or tele-health* or telemedicine or tele-medicine or telenursing or tele-nursing).mp. | 55975 |
| 4 | medical informatics/ or nursing informatics/ or public health informatics/ | 15723 |
| 5 | ((nursing or medicine or medical or health*) adj2 informatics).mp. | 24294 |
| 6 | (digital* adj3 (health or care or healthcare or consult* or visit* or medicine or nursing)).mp. | 12651 |
| 7 | (virtual* adj3 (health or care or healthcare or consult* or visit* or medicine or nursing)).mp. | 5848 |
| 8 | ((internet* or web*) adj3 (health or care or healthcare or consult* or visit* or medicine or nursing)).mp. | 12412 |
| 9 | (("remote* deliver*" or "online deliver*") adj3 (health or care or healthcare or consult* or visit* or medicine or nursing)).mp. | 129 |
| 10 | (eHealth or e-health or mhealth or m-health or (mobile adj2 (health or care or healthcare or consult* or medicine or nursing or app# or application*))).mp. | 42322 |
| 11 | "health* information technolog*".mp. | 4511 |
| 12 | "technolog* driven health*".mp. | 14 |
| 13 | or/1-12 | 134634 |
| 14 | education, nursing/ or education, nursing, associate/ or education, nursing, baccalaureate/ or education, nursing, diploma programs/ or education, nursing, graduate/ | 65593 |
| 15 | (education/ or exp curriculum/ or education, professional/) and nurs*.ab. | 13230 |
| 16 | (nurs* adj3 (educat* or curricul* or learn* or teach* or instruct* or student*)).ti,ab. | 66229 |
| 17 | Professional Competence/ | 25135 |
| 18 | ((competen* or skill* or knowledge) adj3 ("nurs* student*" or "nurs* educat*")).ti,ab. | 2702 |
| 19 | 14 or 15 or 16 or 17 or 18 | 128641 |
| 20 | 13 and 19 | 2504 |
| 21 | limit 20 to (english language and yr="2012 -Current") | 1471 |
| 22 | limit 21 to (comment or editorial or letter) | 28 |
| 23 | 21 not 22 | 1443 |

**Database: OVID Embase <1974 to 2023 October 30>**

**Date of search: October 31, 2023**

| **S/N** | **Search Words** | **Results** |
| --- | --- | --- |
| 1 | telemedicine/ or telehealth/ or exp teleconsultation/ or video consultation/ | 75215 |
| 2 | (telehealth* or tele-health* or telemedicine or tele-medicine or telenursing or tele-nursing or teleconsultation* or tele-consultation*).mp. | 85269 |
| 3 | medical informatics/ | 23369 |
| 4 | nursing informatics/ | 1725 |
| 5 | ((nursing or medicine or medical or health*) adj2 informatics).mp. | 30433 |
| 6 | (digital* adj3 (health or care or healthcare or consult* or visit* or medicine or nursing)).mp. | 13837 |
| 7 | (virtual* adj3 (health or care or healthcare or consult* or visit* or medicine or nursing)).mp. | 8079 |
| 8 | ((internet* or web*) adj3 (health or care or healthcare or consult* or visit* or medicine or nursing)).mp. | 15214 |
| 9 | (("remote* deliver*" or "online deliver*") adj3 (health or care or healthcare or consult* or visit* or medicine or nursing)).mp. | 172 |
| 10 | mobile health application/ | 4339 |
| 11 | (eHealth or e-health or mhealth or m-health or (mobile adj2 (health or care or healthcare or consult* or medicine or nursing or app# or application*))).mp. | 50594 |
| 12 | health* information technolog*.mp. | 4790 |
| 13 | technolog* driven health*.mp. | 17 |
| 14 | or/1-13 | 177350 |
| 15 | nursing education/ | 89416 |
| 16 | (education/ or course content/ or curriculum/ or curriculum development/ or education program/) and nurs*.ab. | 59809 |
| 17 | (nurs* adj3 (educat* or curricul* or learn* or teach* or instruct* or student*)).ti,ab. | 70657 |
| 18 | (competence/ or professional competence/) and nurs*.ab. | 5588 |
| 19 | nursing competence/ | 1242 |
| 20 | ((competen* or skill* or knowledge) adj3 ("nurs* student*" or "nurs* educat*")).ti,ab. | 2715 |
| 21 | or/15-20 | 159601 |
| 22 | 14 and 21 | 3557 |
| 23 | limit 22 to (english language and yr="2012 -Current") | 2401 |
| 24 | limit 23 to (books or chapter or conference abstract or "conference review" or editorial or letter or note) | 786 |
| 25 | 23 not 24 | 1615 |

**Database: Scopus (1976 - Present)**

**Date of search: October 31, 2023**

| **Search Words** | **Results** |
| --- | --- |
| ( ( ( TITLE-ABS-KEY ( ( telehealth* OR tele-health* OR telemedicine OR tele-medicine OR telenursing OR tele-nursing OR teleconsultation* OR tele-consultation* ) ) OR TITLE-ABS-KEY ( ( ( nursing OR medicine OR medical OR health* ) W/2 informatics ) ) OR TITLE-ABS-KEY ( ( digital* W/3 ( health OR care OR healthcare OR consult* OR visit* OR medicine OR nursing ) ) ) OR TITLE-ABS-KEY ( ( virtual* W/3 ( health OR care OR healthcare OR consult* OR visit* OR medicine OR nursing ) ) ) OR TITLE-ABS-KEY ( ( ( internet* OR web* ) W/3 ( health OR care OR healthcare OR consult* OR visit* OR medicine OR nursing ) ) ) OR TITLE-ABS-KEY ( ( ( "remote* deliver*" OR "online deliver*" ) W/3 ( health OR care OR healthcare OR consult* OR visit* OR medicine OR nursing ) ) OR "video consult*" ) OR TITLE-ABS-KEY ( ( ehealth OR e-health OR mhealth OR m-health OR ( mobile W/2 ( health OR care OR healthcare OR consult* OR medicine OR nursing OR app# OR application* ) ) ) ) ) ) OR ( ( TITLE-ABS-KEY ( "health* information technolog*" ) OR TITLE-ABS-KEY ( "technolog* driven health*" ) ) ) ) AND ( ( TITLE-ABS-KEY ( ( nurs* W/3 ( educat* OR curricul* OR learn* OR teach* OR instruct* OR student* ) ) ) OR TITLE-ABS-KEY ( ( ( competen* OR skill* OR knowledge ) W/3 ( "nurs* student*" OR "nurs* educat*" OR "nurs* school*" OR "nurs* graduat*" ) ) ) OR TITLE-ABS-KEY ( competen* AND nurs* ) ) ) AND PUBYEAR > 2011 AND PUBYEAR < 2024 AND ( LIMIT-TO ( DOCTYPE , "ar" ) OR LIMIT-TO ( DOCTYPE , "re" ) ) AND ( LIMIT-TO ( LANGUAGE , "English" ) ) | 2332 |
