# Supplementary material for: Digital Health Education and Training for Undergraduate and Graduate Nursing Students: Scoping Review
Source: JMIR Nurs. 2024 Jul 17;7:e58170. doi: 10.2196/58170 (PMC11292154; doi:10.2196/58170)
Supplement: Multimedia Appendix 2 [file nursing_v7i1e58170_app2.docx]

**Appendix II - List of Excluded Studies - AMA 11^th^ Edition Citation Style (n=411)**

| **Reason for Exclusion** | **Number of Records** |
| --- | --- |
| Wrong concept | 92 |
| Wrong population | 103 |
| Wrong outcome | 140 |
| Wrong study design | 37 |
| Reviews | 26 |
| Duplicate | 10 |
| Full-text not retrieved. | 3 |

1. Abdrbo AA. Nursing informatics competencies among nursing students and their relationship to patient safety competencies. CIN: Computers, Informatics, Nursing. 2015;33(11):509–14. doi:10.1097/CIN.0000000000000197

Reason: Wrong outcome

1. Abdekhoda M, Khezri H. Assessing nurses’ informatics competency and identifying its related factors. J Res Nurs. 2019;24(7):529-538. doi:10.1177/1744987119839453

Reason: Wrong population

1. Abdekhoda M, Khezri H. Investigating the impact of health informatics literacy on the evidence-based practice of nursing. Online J Nurs Informatics. 2021;25(2):4-4.

Reason: Wrong population

1. Abu Raddaha AH. Nurses’ perceptions about and confidence in using an electronic medical record system. Proc Singapore Healthc. 2018;27(2):110-117. doi: 10.1177/2010105817732585

Reason: Wrong population

1. Achampong EK. Assessing the current curriculum of the nursing and midwifery informatics course at all nursing and midwifery institutions in Ghana. J Med Educ Curric Dev. 2017;4:2382120517706890. doi:10.1177/2382120517706890

Reason: Wrong outcome

1. Afra A, Elahi N, Langarizadeh M, Beiramipour A. Need assessment for nursing informatics curriculum in Iran: An application of the delphi technique. Crescent J Med Biol Sci. 2020;7(2):201–6.

Reason: Wrong outcome

1. Ahmad S, Jenkins MF, Alexander SD. Artificial intelligence for nursing practice and management: Current and potential research and education. CIN: Computers, Informatics, Nursing. 2022;40(3):139–44. doi: 10.1097/CIN.0000000000000871

Reason: Wrong outcome

1. Ahmadi M, Sheikhtaheri A, Tahmasbi F, Eslami Jahromi M, Rangraz Jeddi F. A competency framework for Ph.D. programs in health information management. Int J Med Inform.2022;168:104906. doi: 10.1016/j.ijmedinf.2022.104906

Reason: Wrong outcome

1. Ahmed TJ, Baig M, Bashir MA, Gazzaz ZJ, Butt NS, Khan SA. Knowledge, attitudes, and perceptions related to telemedicine among young doctors and nursing staff at the King Abdul-Aziz University Hospital Jeddah, KSA. Niger J Clin Pract. 2021;24(4):464-469. doi: 10.4103/njcp.njcp_34_20

Reason: Wrong population

1. Ahonen O, Kinnunen UM, Lejonqvist GB, Apkalna B, Viitkar K, Saranto K. Identifying biomedical and health informatics competencies in higher education curricula. Stud Health Technol Inform. 2018;251:261-264. doi: 10.3233/978-1-61499-880-8-261

Reason: Wrong population

1. Ahonen O, Kouri P, Kinnunen UM, Junttila K, Liljamo P, Arifulla D, Saranto K. The development process of eHealth strategy for nurses in Finland. Stud Health Technol Inform. 2016;225:203-207.

Reason: Wrong population

1. Ahonen O, Kinnunen UM, Lejonqvist GB, Apkalna B, Viitkar K, Saranto K. Identifying biomedical and health informatics competencies in higher education curricula. Data, informatics and technology: An inspiration for improved healthcare.

Reason: Duplicate

1. Airth-Kindree N, Vandenbark RT. Mobile applications in nursing education and practice. Nurse Educator. 2014;39(4):166–9. doi: 10.1097/nne.0000000000000041

Reason: Wrong outcome

1. Ajayi NA. Impact of health informatics on nurses’ computer skills and the role of the library. Electron Libr. 2013;31(2):157-166. doi: 10.1108/02640471311312357

Reason: Wrong population

1. Akman A, Erdemir F, Agah Tekindal M. Psychometric properties and reliability of the Turkish version of the technology attitudes survey and nursing students’ attitudes toward technology. Int J Caring Sci. 2014;7(2):415-425.

Reason: Wrong population

1. Akman A, Erdemir F, Agah Tekindal M. Psychometric properties and reliability of the Turkish version of the technology attitudes survey and nursing students’ attitudes toward technology. Int J Caring Sci. 2014;7(2):415-425.

Reason: Duplicate

1. Al-Hawamdih S, Ahmad MM. Examining the relationship between nursing informatics competency and the quality of information processing. Comput Inform Nurs. 2018;36(3):154-160. doi: 10.1097/CIN.0000000000000379

Reason: Wrong population

1. Almalki M, Househ M, Alhefzi M. Developing a Saudi health informatics competency framework: A comparative assessment. Stud Health Technol Inform. 2019;264:1101-1105. doi: 10.3233/SHTI190396

Reason: Wrong population

1. Ali S, Kleib M, Paul P, Petrovskaya O, Kennedy M. Compassionate nursing care and the use of digital health technologies: A scoping review. *Int J Nurs Stud*. 2022;127(104161):104161. doi:10.1016/j.ijnurstu.2021.104161

Reason: Review

1. Alipour J, Payandeh A. Assessing the level of digital health literacy among healthcare workers of teaching hospitals in the southeast of Iran. Inform Med Unlocked. 2022;29:100868. doi: 10.1016/j.imu.2022.100868

Reason: Wrong population

1. Alshammari F, Pasay-An E, Indonto MCL. Competencies in nursing informatics in the Saudi Arabian context: A sequential explanatory study. Philipp J Nurs. 2017;87(2):45-55.

Reason: Wrong population

1. Ammenwerth E, Hackl WO. Topics for continuous education in nursing informatics: Results of a survey among 280 Austrian nurses. Stud Health Technol Inform. 2019;260:162-169. doi: 10.3233/978-1-61499-971-3-162

Reason: Wrong population

1. Ammenwerth E, Hackl WO. Topics for continuous education in nursing informatics: Results of a survey among 280 Austrian nurses. Stud Health Technol Inform. 2019;260:162-169.

Reason: Duplicate

1. Anderberg P, Bjorling G, Stjernberg L, Bohman D. Analyzing nursing students’ relation to electronic health and technology as individuals and students and in their future career (the eNursEd Study): Protocol for a longitudinal study. JMIR Res Protoc. 2019;8(10):e14643. doi: 10.2196/14643

Reason: Wrong outcome

1. Anderson C, Sensmeier J, Kwiatkoski T. Results of the 2020 HIMSS nursing informatics workforce survey-growth in education and leadership. Comput Inform Nurs. 2020;38(9):431–432. doi:10.1097/CIN.0000000000000679
2. Reason: Wrong concept
3. Anderson VM, Tracy MF. Capturing original preanesthetic comprehensive history and physical assessments with the use of video telehealth: A quality improvement project for student nurse anesthetists. J Perianesth Nurs. 2023;9610507. doi: 10.1016/j.jopan.2023.07.021

Reason: Wrong concept

1. Anderson M, Kelly LR, Lauderman C, Bench JR, Miller S, Harlan MD. Using sharable electronic databases to develop nursing case studies to simulate clinical experiences. Nurse Educator. 2022;47(5):E122–E123. doi:10.1097/NNE.0000000000001157

Reason: Wrong outcome

1. Ang RJ. Content management and nursing informatics education. Can J Nurs Inform. 2022;17(2):1–3.

Reason: Wrong outcome

1. Anonymous. Section A. Professional nursing roles and nursing informatics competencies. Stud Health Technol Inform. 2017;232:7.

Reason: Wrong outcome

1. Anonymous. The Omaha system as a structured instrument for bridging nursing informatics with public health nursing education: A feasibility study. CIN: Computers, Informatics, Nursing. 2018;36(6):314–5. doi: 10.1097/01.NCN.0000532438.43897.1a

Reason: Wrong outcome

1. Ariosto DA, Harper EM, Wilson ML, Hull SC, Nahm ES, Sylvia ML. Population health: A nursing action plan. JAMIA Open. 2018;1(1):7–10. doi: 10.1093/jamiaopen/ooy003

Reason: Wrong concept

1. Atique S, Bautista JR, Block LJ, Lee JJ, Lozada‐Perezmitre E, Nibber R, et al. A nursing informatics response to COVID‐19: Perspectives from five regions of the world. J Adv Nurs. 2020;76(10):2462–2468. doi: 10.1111/jan.14417

Reason: Wrong concept

1. Austria PJM. Exploring the influence of informatics in Nursing: The Filipino student nurses perspective. Online J Nurs Inform. 2017;21(2):2–1.

Reason: Wrong concept

1. Bakas T, Sampsel D, Israel J, Chamnikar A, Ellard A, Clark JG, Ulrich MG, Vanderelst D. Satisfaction and technology evaluation of a telehealth robotic program to optimize healthy independent living for older adults. J Nurs Scholarsh. 2018;50(6):666-675. doi: 10.1111/jnu.12436

Reason: Wrong population

1. Bahrami M, Hadadgar A, Fuladvandi M. Designing virtual patients for education of nursing students in cancer course. Iran J Nurs Midwifery Res. 2021;26(2):133–6. doi: 10.4103/ijnmr.IJNMR_327_20

Reason: Wrong outcome

1. Bartz CC. Leadership strategies for improved nursing synergy between informatics and telehealth. Stud Health Technol Inform. 2014;201:227–232. doi: 10.3233/978-1-61499-415-2-227

Reason: Wrong concept

1. Baillie L, Chadwick S, Mann R, Brooke-Read M. A survey of student nurses’ and midwives’ experiences of learning to use electronic health record systems in practice. Nurse Educ Pract. 2013;13(5):437–441.doi:1016/j.nepr.2012.10.003

Reason: Wrong concept

1. Banbury A, Taylor ML, Gray LC, Reid N, Smith AC. Sustaining and expanding telehealth activity: Training requirements for Australian residential aged care front-line staff. PEC Innovation. 2023;2. doi: 10.1016/j.pecinn.2022.100109

Reason: Wrong population

1. Barbosa S de FF. Competencies related to informatics and information management for practicing nurses and nurses leaders in Brazil and South America. Stud Health Technol Inform. 2017;232:77-85.

Reason: Wrong population

1. Beaney P, Hatfield R, Hughes A, Schmid M, Chambers R. Creating digitally ready nurses in general practice. Nurs Manag (Harrow). 2019;26(3):27-35. doi: 10.7748/nm.2019.e1840

Reason: Wrong population

1. Benavente A, Urra E, Hullin C, Almond H. Pilot research to develop a nursing digital health capability framework in Chile. Stud Health Technol Inform. 2021;284:186-188. doi: 10.3233/SHTI210700

Reason: Wrong population

1. Bergren MD, Maughan ED, VanDenBrink R, Foster B, Carveth L. Nursing informatics and school nursing: Specialists wanted. NASN Sch Nurse. 2020;35(4):208-210. doi: 10.1177/1942602X20928347

Reason: Wrong population

1. Belnap J. Scratching the surface: Using informatics in education. J Doctoral Nurs Pract. 2016;9(1):164–6. doi: 10.1891/2380-9418.9.1.164

Reason: Wrong outcome

1. Bickford CJ. The specialty of nursing informatics: New scope and standards guide practice. CIN: Computers, Informatics, Nursing. 2015;33(4):129–31. doi: 10.1097/CIN.0000000000000150

Reason: Wrong outcome

1. Bickford CJ. NI continuing education: Replicating a U.S. model in other countries? Stud Health Technol Inform. 2016;225:678–9.

Reason: Wrong outcome

1. Bickford CJ. The professional association’s perspective on nursing informatics and competencies in the US. Stud Health Technol Inform. 2017;232:62-68.

Reason: Wrong population

1. Bickford CJ. Update: NI scope and standards of practice, competencies, and certification. Stud Health Technol Inform. 2016;225:746–7.

Reason: Wrong outcome

1. Bichel-Findlay J. Digital health. Aust Midwifery News. 2021;23(1):28-29.

Reason: Wrong study design

1. Blahna BL. TECH NOTES. How nurses can collaborate with EHR design. Nurs. 2015;45(12):25-26. doi: 10.1097/01.NURSE.0000473404.97294.02

Reason: Wrong population

1. Borycki EM, Cavan Frisch N. Nursing informatics. Can J Nurs Res. 2013;45(1):92-94. doi: 10.1177/084456211304500108

Reason: Wrong study design

1. Borycki EM, Cummings E, Kushniruk AW, Saranto K. Integrating health information technology safety into nursing informatics competencies. Stud Health Technol Inform. 2017;232:222–8.

Reason: Wrong outcome

1. Borycki EM, Foster J. A comparison of Australian and Canadian informatics competencies for undergraduate nurses. Stud Health Technol Inform. 2014;201:349–55. doi: 10.3233/978-1-61499-415-2-349

Reason: Wrong outcome

1. Borycki EM, Foster J, Sahama T, Frisch N, Kushniruk AW. Developing national level informatics competencies for undergraduate nurses: Methodological approaches from Australia and Canada. Stud Health Technol Inform. 2013;183:345–9.

Reason: Wrong outcome

1. Borycki EM, Kushniruk AW. Educational electronic health records at the University of Victoria: Challenges, recommendations and lessons learned. Stud Health Technol Inform. 2019;265:74-79. doi: 10.3233/SHTI190141

Reason: Wrong population

1. Bozikov J, Hercigonja Szekeres M. Training of students for critical evaluation of mobile health applications. Stud Health Technol Inform. 2017;245:1320.

Reason: Wrong population

1. Bratton-Robinson J. Mental Health and Technology: The use of mobile mental health apps in an undergraduate psychiatric-mental health nursing course. Nurse Educator. 2023:10-97. doi:10.1097/nne.0000000000001384

Reason: Wrong outcome

1. Brixey JJ. Health informatics competencies, workforce and the DNP: Why connect these “dots”? Stud Health Technol Inform. 2016;225:750-752.

Reason: Wrong population

1. Brownie SM, Chalmers LM, Broman P, Andersen P. Evaluating an undergraduate nursing student telehealth placement for community-dwelling frail older people during the COVID-19 pandemic. J Clin Nurs. 2023;32(1-2):147-162. doi:10.1111/jocn.16208.

Reason: Duplicate

1. Brown J, Morgan A, Mason J, Pope N, Bosco AM. Student nurses’ digital literacy levels: Lessons for curricula. CIN: Computers, Informatics, Nursing. 2020;38(9):451–8. doi: 10.1097/CIN.0000000000000615

Reason: Wrong outcome

1. Brown Wilson C, Slade C, Wong WYA, Peacock A. Health care students experience of using digital technology in patient care: A scoping review of the literature. *Nurse Educ Today*. 2020;95:104580. doi:10.1016/j.nedt.2020.104580

Reason: Review

1. Burgos D, Lopez-Serrano A, Palmisano S, Timmins F, Connolly M. Digital competencies for nurses: Tools for responding to spiritual care needs. Healthcare (Basel). 2022;10(10). doi: 10.3390/healthcare10101966

Reason: Wrong population

1. Bryant LE, Whitehead DK, Kleier JA. Development and testing of an instrument to measure informatics knowledge, skills, and attitudes among undergraduate nursing students. Online J Nurs Inform. 2016;20(2):3-3.

Reason: Wrong concept

1. Butler M. Teaching the new HIM: Educators integrating informatics, data analytics, and information governance into HIM programs. J AHIMA. 2017;88(5):16-19.

Reason: Wrong study design

1. Carroll K. Transforming the art of nursing: Telehealth technologies. Nurs Sci Q. 2018;31(3):230–2. doi: 10.1177/0894318418774930

Reason: Wrong outcome

1. Carrington JM, Tiase VL, Estrada N, Shea KD. Nursing education focus of nursing informatics research in 2013. Nurs Admin Q. 2014;38(2):189–91.doi: 10.1097/NAQ.0000000000000024

Reason: Wrong outcome

1. Carrington JM. Trends in nursing informatics research and the importance of the nurse administrator. Nurs Adm Q. 2016;40(2):184-185. doi: 10.1097/NAQ.0000000000000163

Reason: Wrong concept

1. Carrington JM, Love R. A model to guide telehealth research quality improvement, practice, and education. Stud Health Technol Inform. 2021;284:369-373. doi: 10.3233/SHTI210749

Reason: Wrong concept

1. Carrington JM, Love R. A model to guide telehealth research quality improvement, practice, and education...15th International Congress on Nursing Informatics (Online), August 23-September 2, 2021. Stud Health Technol Inform. 2021;284:369-373. doi: 10.3233/SHTI210749

Reason: Duplicate

1. Carrington JM, Tiase V, Estrada N, Shea KD, Dudding KM, Finley BA, Nibbelink C, Rasmussen RJ, Roberts ML. Nursing informatics research and emerging trends in 2015. CIN: Comput Inform Nurs. 2016;34(7):284-286. doi: 10.1097/cin.0000000000000278

Reason: Wrong concept

1. Çetin S, Ergün MA, Tekindal MA, Tekindal B, Tekindal M. A qualitative evaluation of the knowledge levels of nurses regarding informatics and health informatics: The case of Atatürk training and research hospital. Int J Caring Sci. 2015;8(3):555-566.

Reason: Wrong population

1. Chambers R, Hughes A, Beaney P, Schmid M. You too can be a digital practice nurse champion. Pract Nurse. 2018;48(6):11–5.

Reason: Wrong outcome

1. Chambers R, McKinney R. Digital by choice: Becoming part of a digitally ready general practice team. Prim Health Care. 2018;28(7):22–7. doi: 10.7748/phc.2018.e1502

Reason: Wrong outcome

1. Chen Y, Cai Z, Lin B, Yan L, Zheng W, Kuo M-C, Hubner U, Chang P. Developing a professional-practice-model-based nursing organizational informatics competency model. Int J Med Inform. 2022;166:104840. doi: 10.1016/j.ijmedinf.2022.104840

Reason: Wrong outcome

1. Chen Y, Yan L, Zheng W, Lin B, Wu L, Wu Z, Chang P. Development of Nursing Informatics Competencies Evaluation Index System of Clinical Nurses. Stud Health Technol Inform. 2021;284:177-178. doi: 10.3233/SHTI210696

Reason: Wrong outcome

1. Chia-Jung H, Tzu-Chi H, Pei-Chang H. Exploring nursing students’ learning experiences in improving their health information technology competency in interdisciplinary cooperation: An innovative pilot study. Stud Health Technol Inform. 2021;284:181–3. doi: 10.3233/SHTI210698

Reason: Wrong outcome

1. Chia-Hui Liu, Ting-Ting Lee, Mills ME. The experience of informatics nurses in Taiwan. J Prof Nurs. 2015;31(2):158-164.

Reason: Wrong population

1. Chi KL, Chiou SF. Needs Assessment and System Design of an Extracorporeal Membrane Oxygenation Simulation Learning System...15th International Congress on Nursing Informatics (Online), August 23-September 2, 2021. Stud Health Technol Inform. 2021;284:537-538.doi: 10.3233/SHTI210791

Reason: Wrong concept

1. Chike-Harris KE, Durham C, Logan A, Smith G, DuBose-Morris R. Integration of telehealth education into the health care provider curriculum: A review. *Telemed J E Health*. 2021;27(2):137-149. doi:10.1089/tmj.2019.0261

Reason: Review

1. Chike-Harris KE, Garber K, Derouin A. Telehealth educational resources for graduate nurse faculty. Nurse Educator. 2021;46(5):295.

Reason: Wrong outcome

1. Clarke-Darrington J, McDonald T, Ali P. Digital capability: An essential nursing skill for proficiency in a post-COVID-19 world. *Int Nurs Rev*. 2023;70(3):291-296. doi:10.1111/inr.12839

Reason: Review

1. Choi J. Comparative assessment of informatics competencies in three undergraduate programs. Online J Nurs Inform. 2012;16(2):1–8.

Reason: Wrong outcome

1. Choi J, Bakken S. Validation of the self-assessment of nursing informatics competencies scale among undergraduate and graduate nursing students. J Nurs Educ. 2013;52(5):275–82. doi: 10.3928/01484834-20130412-01

Reason: Wrong outcome

1. Choi J, De Martinis JE. Nursing informatics competencies: Assessment of undergraduate and graduate nursing students. J Clin Nurs (John Wiley & Sons, Inc.). 2013;22(13–14):1970–6. doi: 10.1111/jocn.12188

Reason: Wrong outcome

1. Choi J, Zucker DM. Self-assessment of nursing informatics competencies for Doctor of Nursing practice students. J Prof Nurs. 2013;29(6):381-7.

Reason: Wrong outcome

1. Choi J, Bove L, Tarte V. Nursing informatics competency after experiencing simulated electronic health records: Descriptive study...15th International Congress on Nursing Informatics (Online), August 23-September 2, 2021. Stud Health Technol Inform. 2021;284:143-147. doi: 10.3233/SHTI210686

Reason: Wrong study design

1. Choi KS. Virtual Reality in Nursing: Nasogastric Tube Placement Training Simulator...16 World Congress of Medical and Health Informatics: Precision Healthcare Through Informatics (MedInfo2017), Hangzhou, China, 2017. Stud Health Technol Inform. 2017;245:1298-1298. doi: [10.3233/978-1-61499-830-3-1298](https://doi.org/10.3233/978-1-61499-830-3-1298)

Reason: Wrong concept

1. Choi M, Park JH, Lee HS. Assessment of the need to integrate academic electronic medical records into the undergraduate clinical practicum: A focus group interview. CIN: Comput Inform Nurs. 2016;34(6):259-265. doi: 10.1097/CIN.0000000000000244

Reason: Wrong concept

1. Chung SY, Staggers N. Measuring nursing informatics competencies of practicing nurses in Korea: Nursing Informatics Competencies Questionnaire. Comput Inform Nurs. 2014;32(12):596-605. doi: 10.1097/CIN.0000000000000114

Reason: Wrong population

1. Conte G, Arrigoni C, Magon A, Stievano A, Caruso R. Embracing digital and technological solutions in nursing: A scoping review and conceptual framework. *Int J Med Inform*. 2023;177(105148):105148. doi:10.1016/j.ijmedinf.2023.105148

Reason: Review

1. Collins S, Po-Yin Yen, Phillips A, Kennedy MK. Nursing informatics competency assessment for the nurse leader. JONA. 2017;47(4):212-218. doi: 10.1097/NNA.0000000000000467

Reason: Wrong population

1. Collins S, Yen P-Y, Phillips A, Kennedy MK. Nursing informatics competency assessment for the nurse leader: The Delphi Study. J Nurs Adm. 2017;47(4):212-218. doi: 10.1097/NNA.0000000000000467

Reason: Wrong population

1. Condor DF, Sanchez Alvarez K, Bidman AA. Nursing Informatics Training in Undergraduate Nursing Programs in Peru. *Stud Health Technol Inform*. 2018;250:81.

Reason: Review

1. Crabtree A. Incorporating telehealth in curriculum. J Allied Health. 2021;50(1):92-92.

Reason: Full-text unavailable

1. Creedon SA, Cummins AM. Development of a blended model of teaching and learning for nursing students on rostered placement to ensure competence in information and communication technology for professional practice in Ireland. CIN: Computers, Informatics, Nursing. 2012;30(5):274–9. doi: 10.1097/ncn.0b013e31823eb652

Reason: Wrong outcome

1. Cummings E, Borycki EM, Madsen I. Teaching nursing informatics in Australia, Canada and Denmark. Stud Health Technol Inform. 2015;218:39–44. doi: 10.3233/978-1-61499-574-6-39

Reason: Wrong outcome

1. Cummings E, Shin EH, Mather C, Hovenga E. Embedding nursing informatics education into an Australian undergraduate nursing degree. Stud Health Technol Inform. 2016;225:329–33. doi: [10.3233/978-1-61499-658-3-329](https://doi.org/10.3233/978-1-61499-658-3-329)

Reason: Wrong outcome

1. Cummins MR. Nursing informatics and learning health system. CIN: Computers, Informatics, Nursing. 2014;32(10):471–4. doi: 10.1097/CIN.0000000000000109

Reason: Wrong outcome

1. Cummings E, Moran G, Woods L, Almond H, Procter P, Makeham M, Dobroff N, Griffin K, Reeves J, Nowlan S, Ryan A, Schaper L. Methodology for the development of the Australian national nursing and midwifery digital health capability framework. Stud Health Technol Inform. 2021;284:135-142. doi: 10.3233/SHTI210685

Reason: Wrong population

1. Curioso WH, Pena-Ayudante WR, Oscuvilca-Tapia E. COVID-19 reveals the urgent need to strengthen nursing informatics competencies: A view from Peru. Inform Health Soc Care. 2021;46(3):229–33. doi: 10.1080/17538157.2021.1941974

Reason: Wrong outcome

1. Curran V, Gustafson DL, Simmons K, Lannon H, Wang C, Garmsiri M, Fleet L, Wetsch L. Adult learners’ perceptions of self-directed learning and digital technology usage in continuing professional education: An update for the digital age. J Adult Contin Educ. 2019;25(1):74-93. doi: 10.1177/1477971419827318

Reason: Wrong population

1. Darvish A, Bahramnezhad F, Keyhanian S, Navidhamidi M. The role of nursing informatics on promoting quality of health care and the need for appropriate education. Glob J Health Sci. 2014;6(6):11-18. doi: 10.5539/gjhs.v6n6p11

Reason: Wrong study design

1. De Fatima Faria BARBOSA S. Competencies related to informatics and information management for practicing nurses and nurses leaders in Brazil and South America. Stud Health Technol Inform. 2017;232:77-85. doi: 10.3233/978-1-61499-738-2-77

Reason: Wrong population

1. De Leeuw JA, Woltjer H, Kool RB. Identification of factors influencing the adoption of health information technology by nurses who are digitally lagging: In-depth interview study. J Med Internet Res. 2020;22(8):e15630. doi: 10.2196/15630

Reason: Wrong population

1. De Gagne JC, Bisanar WA, Makowski JT, Neumann JL. Integrating informatics into the BSN curriculum: a review of the literature. *Nurse Educ Today*. 2012;32(6):675-682. doi:10.1016/j.nedt.2011.09.003

Reason: Review

1. Doorenbos AZ, Min Kyeong Jang, Hongjin Li, Lally RM. eHealth Education: Methods to enhance oncology nurse, patient, and caregiver teaching. Clin J Oncol Nurs. 2020;24:42-48. doi: 10.1188/20.CJON.S1.42-48

Reason: Wrong population

1. Eardley DL, Krumwiede KA, Secginli S, Garner L, DeBlieck C, Cosansu G, Nahcivan NO. The Omaha System as a structured instrument for bridging nursing informatics with public health nursing education: A feasibility study. CIN: Computers, Informatics, Nursing. 2018;36(6):275–83. doi: 10.1097/CIN.0000000000000425

Reason: Wrong outcome

1. Egbert N, Thye J, Hackl WO, Müller-Staub M, Ammenwerth E, Hübner U. Competencies for nursing in a digital world. Methodology, results, and use of the DACH-recommendations for nursing informatics core competency areas in Austria, Germany, and Switzerland. Inform Health Soc Care. 2019;44(4):351–75. doi: 10.1080/17538157.2018.1497635

Reason: Wrong outcome

1. Egbert N, Thye J, Schulte G, Liebe J-D, Hackl WO, Ammenwerth E, Hubner U. An iterative methodology for developing national recommendations for nursing informatics curricula. Studies in Health Technology and Informatics. 2016;228:660–664.

Reason: Wrong study design

1. Egilsdottir HO, Heyn LG, Brembo EA, Byermoen KR, Moen A, Eide H. Configuration of mobile learning tools to support basic physical assessment in nursing education: Longitudinal participatory design approach. JMIR Mhealth Uhealth. 2021;9(1):e22633. doi: 10.2196/22633

Reason: Wrong concept

1. Ellis BS, Quayle S, Bailey I, Tishkovskaya S, Spencer J, Richardson R. Students’ perceptions on their use of an EHR: pilot questionnaire study. BMJ Health Care Inform. 2020;27(3). doi: 10.1136/bmjhci-2020-100163

Reason: Wrong outcome

1. Estes K, Gilliam E, Knapfel S, Chanmi LEE, Skiba D. Discovering eHealth Technology: An Innovative Interprofessional Graduate Student Learning Experience. Stud Health Technol Inform. 2016;225:242-246. doi: 10.3233/978-1-61499-658-3-242

Reason: Wrong population

1. Farzandipour M, Mohamadian H, Akbari H, Safari S, Jabali MS. Self-assessment of nursing informatics competencies in hospitals. Online J Nurs Informatics. 2020;24(2):1-1.

Reason: Wrong outcome

1. Farzandipour M, Mohamadian H, Akbari H, Safari S, Sharif R. Designing a national model for assessment of nursing informatics competency. BMC Med Inform Decis Mak. 2021;21(1):1-12. doi: [10.1186/s12911-021-01405-0](https://doi.org/10.1186/s12911-021-01405-0)

Reason: Wrong population

1. Fields W. Musings on teaching an undergraduate nursing informatics course. Online J Nurs Informatics. 2015;19(3):1-2.

Reason: Wrong outcome

1. Feldthouse DM, Jacques DP, Fenelon L, Robertiello G, Pasklinsky N, Fletcher J, Groom LL, Doty GR, Squires AP. Implementing an academic electronic health record in nursing education. Journal of Informatics Nursing. 2022;7(2):37–42.

Reason: Full-text unavailable

1. Fernandez-Marcelo PG, Ho BL, Faustorilla JFJ, Evangelista AL, Pedrena M, Marcelo A. Emerging eHealth directions in the Philippines. Yearbook of Medical Informatics. 2012;7(9312666):144–152.

Reason: Wrong study design

1. Firuzi Z, Sabet MS, Jafaraghaee F, Jafari H, Leyli EK, Karkhah S, Ghazanfari MJ. Effect of a forensic nursing virtual education course on knowledge and clinical decision-making of master’s nursing students in Iran: A non-equivalent control group pre- and post-test study. J Educ Eval Health Prof. 2022;19:20. doi: 10.3352/jeehp.2022.19.20

Reason: Wrong outcome

1. Furukawa M, Harrison L, Mugford Y, Pollack E, Selsky S, Wellbaum D. Nursing informatics fellowship: Developing future nurse informaticists at UCLA Health. J Inform Nurs. 2020;5(1):24-41.

Reason: Wrong population

1. Firuzi Z, Sabet MS, Jafaraghaee F, Jafari H, Leyli EK, Karkhah S, Ghazanfari MJ. Effect of a forensic nursing virtual education course on knowledge and clinical decision-making of master’s nursing students in Iran: A non-equivalent control group pre- and post-test study. J Educ Eval Health Prof. 2022;19:20. doi: 10.3352/jeehp.2022.19.20

Reason: Wrong outcome

1. Foronda C. Teaching ethics in healthcare technology-not science fiction. CIN: Comput Inform Nurs. 2021;39(5):231-236. doi: 10.1097/CIN.0000000000000766

Reason: Wrong concept

1. Foster M, Sethares K. Current Strategies to Implement Informatics into the Nursing Curriculum: An Integrative Review. OJNI. 2017;21(3).

Reason: Review

1. Foster M, Lioce L, Howell Adams M. Telehealth in nursing education: A systematic review. *J Nurs Educ*. 2021;60(11):633-635. doi:10.3928/01484834-20210913-06

Reason: Review

1. Forman TM, Flores D, Miller AS. An Integrative Literature Review of the Use of Electronic Health Records for Clinical Nursing Education. J Inform Nurs. 2020;5(1):28-39, 43-44.

Reason: Review

1. Fulton CR, Meek JA, Walker PH. Faculty and organizational characteristics associated with informatics/health information technology adoption in DNP programs. J Prof Nurs. 2014;30(4):292-299.

Reason: Wrong outcome

1. Fujino Y, Kawamoto R. Effect of information and communication technology on nursing performance. Comput Inform Nurs. 2013;31(5):244-250. doi: 10.1097/NXN.0b013e3182842103

Reason: Wrong population

1. Furukawa M, Harrison L, Mugford Y, Pollack E, Selsky S, Wellbaum D. Nursing informatics fellowship: Developing future nurse informaticists at UCLA Health. J Inform Nurs. 2020;5(1):24-41.

Reason: Wrong population

1. Gadd C, Delaney CW, de Fatima Marin H, Greenwood K, Williamson JJ. Accelerating the global workforce demand for nurse Informaticians: Advanced Health Informatics Certification (AHIC). Studies in Health Technology and Informatics. 2016;225(ck1, 9214582):697–699.

Reason: Wrong study design

1. Gartz J, O’Rourke J. Telehealth educational interventions in nurse practitioner education: An integrative literature review: An integrative literature review. *J Am Assoc Nurse Pract*. 2020;33(11):872-878. doi:10.1097/JXX.0000000000000488

Reason: Review

1. Gardner CL, Jones SJ. Utilization of Academic Electronic Medical Records in Undergraduate Nursing Education. Online J Nurs Inform. 2012;16(2):31-37.

Reason: Wrong concept

1. Georgsson M. A nursing informatics research career in the making—Personal reflections and insights. Online J Nurs Informatics. 2018;22(2):2-1.

Reason: Wrong outcome

1. Georgsson M. Uses and advantages of eLearning and distance pedagogy in nursing informatics education. Online J Nurs Informatics. 2019;23(2):7-7.

Reason: Wrong outcome

1. Georgsson M. Rethinking the provision of education during the COVID-19 pandemic—online learning and nursing informatics. Online J Nurs Informatics. 2020;24(3):7-7.

Reason: Wrong outcome

1. Gibson NA, Arends R, Hendrickx L. Tele-U to Tele-ICU: Telehealth Nursing Education. Crit Care Nurse. 2021;41(5):34-39. doi:10.4037/ccn2021109

Reason: Wrong concept

1. Grzybowski D. Standards, information governance, informatics—Essential components of HIM workforce education. J AHIMA. 2017;88(5):48-50.

Reason: Wrong population

1. Glinkowski W, Pawlowska K, Kozlowska L. Telehealth and telenursing perception and knowledge among university students of nursing in Poland. Telemed J E Health. 2013;19(7):523-529. doi: 10.1089/tmj.2012.0217

Reason: Wrong concept

1. Grady J. CE: Telehealth: A case study in disruptive innovation. Am J Nurs. 2014;114(4):38-7. doi: 10.1097/01.NAJ.0000445682.52553.89

Reason: Wrong concept

1. Gonen A, Sharon D, Offir A, Lev-Ari L. How to enhance nursing students’ intention to use information technology: The first step before integrating it in nursing curriculum. Comput Inform Nurs. 2014;32(6):286-293. doi: 10.1097/CIN.0000000000000064

Reason: Wrong outcome

1. Gonzalez ZA, Schachner MB, Tattone MA, Benitez SE. Changing Educational Paths in an Informatics Course According to the Needs and Expectations of Nursing Degree Students. Stud Health Technol Inform. 2016;225:324-328.

Reason: Wrong outcome

1. Goossen W. Informatics competencies in connected health: Annotated bibliography. In: NI 2016, Switzerland. Studies in Health Technology & Informatics. 2017;232:241–251. doi: 10.3233/978-1-61499-738-2-241

Reason: Wrong study design

1. Grundland B, Kulasegaram K, Prucnal K, Freeman R. Implementation and evaluation of an innovative virtual care curriculum. Medical Education. 2022;56(11):1133–1134. doi: 10.1111/medu.14920

Reason: Wrong population

1. Gulzar S, Khoja S, Sajwani A. Experience of nurses with using eHealth in Gilgit-Baltistan, Pakistan: A qualitative study in primary and secondary healthcare. BMC Nurs. 2013;12:6. doi: [10.1186/1472-6955-12-6](https://doi.org/10.1186/1472-6955-12-6).

Reason: Wrong population

1. Hah H, Goldin D. Exploring care providers’ perceptions and current use of telehealth technology at work, in daily life, and in education: Qualitative and quantitative study. JMIR Med Educ. 2019;5(1):e13350. doi: 10.2196/15087

Reason: Wrong population

1. Hah H, Goldin D, Ha S. The association between willingness of frontline care providers’ to adaptively use telehealth technology and virtual service performance in provider-to-provider communication: Quantitative study. J Med Internet Res. 2019;21(8):e15087.

Reason: Wrong population

1. Harerimana A, Mtshali NG. Types of ICT applications used and the skills’ level of nursing students in higher education: A cross-sectional survey. Int J Afr Nurs Sci. 2019;11:100163. doi: 10.1016/j.ijans.2019.100163

Reason: Wrong concept

1. Harerimana A, Wicking K, Biedermann N, Yates K. Integrating nursing informatics into undergraduate nursing education in Africa: A scoping review. *Int Nurs Rev*. 2021;68(3):420-433. doi:10.1111/inr.12618

Reason: Review

1. Harerimana A, Wicking K, Biedermann N, Yates K. Nursing informatics in undergraduate nursing education in Australia before COVID-19: A scoping review. *Collegian*. 2022;29(4):527-539. doi:10.1016/j.colegn.2021.11.004

Reason: Review

1. Herrera A, Foronda C. From Bedside to Webside: Telehealth Education for Doctoral Nursing Students. J Doctor Nurs Pract. 2022;15(3):165-172. doi: 10.1891/JDNP-2021-0049

Reason: Full-text unavailable

1. Hamilton D. Can a reset of digital literacy standards improve nursing practice? Br J Nurs. 2018;27(13):768-768. doi: 10.12968/bjon.2018.27.13.768

Reason: Wrong study design

1. Hamilton H, Iradukunda F, Aselton P. The integration of telehealth in nursing education: A new frontier. Journal of Informatics Nursing. 2021;6(1):18–25.

Reason: Wrong study design

1. Hebda TL, Calderone TL. Informatics competencies for healthcare professionals: The Technology Informatics Guiding Education Reform (TIGER) Initiative model. Drug Metab Drug Interact. 2012;27(3):145-149. doi: 10.1515/dmdi-2012-0013

Reason: Wrong outcome

1. Herbert VM, Connors H. Integrating an academic electronic health record: Challenges and success strategies. Comput Inform Nurs. 2016;34(8):345-376. doi:10.1097/cin.0000000000000264

Reason: Wrong outcome

1. Hawkins SY. Telehealth nurse practitioner student clinical experiences: An essential educational component for today’s health care setting. Nurse Educ Today. 2012;32(8):842-845. doi: 10.1016/j.nedt.2012.03.008

Reason: Wrong outcome

1. Honey M, Kim Young, Cowls H. Virtual student collaboration: Connecting student health professionals. In: MEDINFO 2019, the 17th World Congress on Medical and Health Informatics, August 25-30, 2019, Lyon, France. Studies in Health Technology & Informatics. 2019;264:1935–1936. doi: 10.3233/SHTI190721

Reason: Wrong study design

1. Honey M, Collins E, Britnell S. Identifying how to support nurse educators nationally to teach nursing informatics. Stud Health Technol Inform.2021;284:124-129. doi: 10.3233/SHTI210683

Reason: Wrong outcome

1. Honey MLL, Skiba DJ, Procter P, Foster J, Kouri P, Nagle LM. Nursing informatics competencies for entry to practice: The perspective of six countries. Stud Health Technol Inform. 2017;232:51-61.

Reason: Wrong outcome

1. Honey M, Westbrooke L. Evolving national strategy driving nursing informatics in New Zealand. Stud Health Technol Inform. 2016;225:183-187.

Reason: Wrong outcome

1. Howard EV, Bishop-Clark C, Evans DM, Rose AW. Developing a bachelor’s program in health information technology. *Information Systems Education Journal*. 2013;11(1):33-40. Reason: Wrong outcome
2. Honey M, Procter P. The Shifting Sands of Nursing Informatics Education: From Content to Connectivity. Stud Health Technol Inform. 2017;232:31-40.

Reason: Wrong concept

1. Honey M, Wright J. Nurses developing confidence and competence in telehealth: Results of a descriptive qualitative study. Contemp Nurse. 2018;54(4-5):472-482. doi: 10.1080/10376178.2018.1530945

Reason: Wrong population

1. Hong KJ, Park NL, Heo SY, Jung SH, Lee YB, Hwang JH. Effect of e-Health Literacy on COVID-19 Infection-Preventive Behaviors of Undergraduate Students Majoring in Healthcare. Healthc (Basel). 2021;9(5). doi:10.3390/healthcare9050573

Reason: Wrong concept

1. Horton A, Roser S. Evaluating nurse data navigation training program: A quality improvement project. In: 15th International Congress on Nursing Informatics (Online), August 23-September 2, 2021. Studies in Health Technology & Informatics. 2021;284:179–180. doi: 10.3233/SHTI210697

Reason: Wrong study design

1. Holt KA, Overgaard D, Engel LV, Kayser L. Health literacy, digital literacy and eHealth literacy in Danish nursing students at entry and graduate level: A cross sectional study. BMC Nurs. 2020;19:22. doi: 10.1186/s12912-020-00418-w

Reason: Wrong outcome

1. Hsien-Cheng Lin, Meng-Hsiang Hsu, Chen-Wei Yang. The Influences of computer system success and informatics competencies on organizational impact in nursing environments. Comput Inform Nurs. 2014;32(2):90-99. doi:10.1097/CIN.0000000000000010

Reason: Wrong outcome

1. Hsieh CJ, Hsu TZ, Hsu PC. Exploring Nursing Students’ Learning Experiences in Improving Their Health Information Technology Competency in Interdisciplinary Cooperation: An Innovative Pilot Study...15th International Congress on Nursing Informatics (Online), August 23-September 2,. Stud Health Technol Inform. 2021;284:181-183. doi: 10.3233/SHTI210698

Reason: Wrong concept

1. Hubner U, Shaw T, Thye J, Egbert N, Marin H, Ball M. Towards an international framework for recommendations of core competencies in nursing and inter-professional informatics: The TIGER competency synthesis project. Stud Health Technol Inform. 2016;228:655-659.

Reason: Wrong outcome

1. Hubner U, Thye J, Shaw T, Elias B, Egbert N, Saranto K, Babitsch B, Procter P, Ball MJ. Towards the TIGER international framework for recommendations of core competencies in health informatics 2.0: Extending the scope and the roles. Stud Health Technol Inform. 2019;264:1218-1222. doi: 10.3233/SHTI190420

Reason: Wrong outcome

1. Hübner U, Ball M, Fátima Marin H de, Chang P, Wilson M, Anderson C. Towards implementing a global competency-Based nursing and clinical informatics curriculum: Applying the TIGER initiative. Stud Health Technol Inform. 2016;225:762-764.

Reason: Wrong outcome

1. Hudgins T, Camp-Spivey L, Lee S. Leveraging Innovation to Design a Psychiatric Mental Health Simulation for Undergraduate Nursing Students During the COVID-19 Global Pandemic. Nurs Educ Perspect. 2023;44(1):59-60. doi: 10.1097/01.NEP.0000000000000922

Reason: Wrong concept

1. Hussey P, Adams E, Shaffer FA. Nursing informatics and leadership, an essential competency for a global priority: eHealth. Nurse Leader. 2015;13(5):52-57. doi: 10.1016/j.mnl.2015.07.002

Reason: Wrong population

1. Hullin C. Nursing informatics education: Latino America & Caribe. Studies in Health Technology & Informatics. 2016;225:729–731. doi: [10.3233/978-1-61499-658-3-729](https://doi.org/10.3233/978-1-61499-658-3-729)

Reason: Wrong study design

1. Hui KY, Haines C, Bammann S, et al. To what extent is telehealth reported to be incorporated into undergraduate and postgraduate allied health curricula: A scoping review. *PLoS One*. 2021;16(8):e0256425. doi:10.1371/journal.pone.0256425

Reason: Review

1. Jarva E, Oikarinen A, Andersson J, Tuomikoski A-M, Kääriäinen M, Meriläinen M, Mikkonen K. Healthcare professionals’ perceptions of digital health competence: A qualitative descriptive study. Nurs Open. 2022;9(2):1379-1393. doi: 10.1002/nop2.1184

Reason: Wrong population

1. Jang SM, Kim J. A study on nursing informatics competence of clinical nurses: Applying focus group interview. J Korean Acad Soc Nurs Educ. 2020;26(3):299-310. doi: 10.5977/jkasne.2020.26.3.299

Reason: Wrong population

1. Jenkins ML, Davis A. Transforming nursing documentation. The 17th World Congress of Medical and Health Informatics, 25-30 August 2019, Lyon, France. Stud Health Technol Inform. 2019;264:625-8. doi: 10.3233/SHTI190298

Reason: Wrong outcome

1. Jensen R, De Souza Guedes E, Leite MMJ. Informatics competencies essential to decision making in nursing management. Rev Esc Enferm USP. 2016;50(1):109-17. doi: 10.1590/S0080-623420160000100015

Reason: Wrong outcome

1. Jeon E, Peltonen L-M, Block L, Ronquillo C, Tayaben JL, Nibber R, Pruinelli L, Perezmitre EL, Sommer J, Topaz M, Eler GJ, Shishido HY, Wardaningsih S, Sutantri S, Ali S, Alhuwail D, Abd-Alrazaq A, Akhu-Zaheya L, Lee Y-L, Shao-Hui SHU. Emergency remote learning in nursing education during the COVID-19 pandemic. In: Medical Informatics Europe, Public Health and Informatics Conference (Virtual), 29-31 May 2021. Studies in Health Technology & Informatics. 2021;281:942–946. doi: 10.3233/SHTI210317

Reason: Wrong study design

1. Johnson D, Gatewood E, Ling A, Kuo AC. Teleprecepting: A timely approach to clinical education during COVID-19. J Am Assoc Nurse Pract. 2021;34(1):153-159. doi: 10.1097/JXX.0000000000000567

Reason: Wrong concept

1. Johnson DS, Ling A, Melino K. Exploring Readiness for Teleprecepting in Psychiatric Mental Health Nurse Practitioner Training. J Am Psychiatr Nurses Assoc. 2021;27(2):169-173. doi: 10.1177/1078390320948127

Reason: Wrong concept

1. Jouparinejad S, Foroughameri G, Khajouei R, Farokhzadian J. Improving the informatics competency of critical care nurses: Results of an interventional study in the southeast of Iran. BMC Med Inform Decis Mak. 2020;20(1). doi: 10.1186/s12911-020-01244-5

Reason: Wrong population

1. Ju-Young H, Lee SY. The relationship between the subjective health status, e-health literacy, health literacy and health promoting behavior in undergraduate nursing students. Medico-Legal Update. 2019;19(1):641-5. doi: [10.5958/0974-1283.2019.00114.2](https://doi.org/10.5958/0974-1283.2019.00114.2)

Reason: Wrong outcome

1. Kaltoft MK, Nielsen JB, Salkeld G, Dowie J. Enhancing informatics competency under uncertainty at the point of decision: A knowing about knowing vision. Stud Health Technol Inform. 2014;205:975-979.

Reason: Wrong concept

1. Kaynar NS, Secginli S, West K. Psychometric testing of the Turkish version of the technology informatics guiding educational reform-based assessment of nursing informatics competencies tool. Comput Inform Nurs. 2020;38(11):572-578. doi: 10.1097/CIN.0000000000000671

Reason: Wrong concept

1. Kazawa K, Teramoto C, Azechi A, Satake H, Moriyama M. Undergraduate nursing students’ learning experiences of a telehealth clinical practice program during the COVID-19 pandemic: A qualitative study. Nurse Educ Today. 2022;111:105297. doi: 10.1016/j.nedt.2022.105297

Reason: Wrong concept

1. Kaas MJ. Will We Be Ready? Preparing psychiatric-mental health nurses for future practice. Journal of the American Psychiatric Nurses Association. 2020;26(1):112–119. doi: 10.1177/1078390319878767

Reason: Wrong study design

1. Kawi J. Educational innovations leveraging technology for nurse practitioner students to learn pain management. Pain Management Nursing. 2023;24(2):240–241. doi: 10.1016/j.pmn.2023.02.028

Reason: Wrong study design

1. Kaihlanen A, Elovainio M, Virtanen L, Kinnunen UM, Vehko T, Saranto K, Heponiemi T. Nursing informatics competence profiles and perceptions of health information system usefulness among registered nurses: A latent profile analysis. J Adv Nurs (John Wiley & Sons, Inc). 2023;79(10):4022-4033. doi: 10.1111/jan.15718

Reason: Wrong population

1. Kaihlanen A-M, Gluschkoff K, Kinnunen UM, Saranto K, Ahonen O, Heponiemi T. Nursing informatics competencies of Finnish registered nurses after national educational initiatives: A cross-sectional study. Nurse Educ Today. 2021;106. doi: 10.1016/j.nedt.2021.105060

Reason: Wrong population

1. Kaihlanen A-M, Gluschkoff K, Laukka E, Heponiemi T. The information system stress, informatics competence and well-being of newly graduated and experienced nurses: A cross-sectional study. BMC Health Serv Res. 2021;21(1). doi: 10.1186/s12913-021-07132-6

Reason: Wrong population

1. Kaminski J. Nursing informatics summer reading suggestions. Online J Nurs Informatics. 2017;21(2):6-1.

Reason: Wrong outcome

1. Kannry J, Sengstack P, Thyvalikakath TP, Poikonen J, Middleton B, Payne T, Lehmann CU. The Chief Clinical Informatics Officer (CCIO): AMIA task force report on CCIO knowledge, education, and skill set requirements. Appl Clin Inform. 2016;7(1):143-176. doi: 10.4338/ACI-2015-12-R-0174

Reason: Wrong population

1. Kerr MJ, Honey ML, Krzyzanowski B. Geo-spatial informatics in international public health nursing education. Studies in Health Technology and Informatics. 2016;225:983–984.

Reason: Wrong study design

1. Kennedy MA, Moen A. Nurse leadership and informatics competencies: Shaping transformation of professional practice. Stud Health Technol Inform. 2017;232:197-206.

Reason: Wrong population

1. Kennedy MK, Vrana-Bossart M, Henry A, Goldsmith DM, Phillips A. New England Nursing Informatics Consortium and CIN: Computers, Informatics, Nursing Partner to Offer a Virtual Journal Club With Continuing Education!. Comput Inform Nurs. 2022;40(3):145-146. doi: 10.1097/CIN.0000000000000905

Reason: Wrong population

1. Khairat S, Feldman SS, Rana A, Faysel M, Purkayastha S, Scotch M, Eldredge C. Foundational domains and competencies for baccalaureate health informatics education. Journal of the American Medical Informatics Association. 2023;30(10):1599-607

Reason: Wrong concept

1. Kirkman T, Brown N, DeFoor M, Seay A, Darby W. Enriching nursing simulation with a threefold hybrid approach. Clin Simul Nurs. 2022;68:34-41. doi: 10.1016/j.ecns.2022.04.008

Reason: Wrong outcome

1. Kim HN. A conceptual framework for interdisciplinary education in engineering and nursing health informatics. Nurse Educ Today. 2019;74:91-93.doi: 10.1016/j.nedt.2018.12.010

Reason: Wrong population

1. Kim IJ, Kim MS, Jeon MK. Exploring Nursing Students’ Perspectives on Telenursing Using Q-methodology. Comput Inform Nurs. 2021;39(12):1007-1016. doi: 10.1097/CIN.0000000000000767

Reason: Wrong concept

1. Kim J. Development of nursing informatics education model for strengthening information processing capability. *Journal Of Engineering And Applied Sciences*. 2017;12(Specialissue2):6356-6361.

Reason: Wrong concept

1. Kim S, Jeon J. Factors influencing eHealth literacy among Korean nursing students: A cross‐sectional study. Nurs Health Sci. 2020;22(3):667-674. doi: 10.1111/nhs.12711

Reason: Wrong concept

1. Kinnunen UM, Rajalahti E, Cummings E, Borycki EM. Curricula challenges and informatics competencies for nurse educators. Stud Health Technol Inform. 2017;232:41-48.

Reason: Wrong population

1. Kinnunen UM, Heponiemi T, Rajalahti E, Ahonen O, Korhonen T, Hyppönen H. Factors related to health informatics competencies for nurses—Results of a national electronic health record survey. Comput Inform Nurs. 2019;37(8):420-429. doi: 10.1097/CIN.0000000000000511

Reason: Wrong concept

1. Kleib M, Arnaert A, Nagle LM, et al. Digital health education and training for undergraduate and graduate nursing students: a scoping review protocol. *JBI Evid Synth*. 2023;21(7):1469-1476. doi:10.11124/JBIES-22-00266

Reason: Review

1. Kleib M, Chauvette A, Furlong K, Nagle L, Slater L, McCloskey R. Approaches for defining and assessing nursing informatics competencies: a scoping review: A scoping review. *JBI Evid Synth*. 2021;19(4):794-841. doi:10.11124/JBIES-20-00100

Reason: Review

1. Kleib M. Emerging leaders and informatics. Nurs Leadersh (Tor Ont). 2013;26(3):21-3.

Reason: Wrong outcome

1. Kleib M, Chauvette A, Nagle L. Are Alberta nurses prepared for digital health? Alberta RN. 2018;74(3):40-1.

Reason: Wrong outcome

1. Kleib M, Nagle L. Psychometric properties of the Canadian nurse informatics competency assessment scale. Comput Inform Nurs. 2018;36(7):359-65. doi: 10.1097/CIN.0000000000000437

Reason: Wrong outcome

1. Kleib M, Nagle LM. The psychometric properties of version 2 of the Canadian nurse informatics competency assessment scale. Comput Inform Nurs. 2023;41(3):153-61. doi: 10.1097/CIN.0000000000000940

Reason: Wrong outcome

1. Kleib M, Shaben T, Zimka O, Kwan J, Alberta Nursing Informatics Group. Informatics is considered a core competency for safe clinical practice. Alberta RN. 2013;69(3):12.

Reason: Wrong outcome

1. Kleib M, Nagle L. Factors associated with Canadian nurses’ informatics competency. Comput Inform Nurs. 2018b;36(8):406-415. doi: 10.1097/CIN.0000000000000434

Reason: Wrong population

1. Kleib M, Nagle L. Development of the Canadian nurse informatics competency assessment scale and evaluation of Alberta’s registered nurses’ self-perceived informatics competencies. Comput Inform Nurs. 2018a;36(7):350-358. doi: 10.1097/CIN.0000000000000435

Reason: Wrong concept

1. Kowitlawakul Y, Chan SWC, Wang L, Wang W. Exploring faculty perceptions towards electronic health records for nursing education. Int Nurs Rev. 2014;61(4):499-506. doi: 10.1111/inr.12141

Reason: Wrong outcome

1. Kowitlawakul Y, Chan SWC, Pulcini J, Wang W. Factors influencing nursing students’ acceptance of electronic health records for nursing education (EHRNE) software program. Nurse Educ Today. 2015;35(1):189-194. doi: 10.1016/j.nedt.2014.05.010

Reason: Wrong concept

1. Konstantinidis ST, Sisi LI, Traver V, Zary N, Bamidis PD. Actions to empower digital competences in healthcare workforce: A qualitative approach. Stud Health Technol Inform. 2017;238:253-256. doi: 10.3233/978-1-61499-781-8-253

Reason: Wrong population

1. Kokol P, BlaŽUn H, VoŠNer J, Saranto K. Nursing informatics competencies: Bibliometric analysis. Studies in Health Technology & Informatics. 2014;201:342–348. doi: 10.3233/978-1-61499-415-2-342

Reason: Wrong study design

1. Kokol P, Vošner HB. Nursing informatics research: A bibliometric analysis of funding patterns. Online Journal of Nursing Informatics. 2017;21(2).

Reason: Wrong study design

1. Kokol P, Vošner HB, Železnik D, Vošner J, Saranto K. Bibliometric patterns of research literature production on nursing informatics competence. Journal of Nursing Education. 2015;54(10):565–571. doi:10.3928/01484834-20150916-04

Reason: Wrong study design

1. Kristová J, Bachratá Z, Slezáková Z, Miklovičová E. Implementation of telenursing in the Slovak Republic. Nurs 21st Century. 2021;20(3):216-20. doi: 10.2478/pielxxiw-2021-0028

Reason: Wrong outcome

1. Kujala S, Heponiemi T, Hilama P. Clinical leaders’ self-perceived eHealth competencies in the implementation of new eHealth services. Stud Health Technol Inform. 2019;264:1253-1257. doi: 10.3233/SHTI190427

Reason: Wrong population

1. Kujala S, Heponiemi T, Hilama P. Clinical Leaders’ Self-Perceived eHealth Competencies in the Implementation of New eHealth Services. In: The 17th World Congress of Medical and Health Informatics, 25-30 August 2019, Lyon, France. Stud Health Technol Inform. 2019;264:1253-1257. doi: 10.3233/SHTI190427

Reason: Duplicate

1. Kupferschmid B, Creech C, Lesley M, Schoville R. Informatics experience can help predict doctor of nursing practice student mastery of informatics competencies. Comput Inform Nurs. 2020;38(11):590-6. doi: 10.1097/CIN.0000000000000656

Reason: Wrong outcome

1. Kupferschmid B, Creech C, Lesley M, Filter M, Aplin-Kalisz C. Evaluation of doctor of nursing practice students' competencies in an online informatics course. Journal of Nursing Education. 2017;56(6):364-7. doi:10.3928/01484834-20170518-09

Reason: Wrong concept

1. Lee JJ, Clarke CL. Nursing students’ attitudes towards information and communication technology: An exploratory and confirmatory factor analytic approach. J Adv Nurs. 2015;71(5):1181-1193. doi: 10.1111/jan.12611

Reason: Wrong concept

1. Lee JS. Implementation and evaluation of a virtual reality simulation: Intravenous injection training system. Int J Environ Res Public Health. 2022;19(9). doi: 10.3390/ijerph19095439

Reason: Wrong outcome

1. Levy S. Commentary: Assessing nurses’ informatics competency and identifying its related factors. J Res Nurs. 2019;24(7):539-540. doi: 10.1177/1744987119839870

Reason: Wrong concept

1. Lewis A, Block L, Peltonen L, Pruinelli L, Topaz M, Lozada Perezmitre E. Emerging Professional’s View of the Landscape: Nursing Informatics Curriculum, Competencies, and Career Opportunities. Stud Health Technol Inform. 2018;250:60-61. doi: 10.3233/978-1-61499-872-3-60

Reason: Wrong concept

1. Le Y, Cao S, Wang M, Lin X, Qian B. A bibliometric and visualized analysis of nursing informatics competencies in China (2000-2020). In: 15th International Congress on Nursing Informatics (Online), August 23-September 2, 2021. Studies in Health Technology & Informatics. 2021;284:403–407. doi: 10.3233/SHTI210759

Reason: Wrong study design

1. Lekalakala-Mokgele E, Lowane MP, Mogale NM. Knowledge, perceptions and attitudes of eHealth and health technology among nursing students from Gauteng Province, South Africa. Healthcare (Basel). 2023;11(12). doi: 10.3390/healthcare11121672

Reason: Wrong outcome

1. Lilly KD, Eldridge C. Healthcare informatics in 21st-century nursing: Are dermatology nurses prepared? J Dermatol Nurses’ Assoc. 2012;4(3):188-194. doi: 10.1097/JDN.0b013e318256b9dc

Reason: Wrong population

1. Liu C-H, Lee T-T, Mills ME. The experience of informatics nurses in Taiwan. J Prof Nurs. 2015;31(2):158-164. doi: 10.1016/j.profnurs.2014.09.005

Reason: Wrong population

1. Liu S, Zheng T, Fang J, Liu J. Assessment of the Chinese literature for nursing informatics education initiatives. Studies in Health Technology and Informatics. 2021;284(ck1, 9214582):220–222. doi:10.3233/SHTI210708

Reason: Wrong study design

1. Liu J, Liu S, Fu M, Fang J. Nursing Students’ Perceptions Towards Clinical Informatics Course. Stud Health Technol Inform. 2021;284:169-170. doi: 10.3233/SHTI210692

Reason: Wrong concept

1. Liu J, Liu S, Yang Y, Fang J. Research themes and hotspots in nursing informatics education based co-word analysis. In: 15th International Congress on Nursing Informatics (Online), August 23-September 2, 2021. Studies in Health Technology & Informatics. 2021;284:74–76. doi: 10.3233/SHTI210670

Reason: Wrong study design

1. Lima LDG, Tomaschewski-Barlem JG, Paloski GR, Barlem ELD, Rocha LP, Castanheira JS. The performance of nursing students in a tele-health service during the COVID-19 pandemic. Rev Gaucha Enferm. 2021;42(8504882, rev):e20200483. doi: 10.1590/1983-1447.2021.20200483

Reason: Wrong concept

1. Lo B, Nagle LM, White P, Kleib M, Kennedy MA, Strudwick G. Digital and informatics competencies: Requirements for nursing leaders in Canada. Healthc Manage Forum. 2021;34(6):320-325. doi: 10.1177/08404704211015428

Reason: Wrong population

1. Lokmic-Tomkins Z, Brar S, Lin N, Khor M, Mathews K, Lawlor K. Advancing nursing informatics through clinical placements: pilot study. Stud Health Technol Inform. 15th International Congress on Nursing Informatics (Online), August 23-September 2, 2021;284:98-102. doi: 10.3233/SHTI210678

Reason: Wrong outcome.

1. Lokmic-Tomkins Z, Marriott P, Tuddenham A, Martin J. Using real-time data to develop and improve teaching of clinical skills in virtual nursing simulation laboratories during COVID-19 pandemic. Stud Health Technol Inform. 15th International Congress on Nursing Informatics (Online), August 23-September 2, 2021;284:153-7. doi:10.3233/SHTI210688

Reason: Wrong outcome

1. Lokmic-Tomkins Z, Cochrane L, Celeste T, Burnie M. An Interdisciplinary Partnership Approach to Improving the Digital Literacy Skills of Nursing Students to Become Digitally Fluent, Work-Ready Graduates. Stud Health Technol Inform. 2021;284:103-107. 10.3233/SHTI210679

Reason: Wrong concept

1. Longhini J, Rossettini G, Palese A. Digital health competencies among health care professionals: Systematic review. *J Med Internet Res*. 2022;24(8):e36414. doi:10.2196/36414

Reason: Review

1. Lopez KD, Castner J, Pruinelli L, Schoville R, Piscotty RJ, Farag A, Abusalem S, Monsen KA. Shared Passion at the Nexus of Nursing Informatics, Systems, Policy, and Research: Midwest Nursing Research Society Advances the State of the Science. Comput Inform Nurs. 2018;36(1):5-7. doi: 10.1097/CIN.0000000000000412

Reason: Wrong concept

1. Madsen I, Cummings E, Borycki EM, Lacroix P. Developing a framework for teaching nursing informatics internationally. Studies in Health Technology & Informatics. 2016;225:783–785. doi: [10.3233/978-1-61499-658-3-783](https://doi.org/10.3233/978-1-61499-658-3-783)

Reason: Wrong study design

1. McKay C, Vanaskie K. Partnering for success: The role of the nurse leader in health information technology implementation for coordination of care. Nurse Leader. 2018;16(6):385-388. doi: 10.1016/j.mnl.2018.07.012

Reason: Wrong population

1. Mataxen PA, Webb LD. Telehealth nursing: More than just a phone call. Nursing. 2019;49(4):11-13. doi: 10.1097/01.NURSE.0000553272.16933.4b

Reason: Wrong concept

1. McCabe C, Timmins F. Embracing healthcare technology – What is the way forward for nurse education? Nurse Educ Pract. 2016;21:104-106. doi: 10.1016/j.nepr.2016.10.007

Reason: Wrong concept

1. McCormick KA, Calzone KA. Genetic and genomic competencies for nursing informatics internationally. Stud Health Technol Inform. 2017;232:152-164. doi: 10.3233/978-1-61499-738-2-152

Reason: Wrong concept

1. McCormick KA, Gugerty B, Sensmeier J. A comparison of professional informatics-related competencies and certifications. Online J Nurs Inform. 2017;21(1):1-1.

Reason: Wrong concept

1. McGonigle D, Hunter K, Hebda T. How can we promote nursing informatics? Online J Nurs Inform. 2013;17(1):30-30. doi: 10.1016/j.aorn.2014.06.012

Reason: Wrong concept

1. McGonigle D, Hunter K, Sipes C, Hebda T. Why nurses need to understand nursing informatics. AORN J. 2014;100(3):324-327.

Reason: Wrong concept

1. McNelis AM, Horton-Deutsch S, Friesth BM. Improving quality and safety in graduate education using an electronic student tracking system. Arch Psychiatr Nurs. 2012;26(5):358-363. doi: 10.1016/j.apnu.2012.06.006

Reason: Wrong concept

1. Mack A, O’Donnell M, Henning A, Bernstein SL. Development of a hospital-academic collaboration to implement an interprofessional telehealth breastfeeding support group. J Interprof Care. 2023;9205811:1-4. doi: 10.1080/13561820.2023.2240851

Reason: Wrong concept

1. Madsen I, Cummings E, Borycki EM. Current status for teaching nursing informatics in Denmark, Canada, and Australia. Medinfo. 2015:1016–1016.doi: 10.3233/978-1-61499-564-7-1016

Reason: Wrong study design

1. Mamta. Nursing informatics: The future now. Nurs J India. 2014;105(5):198-9.

Reason: Wrong outcome

1. Mantas J, Hasman A. IMIA educational recommendations and nursing informatics. In: NI 2016, Switzerland. Studies in Health Technology & Informatics. 2017;232:20–30. doi: 10.3233/978-1-61499-738-2-20

Reason: Wrong study design

1. Mann EG, Medves J, Vandenkerkhof EG. Accessing best practice resources using mobile technology in an undergraduate nursing program: A feasibility study. Comput Inform Nurs. 2015;33(3):122-8. doi: 10.1097/CIN.0000000000000135

Reason: Wrong outcome

1. Masouras PP. ICT & nursing informatics skills in Cyprus’ universities undergraduate nursing curricula. International Journal of Learning. 2016;2(2):172–177. doi: 10.18178/IJLT.2.2.172-177

Reason: Wrong study design

1. Martinez-Ortigosa A, Martinez-Granados A, Gil-Hernández E, Rodriguez-Arrastia M, Ropero-Padilla C, Roman P. Applications of Artificial Intelligence in Nursing Care: A Systematic Review. *Journal of Nursing Management*. July 2023:1-12. doi:10.1155/2023/3219127

Reason: Review

1. Martin-Sanchez F, Rowlands D, Schaper L, Hansen D. The Australian health informatics competencies framework and its role in the Certified Health Informatician Australasia (CHIA) Program. Stud Health Technol Inform. 2017;245:783-787. doi: 10.3233/978-1-61499-830-3-783

Reason: Wrong population

1. Martzoukou K, Luders ES, Mair J, Kostagiolas P, Johnson N, Work F, Fulton C. A cross-sectional study of discipline-based self-perceived digital literacy competencies of nursing students. J Adv Nurs. 2023;7609811:h3l. doi: 10.1111/jan.15801

Reason: Wrong outcome

1. Mather C, Cummings E, Gale F. Mobile learning in nursing: Tales from the profession. Stud Health Technol Inform. 2018;252(ck1, 9214582):112-7.

Reason: Wrong outcome

1. Mather C, Cummings E. Moving past exploration and adoption: Considering priorities for implementing mobile learning by nurses. Stud Health Technol Inform. 2017;241:63-68. doi: 10.3233/978-1-61499-794-8-63

Reason: Wrong population

1. Meum TT, Koch TB, Briseid HS, Vabo GL, Rabben J. Perceptions of digital technology in nursing education: A qualitative study. Nurse Educ Pract. 2021;54: N.PAG-N.PAG. doi: 10.1016/j.nepr.2021.103136

Reason: Wrong concept

1. Mills E, Procter P. Undergraduate nurses’ attitude change to health technology implementation. Stud Health Technol Inform. 2021;284:191-3. doi: 10.3233/SHTI210702

Reason: Wrong outcome

1. Miller C, Rose A, Roach A, Lloyd-Penza M. Implementation of a telephone-triage simulation-based experience into undergraduate nursing curriculum. Clin Simul Nurs. 2023;75:20-24. doi: 10.1016/j.ecns.2022.11.005

Reason: Wrong concept

1. Mills J, Fox J, Damarell R, Tieman J, Yates P. Palliative care providers’ use of digital health and perspectives on technological innovation: A national study. BMC Palliat Care. 2021;20(1):124. doi: 10.1186/s12904-021-00822-2

Reason: Wrong concept

1. Mollart L. Nursing undergraduates’ perception of preparedness using patient electronic medical records in clinical practice. Aust J Adv Nurs. 2021;38(2):44-51. doi: 10.37464/2020.382.282

Reason: Wrong concept

1. Monsen KA, Bush RA, Jones J, Manos EL, Skiba DJ, Johnson SB. Alignment of American Association of Colleges of Nursing graduate-level nursing informatics competencies with American Medical Informatics Association health informatics core competencies. Comput Inform Nurs. 2019;37(8):396-404. doi: 10.1097/CIN.0000000000000537

Reason: Wrong outcome

1. Monsen KA, Pesut DJ. Pilot test of a theory-based instrument to measure nursing informatics leadership skills. Nurs Leadersh (Tor Ont). 2020;33(1):100-111. doi: 10.12927/cjnl.2020.26188

Reason: Wrong concept

1. Murphy J. Engaging nurses in the design and adoption of mHealth tools for care coordination. Stud Health Technol Inform. 2015;225:765-767. doi: 10.3233/978-1-61499-658-3-765

Reason: Wrong concept

1. Murphy J, Goossen W. Introduction: Forecasting informatics competencies for nurses in the future of connected health. Stud Health Technol Inform. 2017;232:1-6.

Reason: Wrong concept

1. Murphy J, Goossen W. Introduction: Forecasting informatics competencies for nurses in the future of connected health...NI 2016, Switzerland. Stud Health Technol Inform. 2017;232:1-6. doi: 10.3233/978-1-61499-738-2-1

Reason: Wrong concept.

1. Murphy J, Honey M, Newbold S, Weber P, Wu HY. Forecasting informatics competencies for nurses in the future of connected health. Stud Health Technol Inform. 2018;250:58-59. doi: [10.3233/978-1-61499-872-3-58](https://doi.org/10.3233/978-1-61499-872-3-58)

Reason: Wrong concept

1. Nagle L. Making informatics competency development explicit. Online J Nurs Informatics. 2013;17(1):31-32.

Reason: Wrong outcome

1. Nagle LM, Sermeus W, Junger A. Evolving role of the nursing informatics specialist. Stud Health Technol Inform. 2017;232(ck1, 9214582):212-21.

Reason: Wrong outcome

1. Nagle LM, Crosby K, Frisch N, Borycki E, Donelle L, Hannah K, Harris A, JettÉ S, Shaben T. Developing entry-to-practice nursing informatics competencies for registered nurses. Stud Health Technol Inform. 2014;201:356-363. doi: 10.3233/978-1-61499-415-2-356

Reason: Wrong population

1. Nazeha N, Pavagadhi D, Kyaw BM, Car J, Jimenez G, Tudor Car L. A digitally competent health workforce: Scoping review of educational frameworks. *J Med Internet Res*. 2020;22(11):e22706. doi:10.2196/22706

Reason: Review

1. Nelson R, Carter-Templeton HD. The nursing informatician’s role in mediating technology related health Literacies. Stud Health Technol Inform. 2016;225(ck1, 9214582):237-41.

Reason: Wrong outcome

1. Nes AAG, Steindal SA, Larsen MH, Heer HC, Lærum-Onsager E, Gjevjon ER. Technological literacy in nursing education: A scoping review. *J Prof Nurs*. 2021;37(2):320-334. doi:10.1016/j.profnurs.2021.01.008

Reason: Review

1. Newbold SK. What practicing nurses need to know about health information technology in order to practice today: Continuing education and certification. Stud Health Technol Inform. 2017;232:229-138. doi: 10.3233/978-1-61499-738-2-229

Reason: Wrong population

1. Nejadshafiee M, Bahaadinbeigy K, Kazemi M, Nekoei-Moghadam M. Telenursing: A step for care management in disaster and emergencies. J Educ Health Promot. 2020;9:204. doi: 10.4103/jehp.jehp_30_20

Reason: Wrong concept

1. O’Connor S, Yan Y, Thilo FJS, Felzmann H, Dowding D, Lee JJ. Artificial intelligence in nursing and midwifery: A systematic review. *J Clin Nurs*. 2023;32(13-14):2951-2968. doi:10.1111/jocn.16478

Reason: Review

1. O’Connor S, Hubner U, Shaw T, Blake R, Ball M. Time for TIGER to ROAR! technology informatics guiding education reform. Nurse Educ Today. 2017;58:78-81. doi: 10.1016/j.nedt.2017.07.014

Reason: Wrong outcome

1. O'Connor S, Gallagher J, Wamba N, Moyo C, Chirambo GB, O’Donoghue J. Establishing long-term nursing informatics capacity in Malawi, Africa. Stud Health Technol Inform. 2016;225:1013–1014. doi: 10.3233/978-1-61499-658-3-1013

Reason: Wrong concept

1. Oducado RMF, Moralista RB. Filipino nursing students’ eHealth literacy and criteria used for selection of health websites. Ann Trop Med Public Health. 2020;23(13):231343. doi: 10.36295/ASRO.2020.231343

Reason: Wrong concept

1. ONS Voice. What oncology nurses need to know about telehealth. ONS Voice. 2021;36(4):26-27.h

Reason: Wrong design

1. Paddock S, Norman C. Telehealth training: Interprofessional mixed reality simulation in a retrieval service setting. EMA Emerg Med Australas. 2022;34(1):134-6. doi: 10.1111/1742-6723.13891

Reason: Wrong outcome

1. Park HK, Park H. eHealth literacy skills among undergraduate nursing students in the U.S. and South Korea. Stud Health Technol Inform. 2016;225:899–900.

Reason: Wrong concept

1. Park HK, Jeong YW. Impact of nursing professionalism on perception of patient privacy protection in nursing students: Mediating effect of nursing informatics competency. Healthc (Basel). 2021;9(10). doi: 10.3390/healthcare9101364

Reason: Wrong concept

1. Park H, Lee E. Self-reported eHealth literacy among undergraduate nursing students in South Korea: A pilot study. Nurse Educ Today. 2015;35(2):408–413. doi: 10.1016/j.nedt.2014.10.013.

Reason: Wrong concept

1. Parker CD. TECH NOTES. Nursing informatics leadership: Helping craft the profession’s future. Nurs. 2014;44(12):23–24. doi: 10.1097/01.NURSE.0000456384.48273.a7

Reason: Wrong concept

1. Parreira P, Santos-Costa P, Graveto J, Ferreira PA, Salgueiro-Oliveira A, Sousa LB, et al. Personal and technological skills to coach people with noncommunicable diseases: Development and validation of a scale for nursing students. Heliyon. 2021;7(2):e06140. doi: 10.1016/j.heliyon.2021.e06140

Reason: Wrong concept

1. Peacock A, Slade C, Brown Wilson C. Nursing and midwifery students’ perspectives of using digital systems on placement: A qualitative study. J Adv Nurs. 2021;7609811:h3l. doi: 10.1111/jan.15091

Reason: Wrong outcome

1. Peltonen L-M, Nibber R, Block L, Ronquillo C, Lozada Perezmitre E, Lewis A, Alhuwail D, Ali S, Georgsson M, Jeon E, Tayaben JL, Lee Y-L, Kuo C-H, Shu S-H, Hsu H, Sommer J, Sarmiento RFR, Jung H, Eler GJ, Pruinelli L. Nursing informatics research trends: Findings from an international survey. Stud Health Technol Inform. 2021;284:344-9. doi: 10.3233/SHTI210741

Reason: Wrong outcome

1. Peltonen L-M, Nibber R, Lewis A, Block L, Pruinelli L, Topaz M, Perezmitre EL, Ronquillo C. Emerging professionals’ observations of opportunities and challenges in nursing informatics. Nurs Leadersh (Tor Ont). 2019;32(2):8-18. doi:10.12927/cjnl.2019.25965

Reason: Wrong outcome

1. Peltonen L-M, Sensmeier J, Saranto K, Newbold SK, Ramírez C. Supporting nursing informatics in practice—Lessons learned by nursing informatics pioneers. Stud Health Technol Inform. 2018;250:62-4. doi: 10.3233/978-1-61499-872-3-62

Reason: Wrong outcome

1. Perron T, Lebo C, Atkins R, Souza M, Jakubowski T. Telemedicine simulation in undergraduate nursing: Addressing social determinants of health. Nurse Educ. 2022;47(6):E150-E151. doi: 10.1097/NNE.0000000000001215

Reason: Wrong population

1. Phillips JM, Stalter AM, Goldschmidt KA, Ruggiero JS, Brodhead J, Bonnett PL, Provencio RA, Mckay M, Jowell V, Merriam DH, Wiggs CM, Scardaville DL. Using systems thinking to implement the QSEN informatics competency. J Contin Educ Nurs. 2019;50(9):392-397. doi: 10.3928/00220124-20190814-04

Reason: Wrong population

1. Phillips A, Yen P-Y, Kennedy M, Collins S. Opportunity and approach for implementation of a self-assessment tool: Nursing informatics competencies for nurse leaders (NICA-NL)...NI 2016, Switzerland. Stud Health Technol Inform. 2017;232:207-211. doi: 10.3233/978-1-61499-738-2-207

Reason: Wrong study design/conference proceedings

1. Phillips A, Yen P-Y, Kennedy MK, Collins S. Opportunity and approach for implementation of a self-assessment tool: Nursing Informatics Competencies for Nurse Leaders (NICA-NL). Stud Health Technol Inform. 2017;232:207-211.

Reason: Wrong population

1. Piercey C. Embedding health informatics skills into an undergraduate curriculum. Aust Nurs Midwifery J. 2015;23(2):41.

Reason: Wrong concept

1. Pontefract SK, Wilson K. Using electronic patient records: Defining learning outcomes for undergraduate education. BMC Med Educ. 2019a;19(1):30. doi: 10.1186/s12909-019-1466-5

Reason: Wrong population

1. Pordeli L. Informatics competency-based assessment: Evaluations and determination of nursing informatics competency gaps among practicing nurse informaticists. Online J Nurs Inform. 2018;22(3):5-5.

Reason: Wrong population

1. Ponchitra R. Nursing informatics. Nitte University Journal of Health Science. 2013;3(2):18–24.

Reason: Wrong study design

1. Powers K, Neustrup W, Thomas C, et al. Baccalaureate nursing students' experiences with multi-patient, standardized patient simulations using telehealth to collaborate. *J Prof Nurs*. 2020;36(5):292-300. doi:10.1016/j.profnurs.2020.03.013

Reason: Wrong outcome

1. Pramilaa R. Application of nursing informatics: Need to transform into reality. Int J Nurs Educ. 2013;5(1):152-6. doi: 10.5958/j.0974-9357.5.1.038

Reason: Wrong outcome

1. Prendergast M, Honey M. The barriers and facilitators for nurse educators using telehealth for education. MEDINFO 2019, the 17th World Congress on Medical and Health Informatics. Stud Health Technol Inform. 2019;264:1323-6. doi: 10.3233/SHTI190441

Reason: Wrong outcome

1. Procter P. A systematic approach to supporting faculty knowledge development in nursing and health informatics. Stud Health Technol Inform. 2021;284(ck1, 9214582):166-8. doi: 10.3233/SHTI210691

Reason: Wrong outcome

1. Procter P. A systematic approach to supporting faculty knowledge development in nursing and health informatics. In: 15th International Congress on Nursing Informatics (Online), August 23-September 2, 2021. Stud Health Technol Inform. 2021;284:166-168. doi:10.3233/SHTI210691

Reason: Duplicate

1. Pruinelli L. Nursing informatics competencies for emerging professionals: International leaders panel. Stud Health Technol Inform. 2016;225:774-6. doi: 10.3233/978-1-61499-658-3-774

Reason: Wrong outcome

1. Pullen RLJ, Silvers CA. Helping students embrace HIT. Nurs Manag. 2018;49(12):17–21. doi: 10.1097/01.NUMA.0000547841.89245.bd

Reason: Wrong concept

1. Quay C, Ramakrishnan A. Innovative use of virtual reality to facilitate empathy toward older adults in nursing education. Nurs Educ Perspect (Wolters Kluwer Health). 2023;44(5):300-2. doi: 10.1097/01.NEP.0000000000001174

Reason: Wrong outcome

1. Raghunathan K, McKenna L, Peddle M. Baseline evaluation of nursing students’ informatics competency for digital health practice: A descriptive exploratory study. Digital Health. 2023;9(101690863):20552076231179051. doi: 10.1177/20552076231179051

Reason: Wrong outcome

1. Raghunathan K, McKenna L, Peddle M. Use of academic electronic medical records in nurse education: A scoping review. *Nurse Educ Today*. 2021;101(104889):104889. doi:10.1016/j.nedt.2021.104889

Reason: Review

1. Rambur B, Palumbo MV, Nurkanovic M. Prevalence of telehealth in nursing: Implications for regulation and education in the era of value-based care. Policy Polit Nurs Pract. 2019;20(2):64-73. doi: 10.1177/1527154419836752

Reason: Wrong population

1. Ramukumba MM, El Amouri S. Nurses’ level of computer literacy, attitudes and responses regarding the electronic health record system in the United Arab Emirates. Afr J Nurs Midwifery. 2017;19(2). doi: 10.25159/2520-5293/1829

Reason: Wrong population

1. Randall K, Steinheider B, Isaacson M, Shortridge A, Byrd S, Ciro C, Ross H, Loving G. Measuring Knowledge, Acceptance, and Perceptions of Telehealth in an Interprofessional Curriculum for Student Nurse Practitioners, Occupational Therapists, and Physical Therapists. J Interact Learn Res. 2016;27(4):339-353.

Reason: Wrong population

1. Randall K, Steinheider B, Isaacson M, Shortridge A, Byrd S, Ciro C, Ross H, Loving G. Measuring knowledge, acceptance, and perceptions of telehealth in an interprofessional curriculum for student nurse practitioners, occupational therapists, and physical therapists. J Interact Learn Res. 2016;27(4):339-353.

Reason: Duplicate

1. Rajalahti E, Saranto K. Nursing informatics competencies are still challenging nurse educators. Stud Health Technol Inform. 2012;180:944-948.

Reason: Wrong outcome

1. Rajalahti E, Heinonen J, Saranto K. Developing nurse educators’ computer skills towards proficiency in nursing informatics. Inform Health Soc Care. 2014;39(1):47-66. doi: 10.3109/17538157.2013.834344

Reason: Wrong population

1. Rathnayake S, Senevirathna A. Self-reported eHealth literacy skills among nursing students in Sri Lanka: A cross-sectional study. Nurse Educ Today. 2019;78:50–56. doi:10.1016/j.nedt.2019.04.006

Reason: Wrong concept

1. Reid L, Button D, Breaden K, Brommeyer M. Nursing informatics and undergraduate nursing curricula: A scoping review protocol. *Nurse Educ Pract*. 2022;65(103476):103476. doi:10.1016/j.nepr.2022.103476

Reason: Review

1. Remus S, Kennedy MA. Innovation in transformative nursing leadership: Nursing informatics competencies and roles. Nurs Leadersh (Tor Ont). 2012;25(4):14–26.

Reason: Wrong concept

1. Risling T. Educating the nurses of 2025: Technology trends of the next decade. Nurse Educ Pract. 2017;22:89-92. doi: 10.1016/j.nepr.2016.12.007

Reason: Wrong outcome

1. Ronquillo C. Advancing nursing informatics in the next 5-10 years: What are the next steps? Stud Health Technol Inform. 2016;225:715-717. doi: 10.3233/978-1-61499-658-3-715

Reason: Wrong outcome

1. Ronquillo C, Topaz M, Pruinelli L, Peltonen L-M, Nibber R. Competency recommendations for advancing nursing informatics in the next decade: International survey results. Stud Health Technol Inform. 2017;232:119-129. doi: 10.3233/978-1-61499-738-2-119

Reason: Wrong outcome

1. Ross AM. Informatics assignment for graduate nursing practice study. J Nurs Educ. 2014;53(11):663-664. doi: [10.3928/01484834-20141022-11](https://doi.org/10.3928/01484834-20141022-11)

Reason: Wrong outcome

1. Ross P, Cross R. Rise of the e-Nurse: The power of social media in nursing. Contemp Nurse. 2019;55(2/3):211–220. doi: 10.1080/10376178.2019.1641419

Reason: Wrong concept

1. Rossler KL, Badowski D, Siegel S. The Presence of Simulated Telehealth in Prelicensure Nursing Education: A Scoping Review. *CLINICAL SIMULATION IN NURSING*. 2023;81:101415. doi:10.1016/j.ecns.2023.04.001

Reason: Review

1. Rouleau G, Gagnon M-P, Côté J, Hudson E, Payne-Gagnon J, Bouix-Picasso J, Duboi C-A. Effects of e-Learning and m-Learning on nursing care in a continuing education context: An overview of mixed method systematic reviews (Protocol). In: 16 World Congress of Medical and Health Informatics: Precision Healthcare Through Informatics (MedInfo2017). Studies in Health Technology & Informatics. 2017;245:1284–1284. doi: 10.3233/978-1-61499-830-3-1284

Reason: Wrong study design

1. Rutledge CM, O’Rourke J, Mason AM, Chike-Harris K, Behnke L, Melhado L, Downes L, Gustin T. Telehealth competencies for nursing education and practice: The four P’s of telehealth. Nurse Educ. 2021;46(5):300-305. doi: 10.1097/NNE.0000000000000988

Reason: Wrong outcome

1. Rutledge CM, Kott K, Schweickert PA, Poston R, Fowler C, Haney TS. Telehealth and eHealth in nurse practitioner training: Current perspectives. Advances in Medical Education and Practice. 2017;8(101562700):399–409. doi: 10.2147/AMEP.S116071

Reason: Wrong study design

1. Sahu M, Gandhi S, Sharma M, Mehrotra S. Nursing students’ perceptions regarding well-being and healthy use of digital technology: A qualitative thematic analysis. J Educ Health Promot. 2023;12. doi: 10.4103/jehp.jehp_1271_22

Reason: Wrong outcome

1. Sanches LMP, Alves DS, Lopes MHBM, Novaes MA. The practice of telehealth by nurses: An experience in primary healthcare in Brazil. Telemed J E Health. 2012;18(9):679-683. doi: 10.1089/tmj.2012.0011

Reason: Wrong population

1. Sanderson CD, Cox K, Disch J. Virtual nursing, virtual learning. Nurse Leader. 2020;18(2):142–146. doi: 10.1016/j.mnl.2019.12.005

Reason: Wrong study design

1. Saranto K, Ronquillo C, Velez O. Nursing competencies for multiple modalities of connected health technologies. Stud Health Technol Inform. 2017;232:172-182.

Reason: Wrong outcome

1. Sarath Rathnayake, Asela Senevirathna. Corrigendum to “Self-reported eHealth literacy skills among nursing students in Sri Lanka: A cross-sectional study”. Nurse Educ Today. 2019;79:210-210. doi: 10.1016/j.nedt.2019.05.029

Reason: Wrong outcome

1. Saratan C, Borycki EM, Kushniruk AW. Information management competencies for practicing nurses and new graduates. Knowl Manag E-Learn. 2015;7(3):378-394.

Reason: Wrong population

1. Sayaka Hoshino, Yoko Muranaka. Development of a tool for hospital nurses’ fatigue self-management that can be used for organizational management. Stud Health Technol Inform. 2021;284:175-176. doi: 10.3233/SHTI210695

Reason: Wrong outcome

1. Schooley B, Singh A, Hikmet N, Brookshire R, Patel N. Integrated digital patient education at the bedside for patients with chronic conditions: Observational study. JMIR Mhealth Uhealth. 2020;8(12):e22947. doi: 10.2196/22947

Reason: Wrong outcome

1. Schultz MA. The digital evolution in healthcare: What does it mean for nursing? J Nurs Adm. 2023;53(7–8):367–369. doi: 10.1097/NNA.0000000000001301

Reason: Wrong concept

1. Seibert K, Domhoff D, Huter K, Krick T, Rothgang H, Wolf-Ostermann K. Application of digital technologies in nursing practice: Results of a mixed methods study on nurses’ experiences, needs and perspectives. Z Evid Fortbild Qual Gesundhwes. 2020;158-159:94-106. doi: 10.1016/j.zefq.2020.10.010

Reason: Wrong outcome

1. Sensmeier J, Anderson C. Tracking the impact of nursing informatics. Nurs Made Incred Easy. 2021;19(1):49-53. doi: 10.1097/01.NME.0000723408.80090.47

Reason: Wrong outcome

1. Sensmeier J, Anderson C, Shaw T, Himss. International evolution of TIGER informatics competencies. Stud Health Technol Inform. 2017;232:69-76. doi: 10.3233/978-1-61499-738-2-69

Reason: Wrong outcome

1. Seo K, Min YH, Choi S-H, Lee H. Evaluation of the Korean version of the self-assessment of nursing informatics competencies scale. BMC Nurs. 2019;18(1):1-9. doi: 10.1186/s12912-019-0392-5

Reason: Wrong outcome

1. Sensmeier J, Anderson C, Shaw T. International evolution of TIGER informatics competencies. Studies in Health Technology and Informatics. 2015;232(ck1, 9214582):69–76.

Reason: Wrong study design

1. Sharpp TJ, Lovelace K, Cowan LD, Baker D. Perspectives of nurse managers on information communication technology and e‐Leadership. J Nurs Manag (John Wiley & Sons, Inc). 2019;27(7):1554-1562. doi: 10.1111/jonm.12845

Reason: Wrong population

1. Sharma S, Oli N, Thapa B. Electronic health-literacy skills among nursing students. Adv Med Educ Pract. 2019;10:527-532. doi: 10.2147/AMEP.S207353

Reason: Wrong outcome

1. Sharma K, Devi SD, Sharma B. The role of nursing informatics in healthcare sector. Journal of Critical Reviews. 2020;7(10):1364–1369. doi: 10.31838/jcr.07.10.268

Reason: Wrong study design

1. Shin EH, Cummings E, Ford K. A qualitative study of new graduates’ readiness to use nursing informatics in acute care settings: Clinical nurse educators’ perspectives. Contemp Nurse. 2018;54(1):64-76. doi: 10.1080/10376178.2017.1393317

Reason: Wrong outcome

1. Shirey MR, Bruck M, Patton E, Bowers D, Watts PI. COVID-19 telehealth fair partnership for capacity building in primary care nursing. Nurs Adm Q. 2022;7703976. doi: 10.1097/NAQ.0000000000000517

Reason: Wrong outcome

1. Shiferaw KB, Tilahun BC, Endehabtu BF. Healthcare providers’ digital competency: A cross-sectional survey in a low-income country setting. BMC Health Serv Res. 2020;20(1):1021. doi: [10.1186/s12913-020-05848-5](https://doi.org/10.1186/s12913-020-05848-5).

Reason: Wrong population

1. Shih DR, Rosenblum R. Attitudes and perceptions of advanced practice nurses towards health information technology and its effects on caring. Online J Nurs Inform. 2017;21(3).

Reason: Wrong population

1. Shorey S, Ang EN, Ng ED, Yap J, Lau LS, Chui CK, Chan YH. Evaluation of a theory-based virtual counseling application in nursing education. CIN: Computers, Informatics, Nursing. 2023:10-97. doi:10.1097/cin.0000000000000999

Reason: Wrong concept

1. Shuffitt JT, Effken J. Threading Informatics throughout Doctor of Nursing Practice (DNP) Curricula. Online Journal of Nursing Informatics. 2012;16(2):28–30.

Reason: Wrong study design

1. Shobuzawa S, Endo Y, Yamanouchi K. Availability of nursing informatics education for master’s programs in nursing administration at graduate schools of nursing in Japan. Stud Health Technol Inform. 2021;284:44-46. doi: 10.3233/SHTI210660

Reason: Duplicate

1. Simpson RL. Why not just any nurse can be a nurse informatician. Nurs Leadersh (Tor Ont). 2012;25(4):27-28.

Reason: Wrong outcome

1. Simpson RL. Chief nurse executives need contemporary informatics competencies. Nurs Econ$. 2013;31(6):277-288.

Reason: Wrong population

1. Sinard JH, Powell SZ, Karcher DS. Pathology training in informatics: Evolving to meet a growing need. Arch Pathol Lab Med. 2014;138(4):505–511. doi: 10.5858/arpa.2013-0328-RA

Reason: Wrong concept

1. Sipes C, Hunter K, McGonigle D, West K, Hill T, Hebda T. The health information technology competencies tool: Does it translate for nursing informatics in the United States? Comput Inform Nurs. 2017;35(12):609-614. doi: 10.1097/CIN.0000000000000408

Reason: Wrong outcome

1. Skiba DJ. Informatics competencies for nurses revisited. Nurs Educ Perspect. 2016;37(6):365-367. doi: 10.1097/01.NEP.0000000000000092

Reason: Wrong outcome

1. Skiba DJ. Students, technology, and teaching: Findings from the 2016 ECAR report. Nurs Educ Perspect. 2017b;38(1):51-52. doi: 10.1097/01.NEP.0000000000000117

Reason: Wrong outcome

1. Skiba DJ. Nursing informatics education: From automation to connected care. In: NI 2016, Switzerland. Studies in Health Technology & Informatics. 2017;232:9–19. doi: 10.3233/978-1-61499-738-2-9

Reason: Wrong study design

1. Skiba DJ. Nursing informatics education: From automation to connected care. Stud Health Technol Inform. 2017;232:9-19.

Reason: Duplicate

1. Strahan B. Self-Assessment of Nursing Informatics Competencies for Pre-licensure Nursing Students. J Inform Nurs. 2017;2(4):14-18.

Reason: Wrong outcome

1. Strudwick G, Hubert S, Gehrs M. Developing nursing informatics competencies for future nursing leaders: An overview of an internship and learning plan. Stud Health Technol Inform. 2018;250:67.

Reason: Wrong population

1. Strudwick G, Nagle L, Kennedy MA, White P, Lo B, Currie L. Supporting the development of informatics competencies among senior nurse leaders in Canada. Stud Health Technol Inform. 2021;284:189-190. doi: 10.3233/SHTI210701

Reason: Wrong population

1. Strudwick G, Nagle LM, Morgan A, Kennedy MA, Currie LM, Lo B, White P. Adapting and validating informatics competencies for senior nurse leaders in the Canadian context: Results of a Delphi study. Int J Med Inform. 2019;129:211-218. doi: 10.1016/j.ijmedinf.2019.06.012

Reason: Wrong population

1. Talcott K, O’donnell JM, Burns HK. Technology and the Nurse Educator: Are You ELITE? Nurse Educator. 2013;38(3):126-131.

Reason: Wrong outcome

1. Tanaka J, Kuroda H, Igawa N, Sakurai T, Ohnishi M. Perceived eHealth literacy and learning experiences among Japanese undergraduate nursing students: A cross-sectional study. CIN: Computers, Informatics, Nursing. 2020;38(4):198-203. doi: 10.1097/CIN.0000000000000611

Reason: Wrong outcome

1. Tarihoran DE, Anggraini D, Juliani E, Ressa R, Fardan I. Indonesian student nurses’ e-Health literacy skills. Stud Health Technolog. 2021. doi: [10.3233/SHTI210767](https://doi.org/10.3233/SHTI210767).

Reason: Wrong outcome

1. Tellez M. Nursing informatics education past, present, and future. CIN: Computers, Informatics, Nursing. 2012;30(5):229-233.

Reason: Wrong outcome

1. Terry J, Davies A, Williams C, Tait S, Condon L. Improving the digital literacy competence of nursing and midwifery students: A qualitative study of the experiences of NICE student champions. Nurse Education in Practice. 2019;34:192-198. doi: 10.1016/j.nepr.2018.11.016

Reason: Wrong outcome

1. Thapa S, Nielsen JB, Aldahmash AM, Qadri FR, Leppin A. Willingness to Use Digital Health Tools in Patient Care Among Health Care Professionals and Students at a University Hospital in Saudi Arabia: Quantitative Cross-sectional Survey. JMIR Medical Education. 2021;7(1):e18590. doi: 10.2196/18590

Reason: Wrong outcome

1. Theron M, Redmond A, Borycki EM. Baccalaureate Nursing Students’ Abilities in Critically Identifying and Evaluating the Quality of Online Health Information. Stud Health Technol Inform. 2017;234:321-327.

Reason: Wrong outcome

1. Theron MJ, Astle B, Dixon D, Redmond A. Beyond checklists: A nursing informatics education strategy for undergraduate nursing students appraising health information on social networking sites (SNS). Quality Advancement in Nursing Education. 2019;5(1):6. doi:10.17483/2368-6669.1174

Reason: Wrong concept

1. Thye J, Shaw T, Hüsers J, Esdar M, Ball M, Babitsch B, Hübner U. What are inter-professional eHealth competencies? Stud Health Technol Inform. 2018;253:201-205. doi: 10.3233/978-1-61499-896-9-201

Reason: Wrong population

1. Ting J, Garnett A, Donelle L. Nursing education and training on electronic health record systems: An integrative review. *Nurse Educ Pract*. 2021;55(103168):103168. doi:10.1016/j.nepr.2021.103168

Reason: Review

1. Tibes CM, Dias JD, Marcondes Westin U, Domingues AN, Zem-Mascarenhas SH, Martinez Évora YD. Development of digital educational resources for nursing education. J Nurs UFPE / Rev Enferm UFPE. 2023;17(3):1326-1334. doi: 10.5205/reuol.10263-91568-1-RV.1103sup201702

Reason: Wrong concept

1. Tissera S, Silva N. Self-reported eHealth literacy among undergraduate nursing students in selected districts of Sri Lanka. Stud Health Technol Inform. 2017;245:1339.

Reason: Wrong concept

1. Topaz M, Radhakrishnan K, Masterson R, Bowles KH. Putting evidence to work: Using standardized terminologies to incorporate clinical practice guidelines within homecare electronic health records. Online J Nurs Inform. 2012;16(2):23–27.

Reason: Wrong concept

1. Topaz M, Ronquillo C, Pruinelli L, Ramos R, Peltonen LM, Siirala E, et al. Central trends in nursing informatics: Students’ reflections from international congress on nursing informatics 2014 (Taipei, Taiwan). CIN: Comput Inform Nurs. 2015;33(3):85–89. doi: 10.1097/CIN.0000000000000139

Reason: Wrong concept

1. Troncoso EL, Breads J. Best of both worlds: Digital health and nursing together for healthier communities. International Nursing Review. 2021;68(4):504-511. doi: 10.1111/inr.12685

Reason: Wrong outcome

1. Tubaishat A, Habiballah L. eHealth literacy among undergraduate nursing students. Nurse Education Today. 2016;42:47-52. doi: 10.1016/j.nedt.2016.04.003

Reason: Wrong outcome

1. Tupper SR, Alexander D. Leading from the future: The nursing informatics executive. CIN: Comput Inform Nurs. 2012;30(3):123–125.

Reason: Wrong concept

1. Tyson RL, Brammer S, McIntosh D. Telehealth in Psychiatric Nursing Education: Lessons From the Field. Journal of the American Psychiatric Nurses Association. 2019;25(4):266-271. doi: 10.1177/1078390318807967

Reason: Wrong outcome

1. Tyler DD. A day in the life of a nurse informaticist. J Inform Nurs. 2017;2(1):24–26.

Reason: Wrong concept

1. Tyler DD. A day in the life of a nurse informaticist: Organizational change. J Inform Nurs. 2019;4(2):18–20.

Reason: Wrong concept

1. Tyler DD. A day in the life of a nurse informaticist: Understanding and influencing policy. J Inform Nurs. 2019;4(3):11–13.

Reason: Wrong concept

1. Tzitzolaki T, Tsiligiri M, Kostouda F. The use of information and communications technology from the educational staff of the nursing and physiotherapy departments of the Alexander Technological Educational Institution of Thessaloniki, Greece. Int J Caring Sci. 2014;7(1):203-210.

Reason: Wrong population

1. van Houwelingen CTM, Barakat A, Best R, Boot WR, Charness N, Kort HSM. Dutch nurses’ willingness to use home telehealth: Implications for practice and education. J Gerontol Nurs. 2015;41(4):47-56. doi: 10.3928/00989134-20141203-01

Reason: Wrong population

1. van Houwelingen CTM, Moerman AH, Ettema RGA, Kort HSM, ten Cate O. Competencies required for nursing telehealth activities: A Delphi-study. Nurse Educ Today. 2016;39:50-62. doi: 10.1016/j.nedt.2015.12.025

Reason: Wrong population

1. van Houwelingen CTM, Ettema RGA, Kort HSM, Ten Cate O. Hospital nurses’ self-reported confidence in their telehealth competencies. J Contin Educ Nurs. 2019;50(1):26-34. doi: [10.3928/00220124-20190102-07](https://doi.org/10.3928/00220124-20190102-07)

Reason: Wrong population

1. van Houwelingen CTM, Ettema RGA, Kort HSM, Ten Cate O. Internet-generation nursing students’ view of technology-based health care. J Nurs Educ. 2017;56(12):717–724. doi: [10.3928/01484834-20171120-03](https://doi.org/10.3928/01484834-20171120-03)

Reason: Wrong concept

1. van Houwelingen T, Ettema RGA, Bleijenberg N, van Os-Medendorp H, Kort HSM, ten Cate O. Educational intervention to increase nurses’ knowledge, self-efficacy and usage of telehealth: A multi-setting pretest-posttest study. Nurse Educ Pract. 2021;51:N.PAG-N.PAG. doi: 10.1016/j.nepr.2020.102924

Reason: Wrong population

1. Verma MP, Gupta S. Competency in informatics for nursing professional in India: Imbibing the tech-culture among nursing professionals. Int J Nurs Educ. 2019;11(1):67-73. doi: 10.5958/0974-9357.2019.00015.1

Reason: Wrong population

1. Viste J. NACNS white paper on telehealth competency for the clinical nurse specialist: Gap analysis and recommendations. Clin Nurse Spec. 2022;36(1):E1-E7. doi: 10.1097/NUR.0000000000000650

Reason: Wrong population

1. von Gerich H, Moen H, Block LJ, et al. Artificial Intelligence -based technologies in nursing: A scoping literature review of the evidence. *Int J Nurs Stud*. 2022;127(104153):104153. doi:10.1016/j.ijnurstu.2021.104153

Reason: Review

1. Wang J, Gephart SM, Mallow J, Bakken S. Models of collaboration and dissemination for nursing informatics innovations in the 21st century. Nursing Outlook. 2019;67(4):419-432. doi: 10.1016/j.outlook.2019.02.003

Reason: Wrong outcome

1. Wang Q-L, Liu L-L, Liu C-R, Zhu Q-S, Ren Z-Y, Gang T-T, et al. “Internet+” comprehensive nursing training course in the post-epidemic era-an exploration of the mixed teaching mode: A randomized trial. Front Med. 2023;10(101648047):1152732. doi: 10.3389/fmed.2023.1152732

Reason: Wrong concept

1. Webb R, Maisano P. A case for the nursing informatics Executive. The Oklahoma Nurse. 2017;62(1):7–14.

Reason: Wrong concept

1. Weiner E, Weaver C, Kennedy R, Marin H, Brennan PF. A Proposed Model for Advancing the Science of Nursing Informatics and its Value Proposition for Clinical Practice, Nursing Education and Research. Stud Health Technol Inform. 2013;192:1235-1235.

Reason: Wrong outcome

1. Wilson RL. The right way for nurses to prescribe, administer and critique digital therapies. Contemporary Nurse: A Journal for the Australian Nursing Profession. 2018;54(4/5):543-545. doi: 10.1080/10376178.2018.1507679

Reason: Wrong outcome

1. Wu Y, Wang Y, Ji M. Competencies related to informatics and information management for practicing nurses in select countries in Asia. Stud Health Technol Inform. 2017;232:86-96.

Reason: Wrong population

1. Wynn S. Preparing Today’s Nursing Students for Tomorrow’s Career. Issues in Mental Health Nursing. 2016;37(4):245-248. doi: 10.3109/01612840.2015.1130761

Reason: Wrong outcome

1. Yang L, Cui D, Zhu X, Zhao Q, Xiao N, Shen X. Perspectives from nurse managers on informatics competencies. Sci World J. 2014;391714:391714. doi: 10.1155/2014/391714

Reason: Wrong population

1. Yao X, Zhou Y, Wang Y, Li Z. Cross-disciplinary training of nursing informatics and nursing engineering at the postgraduate level: A feasibility analysis based on the qualitative method. Nurse Education Today. 2023;121. doi: 10.1016/j.nedt.2023.105708

Reason: Wrong outcome

1. Yen P-Y, Phillips A, Kennedy MK, Collins S. Nursing informatics competency assessment for the nurse leader: Instrument refinement, validation, and psychometric analysis. J Nurs Adm. 2017;47(5):271-277. doi: 10.1097/NNA.0000000000000478

Reason: Wrong population

1. Ying Wu, Yanling Wang, Meihua Ji. Competencies related to informatics and information management for practicing nurses in select countries in Asia. Stud Health Technol Inform. 2017;232:86-96. doi: 10.3233/978-1-61499-738-2-86

Reason: Wrong population

1. Yoon S, Shaffer JA, Bakken S. Refining a self-assessment of informatics competency scale using Mokken scaling analysis. J Interprof Care. 2015;29(6):579–586. doi: 10.3109/13561820.2015.1049340

Reason: Wrong concept

1. Yukie Majima, Seiko Masuda, Takeshi Matsuda. Development of augmented reality in learning for nursing skills...MEDINFO 2019, the 17th World Congress on Medical and Health Informatics, August 25-30, 2019, Lyon, France. Stud Health Technol Inform. 2019;264:1720–1721. doi: 10.3233/SHTI190614

Reason: Wrong concept.

1. Yu WW, Cheng C-Y, Lin CC, Wang J. Fostering nursing students’ informatics competencies via a web-based information literacy course. J Curric Teach. 2013;2(2):11-21.

Reason: Wrong population

1. Yu-tong T, Yan Z, Zhen L, Bing X, Qing-yun C. Telehealth readiness and its influencing factors among Chinese clinical nurses: A cross-sectional study. Nurse Educ Pract. 2022;58:N.PAG-N.PAG. doi: 10.1016/j.nepr.2021.103278

Reason: Wrong population
