# Supplementary material for: Digital Health Education and Training for Undergraduate and Graduate Nursing Students: Scoping Review
Source: JMIR Nurs. 2024 Jul 17;7:e58170. doi: 10.2196/58170 (PMC11292154; doi:10.2196/58170)
Supplement: Multimedia Appendix 3 [file nursing_v7i1e58170_app3.docx]

**Table 1: DH & NI interventions at the undergraduate level (n=23)**

| **Author/Year** | **Method/Pop** | **Instructional strategy used/Topic** | **Outcomes measured or explored** | **Evaluation/assessment strategies** | **Key Findings** |
| --- | --- | --- | --- | --- | --- |
| **Telehealth** |  |  |  |  |  |
| 1. Moore et al. 2023—USA*   Telehealth Simulation: Effect on Nurse Learner Knowledge, Confidence, and Attitudes. | Pilot Study: Pretest-posttest, nonexperimental design.  Senior -level Online BSN students (n=66) enrolled in an NI course  Online NP students (n=14) | A telehealth platform with a web-based clinician dashboard and a point-of-care mobile application that paired to telehealth peripherals. Three simulations lasting approximately 90 minutes. Didactic component (e-book, video) delivered via remotely and asynchronously, Pre-briefing sessions.  Topic: How to conduct telehealth visit, use of telehealth technology.  Framework: Rutledge et al (2017) multi-modal approach, Kolb’s experiential learning theory. | Knowledge, confidence, & attitudes. | TeleOSCE, pre and post intervention survey | Survey scores significantly increased from pre-to post the intervention. Students valued the learning experience. |
| 1. Eckhoff, Diaz et al., 2022—USA*   Using Simulation to Teach Intraprofessional Telehealth Communication. | Descriptive quantitative design  60 students (54 prelicensure, 6 NP) | Online module about telehealth and IP competencies, simulation with videoconferencing i.e., telehealth robot, briefing and debriefing.  Topics: Intraprofessional competencies, telehealth etiquette, professionalism, peripherals, technologies, documentation, billing, collaboration, & history taking.  Framework: AACN (2021), NONPF  NLN Jeffries Simulation Theory, Telehealth education model  PEARLS method for debriefing. | Knowledge Usability of the telehealth robot | Both groups completed a pre and post knowledge test. Post simulation, both groups completed the system usability scale, simulation-based education effectiveness survey, ICCAS survey. | Students perceived the experience as effective. High perceived system usability.  Increase in students’ perceived ability with intra-professional communication. |
| 1. Brownie et al., 2022—New Zealand*   Evaluating an undergraduate nursing student telehealth placement for community-dwelling frail older people during the COVID-19 pandemic. | Case study evaluation (mixed methods—questionnaire & Interviews)  BSN Nursing students (n=19)  Tutors (n=5) | Telehealth clinical placement experience.  Framework: Not specified. | Student and tutor experiences of telehealth placement. | Pre- and post-placement questionnaires, reflective diaries during the placement, post-placement interviews and focus groups. | Themes/lessons learned: tackling COVID-19, implementation requirements, nursing competencies, provider relationships, and community insights. |
| 1. Ochs et al., 2022—USA*   Telehealth in nursing education: Navigating the new normal. | Evaluation study.  BSN (n=400) | A telehealth simulation scenario over an online platform, standardized patients, using scenarios to teach telehealth.  Framework: Not specified. | Feasibility of telehealth to replace clinical experiences, measurement of clinical competency during telehealth simulation. | Formative and summative assessments using OSCE and grading using the C-CEI tool, debriefing, and feedback sessions. | 67% students earned a score of 90% or higher. Telehealth is appropriate and should be incorporated as part of the curriculum, not as a ‘backup.’ |
| 1. Victor et al., 2021—USA*   Telehealth Simulation: An Online Clinical Experience | Descriptive study  BSN students (n=58)  Clinical instructors (n=5)  Senior BSN students acting as SP (n=17) | A telehealth simulation scenario over 1 hr., as an online clinical experience, facilitated within the Google Hangout platform, with standardized patients, pre-post briefing.  Framework: INACSL standards  PEARLS Principles guided debriefing session | Suitability of the telehealth simulation for achieving course outcomes | A checklist for formative evaluation—no scores, a debriefing session, post-section reflections | Positive feedback from students and tutors. Potential for scaling up and testing through different platforms. |
| 1. Powers et al. 2020—USA   Baccalaureate nursing students' experiences with multi-patient,  standardized patient simulations using telehealth to collaborate. | A phenomenological qualitative study  Final semester BSN students (n=27) | Two telehealth enhanced simulation activities using telehealth technology (Facetime application via iPad) lasting 5-6 hours as part of a course; classroom orientation, board games. Pre- simulation activity consisted of creation of concept maps, pre-briefing session. Simulation learning objectives provided.  Framework: QSEN competencies. INACSL Standards of Best Practice: Simulation ^SM^ used for creating the simulation. PEARLS tool used for debriefing. | Experiences and perceptions. | Focus group interviews. | Five themes: Anxiety due to lack of experience, Improved clinical reasoning, Real-world practice, How to communicate effectively, and Application to clinical practice. |
| 1. Lister et al. 2018—USA   Telehealth and telenursing using simulation for pre-licensure USA students. | Descriptive study    BSN students (n=73) | Home care simulation as part of an existing course, using the telepresence robot with a two-way video conferencing technology, briefing, debriefing.  Framework: Not specified. | Confidence in communicating via video, opinion in value of video technology to improve healthcare, assessment skills. | Pre- and post-surveys (Shortened version of the SDS & SCLS). | Statistically significant improvement in the students' degree of confidence concerning the activities, tasks, and resources used in the simulation |
| 1. Molloy et al. 2016—USA   An Innovative Use of Telepresence Robots for Educating Healthcare Professional. | Descriptive quantitative design  53 students (n=48  BSN students; n=5 NP students) | Telepresence robot as a tool to introduce telehealth and to engage distance-based students in clinical simulation.  Framework: Not specified. | Feasibility and acceptability of the telepresence robot. | Post surveys. | High mean scores on the acceptability of using the robot. |
| 1. Thomas et al. 2023—USA*   Use of Simulated Telenursing with Standardized Patients to Enhance Prelicensure Nursing Education. | Quasi-experimental quantitative study (Conducted over 4 school terms)  BSN students (n=41- 48) | Develop a telenursing event modality, standardized patients, using Zoom videoconferencing, 4 case scenarios, pre-assignments, debriefing. Simulation learning objectives provided.  Framework: AACN Essentials. | Comfort and confidence in communication and patient interaction. Reinforce critical theory information. | Student performance on content-related examinations and debriefing. | Improvement on exam for students in telenursing intervention compared to standard curriculum. Increased comfort & confidence. |
| 1. Watkins et al. 2022—USA   Simulated Telenursing Encounters with Standardized Participant Feedback for Prelicensure Nursing Students. | Descriptive study  BSN students (n=32) | Telenursing simulation experience via video conferencing, standardized patients, pre briefing, self-reflection, and debriefing.  Framework:  NLN Jeffries Simulation Framework. | Perceptions regarding the design of the simulation experience, value of the experience, faculty facilitation of the experience. Communication, & interviewing skills. | Self-reflection, evaluation survey and debriefing. | Mean scores ranged between 2.00 – 4.8; recommendations for more information during pre-briefing, include other interprofessional students, uncooperative patients, and difficult conversations. |
| 1. Reierson et al. 2015—Norway   Nursing Students’ Perspectives on Telenursing  in Patient Care after Simulation. | Exploratory -Qualitative design - Focus group interviews.  BSN students (n=32) | Three telenursing scenarios (20 minutes each) using real-time video and audio technology; 5-hour session (2 hrs. theoretical background and three hrs. simulating telenursing scenarios); pre briefing and debriefing as part of the focus groups.  Framework: Not specified. | Experiences and reflections on telenursing experience. | Focus groups for debriefing | Five themes: learning a different nursing role, influence on nursing assessment and decision making, reflections on the quality of remote comforting and care, empowering the patient, and ethical and economic reflections. |
| **EHRs** |  |  |  |  |  |
| 1. Mollart et al., 2023—Australia   The impact of using an academic  electronic medical record program on  first-year nursing students’ confidence and skills in using E-documentation:  a quasi- experimental study. | Quasi-experimental design  BSN students (1^st^ year, n = 105). | Evaluating the AeMR integration in first clinical course, AeMR accessible via mobile devices throughout the term, video on how to use the AeMR, two tutorials with a case scenario. The AeMR developed by a member of the University Learning Design and Teaching Innovation Department.  Framework: Not specified. | Confidence, knowledge, and skill in e-documentation. | Pre and posttest surveys (quantitative and qualitative data). | Significant increase in confidence and knowledge in documenting.  Themes: preparation for practice; more exposure increases confidence; and we can’t forget the patient. |
| 1. Hong et al., 2022—South Korea   Simulation Education Incorporating Academic  Electronic Medical Records for Undergraduate  Nursing Students: A Pilot Study. | Pilot Study—Mixed methods design (one group post-test only & focus group)  BSN students (3^rd^ & 4^th^ yr., n = 76) | Three simulated case scenarios, incorporating a cloud based EMR application, pre-briefing with opportunities to practice, simulation, recording nursing care in the AEMR.  Framework: Not specified. | Usability of the AEMR system, self-efficacy of AEMR utilization, Perspectives on the learning experience | Post survey and focus group interviews | The average mean scores for the AEMR system’s usability and participants’ self-efficacy for AEMR utilization were 5.36 of 7 and 3.96 of 5, respectively.  Themes: Immersion in the simulation education, Training to find and prioritize patients’ information, Confidence in EMR use in practice, Necessity of pre-education and time for familiarizing oneself of the AEMR, Barriers to documenting a nursing process in the AEMR, & Need for AEMR settings considering students’ level (p. 383). |
| 1. Kleib et al., 2021—Canada   Academic Electronic Health Records in Undergraduate Nursing Education: Mixed Methods Pilot Study. | Pilot study—Mixed methods (one-group pretest-post-test, surveys, & focus groups)  Senior-level BSN students (n=13).  Instructors (n=3) | Evaluating a DocuCare simulated EHR (DocuCare), orientation, a training webinar & written guide, 4 case scenarios over one month.  Framework: NI entry-to-practice competencies for RNs; Experiential learning. | Self-reported NI knowledge and attitudes toward the EHR, accuracy of e-documentation, satisfaction with DocuCare, and students’ and educators’ experiences. | One-group pretest-post-test and focus group interviews. | A statistically significant difference in the mean score of knowledge. No statistically significant difference in the mean scores of attitudes. Documentation scores varied. Faculty and students were highly satisfied and recommended integration. Themes: Current challenges related to Documentation, Motivating factors for embracing computerized charting as an educational tool, Educators’, and students’ experiences with DocuCare, Educators’ and students’ suggestions for improving DocuCare, & Recommendations for integrating DocuCare in undergraduate curricula. |
| 1. Repsha et al., 2020—USA   Use of a Simulated Electronic Health Record to Support  Nursing Student Informatics Knowledge and Skills. | Pilot study—cross-sectional survey  64 BSN students in senior-level simulation laboratory courses (n=37) | Simulated EHR (third-party), preparation assignment, actual assignment.  Framework: Not specified | NI knowledge and skills | Pre and post intervention surveys using 18-items SANICS | A statistically significant difference in NI competency scores. |
| 1. Elliott et al., 2018—UK   Teaching student nurses how to use electronic patient records through simulation: A  case study. | Case study  BSN students (n=296) | A simulation activity about the EPR, developing scenarios, creating a Firefox web-based system & an app for students to access and view patients, cases, and the simulated ward. iPads provided to students, pilot testing of the app, roll out and evaluation.  Framework: Not specified. | Student experiences of using the simulated EPR app. | Questionnaire | Enhanced ability to use the EPR, positive views of the simulation experience. |
| 1. Choi et al., 2016—Korea   Nursing Students’ Satisfaction with Mobile  Academic Electronic Medical Records for  Undergraduate Clinical Practicum. | Quasi-experimental study.    3^rd^ yr. BSN students (n=58) | Clinical practicum placement. Experimental group had Mobile AEMR application on iPad.  Control group used PC-based EMR system.  Framework: Not specified. | Satisfaction and usability of the mobile application for intervention and documentation. | Pre- and post-tests. | The experimental group showed generally positive satisfaction and intentions to use it.  Students who used the AEMR application were more satisfied with understanding the diagnostic and laboratory tests, nursing intervention and documentation. |
| 1. Choi et al., 2015—Korea   Usability of Academic Electronic Medical Record  Application for Nursing Students’ Clinical Practicum | Pilot study  3^rd^ yr. BSN students (n=5) | Evaluation of an AEMR using 15 case scenarios, an iPad; introductory overview of the application was provided.  Framework: Not specified. | Usability and functionality of the AEMR for documentation. | “Think aloud” method used to assess students’ ability to interact with the technology (Comments recorded and transcribed). | Minor usability issues identified; application enhanced students’ familiarity with electronic records for documentation. |
| 1. Titzer et al., 2015—USA   Interprofessional education: Lessons learned from conducting an electronic health record assignment | A mixed methods study.  Interprofessional (nursing, health informatics, & radiology technology) (n=56) | The assignment required students to collaboratively create a patient record based on a case study and instructions on how to document in the AEHR.  Framework: IP core competencies, QSEN. | Perceived informatics competency | ICA tool (knowledge, skills, and access).  Post assignment survey. | Assignment objectives were met. Improvements noted in knowledge, skills, and access with a statistically significant findings on some items of the ICA tool. Post survey showed students found the experience useful in providing real-life experience. |
| 1. Warboys et al., 2014—USA   Electronic Medical Records in Clinical Teaching | Nonexperimental, correlational design  BSN students from different cohorts(n=220) | An open-source (OpenEMR) software accessed by students during the 15-week semester, 50-minutes of training sessions and a PDF file provided online for further direction.  Framework: Not specified. | Perception of EMR as a learning tool, level of EMR use needed for positive perception of the EMR and experience on EMR. | EMR use Survey. | Students who  used the EMR 5 or more times had more positive perceptions about the EMR learning experience. |
| 1. Kowitlawakul et al., 2013—Singapore   Development of the electronic health records for nursing education  (EHRNE) software program | Pilot study-Qualitative evaluation using focus groups.  Students in the first three years of the program learning in simulation/lab (n=9) | Developing & evaluating the EHRNE (software application). It can be downloaded to student computer, focused on documentation, case studies, participants used EHRNE for 1.5 hrs., orientation & instructions on how to use the program.  Framework: Not specified | Students’ experiences with EHRNE. | Focus group interviews | Four themes: functionality, data management, timing and complexity, and accessibility.  Educational sessions for both students and faculty outlining the software's purpose, advantages, and limitations are needed before integration. |
| **NI** |  |  |  |  |  |
| 1. Almarwani & Yacoub, 2023—KSA   Examining the Effect of an Educational Program on Nursing Students’ Informatics Competencies. | A one-group pretest-posttest design.  BSN students (n=83). | 2-day online NI educational program, each day had specific objectives, applied didactic sessions, videos, modules, and hands-on practice on using the EHR.  Topics: Key concepts in NI  Framework: 2022 ANA Scope and Standards of Practice for Nursing Informatics. | NI competency | 18-items SANICS before and after the intervention | A statistically significant improvement in NI competency. |
| 1. Kleib & Olson, 2015—Canada   Evaluation of an informatics educational intervention to enhance informatics competence among baccalaureate nursing students. | A three-group post-test only randomized controlled design.  BSN students (n=42) | Developing and evaluating an NI educational intervention. Two learning modules about HI & NI, topic outline for each module with various instructional activities, piloting the intervention, and then testing the efficacy of face-to-face lectures and vodcasts for delivering the content.  Framework: A blended objectivist-constructivist pedagogical framework. Beginner-level NI competencies by Staggers, Gassert & Curran, 2001) | Knowledge gain, attitudes toward the EHR, perceived confidence | A three-group post-test only design | The intervention had a large effect on knowledge gain, but no effect on confidence or attitudes. Vodcasting was equally effective to face-to-face methods for  delivering informatics content. |

**Legend:** BSN – Baccalaureate Nursing Education; NP- Nurse Practitioners; Yr. – Year; AeMR - Academic Electronic Medical Record; AEMR - Academic Electronic Medical Record; EHRNE - Electronic Heath Record for Nursing Education;  QSEN - Quality and Safety Education for Nurses; SANICS - Self-Assessment of Nursing; EPR - Electronic Patient Record; AEHR - Academic Electronic Health Record; ANA – American Nurses Association; NONPF - National Organization of Nurse Practitioner Faculties; AACN – American Association of Colleges of Nursing; NLN – National League of Nursing; PEARLS - Promoting Excellence and Reflective Learning through Simulation; C-CEI- Creighton Competency Evaluation Instrument; ICCAS - Collaborative Competency Attainment Survey; SSSCL - Student Satisfaction and Self-Confidence in Learning; TeleOSCE - Telemedicine Objective Structured Clinical Exam; IP – Intraprofessional; INACSL *-* International Nursing Association of Clinical Simulation and Learning; PEARLS - Promoting Excellence and Reflective Learning through Simulation; NI – Nursing Informatics; EHR – Electronic Health Record; EPR - Electronic Patient Record; SP- Standardized Patients; RN – Registered Nurses; OSCE- Objective Structured Clinical Exam; SDS- Simulation Design Scale; SCLS- Self-Confidence in Learning Scale; e- Electronic; EMR- Electronic Medical Record; ICA- Informatics Competency Assessment; EMR- Electronic Medical Record.

(*): COVID Publication

**Table 2: DH & NI interventions at the graduate education level (n=38)**

| **Author/Year** | **Design/Pop** | **Instructional strategy used/intervention/Content** | **Outcomes measured or explored** | **Evaluation/Assessment strategies** | **Results/Key Findings** |
| --- | --- | --- | --- | --- | --- |
| **Telehealth** |  |  |  |  |  |
| 1. LaManna et al., 2023—USA*   Applying Nurse Practitioner Student Reflections in Design of Telehealth Curricula. | Pilot study—mixed method (qualitative findings based on content analysis of student-generated reflections).  32 students enrolled in DNP Primary care NP program. | Simulated, synchronous telehealth encounter using a case scenario.  Online preparatory module & assessment quiz before the experience. Objectives of simulation provided.  Framework: AACN Essentials, NONPF, INACSL Best Practices for clinical simulation, The 4 Ps framework. | Reflections regarding 1) comfort with learning activities, 2) potential impact of the telehealth experience on future practice, 3), strategies applied in relationship building with patient and caregiver, & 4) similarities and differences in strategies used in relation building during face-to-face and telehealth encounter. | A guided reflection as a written assignment regarding self-performance after the experience. | Results provided insights into planning curricular integration of telehealth. |
| 1. Jones et al., 2023—USA*   Evaluating an intervention of telehealth education and simulation for advanced practice registered nurse students: A single group comparison study. | Quasi-experimental: A single group comparison study.  APRN students (n=68) | An online telehealth instruction (videos, readings, web links, live Q/A) & simulation with SP, over 2 weeks. Content & objectives (Rutledge, 2021).  Framework: The 2016 INACSL Standards for Best Practice in Simulation, NONPF. | Beliefs and confidence in performing telehealth. | Pre-test and post-test. Standardized rubrics, Faculty feedback, & debriefing sessions. | Students' beliefs did not significantly change between the pre- and post-intervention, in which all areas were rated high pre-intervention.  Students reported an increase in their perception and confidence post-intervention. |
| 1. Baliko et al., 2023—USA*   Developing Family Nurse Practitioner Student Competencies: A Two-Part Mental Health-Focused Telehealth Education Activity. | Educational activity.  6 cohorts of NP students (n=135) | Two-parts simulation initiative: 1) Didactic modules including recorded lectures and pre-assigned readings, 2) Simulation encounter with a SP and debriefing.  Framework: AACN 2021: Competencies in Domain 8 (Informatics and Healthcare Technologies—8.1g, 8.3g, 8.4f); Domain 2 (patient-centred care); NP telehealth competencies. | Relevance of telehealth training, complexity of education, interest in learning more and incorporating telehealth in future practice, prior training experience with telehealth. | Post-education surveys and a focus group. | Students reported positive feedback and suggestions to improve the activity and supported its integration in the curriculum. |
| 1. French et al., 2023—USA   “Because of this rotation, this is what I want to do”: Implementation and evaluation of a telehealth opioid use disorder clinical placement for nurse practitioner students. | Program/training evaluation.    NP students (n = 57) | A telehealth clinic as a clinical rotation.  Framework: Not specified | Knowledge and attitudes about providing OUD care, preparedness, and acquisition of skills. | Pre-post-surveys and semi structured interviews. | Varied responses to the surveys (increased knowledge, preparedness, & acquisition of skills to treat OUD. Themes: the continuum of learning opportunities, the comfort providing OUD treatment after participants' clinical rotation, and relevance of a substance use disorder clinical rotation for all NP students. |
| 1. Chrostowski & Tietze, 2022—USA*   Using a Telemedicine Cart for an (OSCE) in Nurse Practitioner Education. | A course-based activity development and evaluation.  29 NP students (n=28) | Clinical examination simulation activity using a telemedicine cart for a virtual patient evaluation. Defined learning objectives for the activity, didactic content, live orientation to the telemedicine cart, and OSCE procedures.  Framework: INACSL (2017), Tietze Telehealth Framework. | Perceptions of the experience (knowledge, skills, and attitudes—KSA). | Grading of OSCE experience using a rubric, post OSCE written reflection, using a survey to collect quantitative and qualitative data | Overall, positive perceptions of the experience. Comparison of KSA between three cohorts didn’t yield statistically significant differences. |
| 1. Chike-Harris, Lauerer et al., 2022—USA*   Telehealth Professionalism Education for Advanced Practice Nursing Students. | Telehealth Activity.  APRN students (n=59) | A self-paced module about telehealth professionalism. An initial self-paced module with a pretest to assess prior knowledge, viewing 2 video-lectures, face-to-face discussion on campus.  Framework: Not specified. | Knowledge. | Pre and post-tests. | Posttest scores indicated improvement in knowledge gain. Students indicated interest in more education. |
| 1. Berrier & Hellier et al. 2022—USA*   Addressing telehealth education in a family nurse practitioner program through simulation-based learning. | Educational evaluation.    FNP students (n=15) | Simulation-based learning module about telehealth medical skills with defined objectives (comprised electronic slide presentation and tabletop exercises) used in pre-briefing, class discussion with faculty, SPs, and a simulation day with summative assessments.  Framework: Kolb’s experiential learning theory and the Transformation Learning Theory, INACSL (2016), Telehealth competencies with corresponding learning objectives,  PEARLS conceptual framework guided the debriefing. | Satisfaction with simulation learning experience, self-confidence for clinical application. | Evaluation rubrics, summative assessment, survey, group debriefing session. | Simulation-based learning experience was satisfying and contributed to confidence building in applying skills learned in the clinical setting. Telehealth education needs to be incorporated in NP training. |
| 1. VanGraafeiland et al. 2022—USA   A Mock Telephone Triage Experience for Nurse Practitioner Students. | A quasi-experimental design.  NP students (n=39) | Evaluating the feasibility of an after-hour telephone consultation. The Mock triage consisted of 4 scenarios, each 15 minutes, faculty role-played the patient role, and debriefing sessions.  Framework: Not specified. | Perceived confidence, competence, readiness to practice, and satisfaction with the experience. | Rubric used to evaluate students’ performance. Pre- post surveys. | Enhanced preparedness, confidence, and perceived ability to provide telephone consultations. |
| 1. Lattner et al., 2022—USA*   Creating an Asynchronous Telehealth Simulation for Advance Nursing Practice Students. | Telehealth activity.  Graduate level post licensure nursing students. | Asynchronous telehealth SBE, using a web-based teleconference tool as a learning strategy in an online advanced course. Pre-simulation activities (reading assignments, videos, and asynchronous pre-briefing), modules, discussion board activities (each topic had it owns learning objectives).  Framework: PEARLS tool was used for the debriefing process. INASCL Standards of Best Practice: Simulation Outcomes and Objectives. | Clinical decision- making and collaboration with telehealth. | Debriefing sessions synchronously via a web-based teleconferencing tool. | Preliminary takeaways showed that students valued the learning experience. Using LMS proved useful and cost-effective. |
| 1. Emerson et al 2021—USA   Telehealth Simulation With Graduate Nurse  Practitioner Students | Pilot study.  NP students (n=11) | Telehealth simulation via video conference technology, online training modules, in-person training simulation day using scenarios and role playing, actual telehealth simulation with SPs.  Framework: NONPF competencies for telehealth. INASCL. | Readiness and confidence with simulation. Satisfaction with the simulation experience materials and activities | Debriefing sessions, pre and post surveys. | Statistically significant results between pre-post simulation confidence and readiness scores.  Satisfaction with the simulation experience scores ranged between 3.89 - 4.57. |
| 1. Chike-Harris, Garber, et al., 2021—USA*   Integration of Telehealth Policy Education into an Existing Advanced Practiced Nurse Practitioner Policy Course. | Telehealth Activity.  APRN students—module offered to three cohorts (N=263) | A self-paced module about telehealth policies, laws and regulations, pre-recorded lectures, & interactive live lecture with guest speakers via teleconferencing.  Framework: NONPF competencies for telehealth, AACN. | Telehealth. | Pre and post-tests. | An average of 40.4% increase in knowledge between pre-post-tests. Positive student feedback. |
| 1. Ball et al., 2021—USA   A Pediatric Telehealth Simulation for Prelicensure and Advanced Practice Nursing Students | Descriptive  NP student (n=16) as healthcare providers, Prelicensure student (n= ~18) acting as nurses | Virtual simulation experiences using a 4-foot-tall mobile robot with an iPad attached to the top, controlled remotely. Role-play of patient/family and provider roles. NPs led debriefing session with prelicensure students.  Framework: Not specified | Providing care virtually | A rubric for determining assessment skills, critical thinking, communication, and leadership. | Students valued the experience as a tool to increase comfort with providing care virtually. |
| 1. LaManna et al. 2021—USA*   Nurse Practitioner Student Perceptions of a Pilot Simulated Gerontologic Telehealth Visit. | Pilot study (Mixed method)—Quantitative findings.  NP students (n=33) | Simulation using a Double 2™ telehealth robot with an iPad as the monitor, pre-briefing, and online learning module, standardized patients.  Framework: The NLN, Jeffries Simulation The­ory, Rut­ledge et al. (2017) multimodal framework for telehealth education, INACSL. | Perceptions of the telehealth visit. Simulation effectiveness, simulation realism, system usability and learnability. | Survey and debriefing. | Overall, students found the telehealth robot experience effective & realistic. Telehealth robot is usable. |
| 1. Arends et al. 2021—USA   Enhancing the nurse practitioner curriculum to improve telehealth competency. | Curriculum development and evaluation.  FNP students (n= 171) | Developed 22 telehealth provider competencies, competencies informed learning outcomes. Developed an evidence-based didactic curriculum with a number of courses delivered using different strategies (presentations, assignments, clinical practicum, and simulation experiences with videos for review, & debriefing sessions).  Topics: An overview of telehealth, camera considerations, use of equipment, troubleshooting, reimbursement, legislation, quality initiatives, enhancing outcomes, direct-to-consumer telehealth services, & future trends (p. 395).  Framework: Guidelines available to telehealth practitioners. | Knowledge and skill levels, confidence, abilities, overall learning experiences. | Pre and post-tests at the beginning and end of each practicum course. | Significant change in mean score of confidence, and ability between pre-post-tests. |
| 1. Love & Carrington, 2020—USA*   Introducing telehealth skills into the Doctor of Nursing Practice curriculum. | Pilot study.  DNP NP students (n=83) | Telehealth education provided through 6 online modules, then students interviewed a simulated patient and provided a collaborative report.  Topics:  Topics: 1) telehealth in practice and consultation, 2) rationale for telehealth practice, 3) enhancement of consultation and collaboration with providers using telehealth, 4) privacy and confidentiality issues related to telehealth, 5) equipment utilized and common issues for telehealth.  Framework: Guidelines by the American Telemedicine Association. | Assess modules and the learning experience. | Post pilot questionnaires for students and simulated patients (n = 12). | Out of 94% of the students completing the survey, 16% felt prepared to interview the patient and 17.5% felt prepared to lead an interprofessional collaborative meeting. Positive feedback from students and patients but more work is needed to address gaps in students’ readiness and technology. |
| 1. Cassiday et al., 2021—USA*   Exploring telehealth in the graduate curriculum. | QI project.  FNP students in two different courses (n=57) with of students from different levels (2^nd^ &3^rd^ years). | A two‐part evidence‐based curriculum consisting of a lecture and 2-hour simulation involving small groups run via Microsoft Teams©, debriefing, and documentation.  Topics:  What is telehealth and how to deliver it to the patient.  Framework: NONPF | Students’ views about the learning experience and impact of simulation on comfort level to use telehealth technology, learning needs. | Evaluation survey with close & open-ended questions after the online lecture and simulation. | Mean scores simulation effectiveness (4.9) and means score of opinions to continue simulation (4.8).  Two themes: realism and engagement. There is a need for telehealth curriculum. |
| 1. Wesemann et al. 2021—USA*   Clinical simulation to evaluate students’ Intraprofessional telehealth skills between multiple university campuses. | Pilot study  NP students (n= 6) | Telehealth skills and competence: Telehealth simulation conducted using the Zoom video conferencing platform. Developed learning objectives that focused on the using interprofessional approach in telehealth.  Framework: RAM, INACSL standard. | Perceptions, interprofessional collaboration using technologies. Telehealth competencies (knowledge, skills, clinical abilities). | Post simulation survey, group debriefing, students completed a self-reflection with open-ended questions about their performance, experience, and telehealth strategies. | 83% of students indicated learning from the activity, thought it was believable, and recommended integration.  Two themes: Need to improve telehealth skills, importance of IP collaboration. |
| 1. Knight & Prettyman, 2020—USA   Rural Telehealth Team Education for Baccalaureate and Nurse Practitioner Students. | Pilot study.  NP students acting as providers (n=19)  BSN students acting as RNs (n=10) | Telehealth skills and competence: A telehealth consultation simulation model, rural setting, standardized patients, pre-briefing, followed by the simulation encounter.  Framework: TAM. | Proficiency in telehealth consultation, IP communication, comfort in using technology, role development. | Post simulation debriefing, survey. | Students found the simulation experience useful. |
| 1. Gibson et al. 2020—USA   Reinforcing Telehealth Competence Through Nurse Practitioner Student Clinical Experiences. | A needs Assessment.  FNP students in the final clinical practicum course.  (n=22)  Clinical preceptors (n=19) | Telehealth skills and competency: Partnership with clinical organizations to identify possible clinical placement opportunities. These were live video patient visits from remote and hub locations. Students engaged in a telehealth clinic rotation in a hub that provide the best learning environment. Clinical preceptors supervised students.  Framework: NONPF, telehealth competencies. | Competence in providing telehealth. | Telehealth evaluation tool completed preceptors and students. | Students met seven of the eight competency criteria, and the preceptors met eight of the 13 evaluation criteria.  Preceptor themes: professionalism, engagement, good communication, asking appropriate questions. Student themes: use of the interprofessional team, a variety of learning experiences, knowledgeable, professional, maintained confidentiality, and welcomed questions. |
| 1. Robinson-Reilly et al. 2020—Australia   Adding telehealth simulation into NP programs. | Pilot study.  NP students (n=20) geographically dispersed. | Telehealth skills and competency: A 5-day on campus Telehealth simulation via VSLV using diverse case scenarios. Pre-briefing, SPs, experience is within an existing health assessment course, experience augmented clinical application.  Framework: Aligned with curriculum philosophy (problem-based learning); practice standards | Learner experiences, engagement, and satisfaction. | Debriefing, course evaluation, peer observation and feedback. | Students found the experience useful, learning from peers, learner satisfaction and engagement improved. |
| 1. Phillips et al. 2020—USA   Assessing the impact of telehealth OSCES in graduate nursing education. | Education evaluation.  NP students (n=28) | Telehealth OSCE Simulation to improve clinical competency.  Didactic telehealth content taught in an advanced health assessment course, followed by telehealth OSCE in a clinical course, OSCEs were delivered using a free video conferencing system.  Framework: NONPF. | General understanding of telehealth; Experience of learning. | Pre and post survey. | 13 out of the 15 telehealth perception items revealed statistically significant differences between pre-post surveys. Four themes: 1) usefulness of telehealth, 2) benefit in role preparation, 3) incorporating technology into experiential learning, and 4) perceptions of the learning experience. |
| 1. Chike-Harris et al., 2020—USA   Graduate Nursing Telehealth Education: Assessment of a  One-Day Immersion Approach. | Pilot test.  DNP students (n=38) | One-day telehealth immersion event (lectures on various telehealth topics, hands-on practice on telehealth cart and other mobile equipment for familiarity, a mock tele-health visit).  Topics: Introduction to Telehealth, use and development of mobile health tools, policies, laws, and regulations regarding APRN telehealth practice, overview of the AVizia 310 telehealth cart and other mHealth equipment.  Framework: NONPF. | Knowledge of telehealth in practice and comfort level. | Pre and post-tests, program evaluation. | Average student posttest scores increased by 8% compared to pretest scores (59% to 64%). Program evaluation revealed students had limited knowledge prior to simulation. Participation of students via WebEx and in person may have impacted results. |
| 1. Quinlin et al., 2020—USA   Development and implementation of an e-visit OSCE to evaluate student ability to provide care by telehealth. | Educational evaluation.  Three cohorts of FNP students (n=103) | Telehealth skills—e-visit:  Development and implementation of an OSCE delivered via web conferencing. Students completed a 15-minute e-visit OSCE assessing and managing the care of a SP.  Framework: NONPF competencies for telehealth. | Abilities to deliver care via telehealth. | Faculty and student feedback and evaluation, grading rubrics, & structured group debrief. | Students from all cohorts valued the learning experience, increased confidence in decision-making and readiness to provide care. |
| 1. List et al., 2019—USA   Improving Telehealth Knowledge in Nurse Practitioner Training for Rural and Underserved Populations. | A QI project to curricular redesign.  FNP students (n=24 pre; n=22 post) | Conducted a needs assessment to identify where telehealth content can be integrated in an existing course. Telehealth learning outcomes, one-hour lecture by an expert presentation which included video conferencing, real-time telemonitoring.  Topics: What is telehealth, defining telehealth, how telehealth may improve patient outcomes, examples of telehealth use, benefits of telehealth.  Framework: PDSA cycle of change, Bloom’s taxonomy, Bandura’s self-efficacy theory. | Confidence in telehealth knowledge. | Pre and post intervention surveys. | Improved telehealth knowledge. Change in telehealth knowledge from pre-to-post intervention was statistically significant.  Provided information that faculty can use in considering more permanent changes to the curriculum. |
| 1. Smith et al., 2018—USA   Using simulation to teach telehealth competencies | Piloting a simulation education activity.  APRN students (Two simulations: Each simulation included 15 groups with two students in each). | Telehealth knowledge and skills:  Simulated telehealth experience, pre-simulation online didactic module on basics of telehealth, reading assignments, pre-briefing, OSCE experience face-to-face with a simulated patient.  Topics: A review of telehealth competencies, equipment, benefits, potential barriers, and re­imbursement issues (p. 625).  Framework: INACSL (2016), Telehealth competencies with corresponding learning objectives,  PEARLS conceptual framework guided the debriefing session | Benefits of simulation to expose students to Telehealth. | Submission of a SOAP Note after the experience and overall experience evaluation. | 1. It would be beneficial to include a pre–posttest to assess learning outcomes and telehealth knowledge, skills, and attitudes. 2. Students reported that the telehealth simulation gave them an opportunity to practice a skill to which they had limited exposure in the clinical setting. 3. Introduction of telehealth earlier in the program creates an opportunity for faculty to design more experiences related to telehealth. |
| 1. Merritt et al., 2018—USA   Using a Web-Based e-Visit Simulation to Educate Nurse Practitioner Students | Pilot Study  Senior-level NP students (n=26) | Developed and implemented a Web-based, e-Visit simulation experience using a case scenario, SPs, online module.  Topic: Module included a description of telehealth, e-visits, role of these emerging technologies in the future of healthcare (p. 306).  Framework: Ericsson’s and Smith’s expertise theory (1991). | Satisfaction with learning experience. | Post survey. | Students reported the experienced enhanced confidence in providing care to actual patients, 97% believed the simulation resembled real-world patients. |
| 1. Erickson et al., 2015—USA   Integrating Telehealth Into the Graduate Nursing Curriculum | Educational evaluation.  NP students (n=72) | One-hour classroom presentation by a telehealth expert and a demonstration via videoconferencing equipment with a live connection to the hospital information technology laboratory, 4-hour clinical rotation using telehealth with a preceptor following the lecture.  Topics: Telehealth—barriers and challenges with providing care via telehealth, licensure requirements for NP as per patient location, providers, services, and patients sites eligible for reimbursement (p. e2)  Framework: Not specified | Overall knowledge, satisfaction, & interest in using telehealth. | Not specified. | Not specified. |
| 1. Rutledge et al. 2014—USA   Telehealth: Preparing Advanced Practice Nurses to Address Healthcare Needs in Rural and  Underserved Populations. | Educational enhancement  DNP APRN students (n= 60) | Telehealth program consisting of a workshop, telehealth practice immersion experiences, and a written project.  Topics for written projects: Role of technology in healthcare with a specific focus on rural settings, ways telehealth can be utilized for patient and caregiver care, education, and support (p.3).  Framework: Bandura’s social cognitive theory (Bandura, 1977). | Knowledge and skills. | Surveys and assignments. | Students rated the experience as very useful and realistic (4.9/5.0). Evaluation helped inform curriculum integration. |
| 1. Parmeter et al., 2023—USA*   Improving Telenursing  Skills Through  Simulation-Based Education. | A QI project—posttest only design  Second year DNP students (n=24) | A telehealth simulation via Zoom technology. Completion of online module prior to simulation, pre-briefing, simulation, and debriefing.  Topics: Tele-health knowledge (foundational knowledge regarding terminology, legal issues, telehealth etiquette, & best practices) (p. 96).  Framework: Ottawa Model for Research, INACSL. | Confidence, competent teaching skills | Telehealth performance rubric (Foronda et al, 2021), SET-M Tool (Leighton et al, 2015)—confidence domain. | Students demonstrated confidence with an average score of 2.875 out of 3, teaching performance scores were 2.7 out of 3, effective overall telehealth performance with a mean score of 13.74 out of 15. |
| 1. Burt & Kilroy, 2021—USA   Nurse Practitioner Student Perceptions of a Multimodal  Telemedicine Clinical Course. | Overview of curricular changes in an NP clinical course to incorporate tele-medicine competencies.  NP students (n=6) | Didactic learning module with faculty narrated PowerPoint presentations via self-directed learning, an 8-hour clinical practicum (synchronous and asynchronous), and three virtual simulation experiences using virtual SPs.  Topics: Overview of Telemedicine competencies (p. E1 23)  Framework: Kolb Cycle of Experiential Learning, PEARLS. | Satisfaction and confidence with simulation-focused learning  Perceptions of the multi-modal telemedicine-focused course. | End of semester survey and written reflection. | Student evaluations supported positive perceptions of multimodal integration of telemedicine competencies into an NP practicum course. |
| **DH** |  |  |  |  |  |
| 1. Beasley et al., 2023—USA*   Introduction to Digital Health Course. | Pilot of a newly developed course & process description.  APRN students within the DNP program (n=13) | Developed a graduate -level digital health course that facilitates implementation of DH services in current or future practice (a summer elective to allow for refinement before rolling it out). Five units with topics identified for each. The course includes multiple activities (self-evaluation assignment, discussion assignments, scholarly paper assignment and presentation, various experiences including a simulation day.  Topics: Introduction to DH; Laws, policies and procedures related to DH; Practical application of DH technologies; Benefits and limitations of DH utilization (p.65).  Framework: AACN Essentials, NONPF. | Perceived telehealth competence. | Post course evaluation (rating & narrative comments), Pre-post survey of telehealth competencies. | The overall average score on the telehealth competency survey was 2.38/4.00 pre-test and 3.16/4.00 post-test. All 22 telehealth competencies were improved from pre- to post-test. |
| 1. Seckman & van de Castle, 2021—USA   Understanding Digital Health Technologies Using Mind Maps. | Descriptive course evaluation project.  DNP students (n=163) | Mind maps tools (MindManager®) as a teaching strategy within 1-credit hour practicum experience along with web-conferencing sessions to explore DH technologies. Prior informatics courses exist. Learning contracts.  Topics: Scope, complexity, and integration of DH technologies, complex digital systems to support patient safety, efficiency and quality, e-patient engagement technologies, associated data sources, data element critical for patient care (p. 9).  Framework: AACN (2006) | Knowledge and application | Course evaluation questionnaire, rubrics, reflections in small groups. | The mind maps activity fostered critical thinking. Course evaluation scores (M=4.35) indicating mind maps as logical, relevant, appropriate, and meaningful to learning. |
| **EHRs** |  |  |  |  |  |
| 1. Choi et al., 2021a—USA   Nursing Informatics Competency after Experiencing Simulated Electronic Health Records: Descriptive Study. | Quantitative, Descriptive Design.  FNP students (n=23) | An assignment in a Health IT course. Simulated EHR (DocuCare). A video to train students on how to use the EHR. Two weeks to complete the assignment.  Topics: Learning core concepts of the DIKW framework. Electronic documentation (p. 145).  Framework: Not specified. | Perceived NI competency. | SICS survey.  An existing survey questionnaire. | NI competency level was between competent and proficient; basic computer skills and roles were above proficient; and advanced computer skills (clinical informatics) fell between competent and proficient levels. |
| 1. Choi, et al., 2021b—USA   Impact of Simulated Electronic Health Records on Informatics Competency of Students in Informatics Course. | Quantitative—Two-group independent measure design.  39 FNP students (intervention (n=19), Control=20) | Simulated EHR (DocuCare) as an assignment in an online informatics course. Customized 10-built-in scenarios from DocuCare. Intervention and control groups used same course materials. All students completed 5-interactive discussion forums, exams, & two paper assignments. One of the two paper assignments was replaced with the DocuCare (simulated EHR) assignment in the intervention group.  Framework: Not specified. | Perception of the simulated EHR, Perceived NI competency. | Two Surveys: 1) CICS—both groups, & 2)  Perception of simulated EHR questionnaire—Intervention group. | Students in the DocuCare group scored higher on the mean informatics competency than those in the control group. Students indicated use of the simulated EHR was enjoyable and useful to learning NI concepts. |
| **NI** |  |  |  |  |  |
| 1. Galacio, 2022—USA   Expanding the Informatics Competencies of Nurse Practitioners Through Online Learning. | One-group quasi-experimental design.  15 Practicing NP and 2 NP students | Online learning module (6 informatics-related topics), short videos followed by learning quizzes.  Topics: NI overview, Datasets and Microsoft Excel©, EHR data reporting, Telehealth best practices, Evaluating online information, Malware, and phishing overview (p. 609).  Framework: Adult Learning Principles | Perceived NI competency. | Pre-Post survey (SANIC). | Statistically significant median increases were identified in five areas. The online module was helpful in expanding students’ understanding of NI. |
| 1. Lokmic-Tomkins et al. 2021—Australia   Advancing Nursing Informatics Through Clinical Placements: Pilot Study. | Pilot study (prospective qualitative study using phenomenological approach)  MN students (n=4) | 4-week clinical placement focused on using the EMR. Briefing on clinical placement expectations was provided.  Topics: NI as a potential career pathway. Learning to use the EMR, using the EMR to make clinical decisions, nursing workflows, documenting nursing care (p.99).  Framework: ANSAT adapted to reflect EMR competencies. | Perceptions about NI in clinical practice. | Students completed reflective diaries at least twice a week (40hr working week). Also used the ANSAT to assess competencies. | Themes: Importance of adequate training in using EMR, Impact of EMR on nursing workflow and patient care, Shaping future career choices, Forming rewarding relationships, Potential for improvement of the experience. |
| 1. Beckham & Riedford, 2014—USA   Evolution of a Graduate-Level Informatics Course for the Non-informatics Specialist  Nurse. | Quantitative descriptive design.  NP students (n=175) | Asynchronous 3-hour credit online NI elective course. Pilot findings revealed improvement for computer application and knowledge based on pre-posttest, but the SNCEQ did not correlate to the open-ended questions. These findings were used to redesign the course.  Topics: Initial objectives based on AACN’s Essentials: “1) analyze current and emerging technologies; 2) evaluate outcome data; 3) incorporate ethical principles for technology use; 4) develop strategies to document patient care and measure outcomes; and 5) use technology to educate patients, guide practice, and support lifelong learning” (p. 388).  Framework: AACN's, The Essentials of Master's Education in Nursing, Bloom’s Taxonomy. | Development of information management confidence and skills. Linking NI concepts to the practice environment. | The SNCEQ was offered before and after the NI course. | The revised course focused on linking theory and nursing practice in course objectives, lectures, and assignments. Teaching strategies included recorded lectures, textbook and additional readings from scholar journals, 5 activities and 2 scholarly papers. Focus on higher-order thinking. |
| 1. McBride et al., 2013—USA   Developing an Applied Informatics Course for a Doctor of Nursing Practice Program. | Process for developing and evaluating an NI course.  DNP Students | An online NI course developed and taught over 4 yrs. [2008-2012], with 3 face-to-face intensives per semester using a seminar approach Multiple teaching strategies including hands-on activities, guest speakers, students’ projects, and others.  Topics: Module 1: (the field of informatics, TIGER initiative, nursing’s role within healthcare informatics, historical trends leading to the technology revolution in healthcare, the impact of the nursing informaticist, and TIGER NI Competencies. Module 2: (Patient safety, quality, and population health initiatives in healthcare informatics.  Module 3: (Database management and analysis, common terminology, and clinical decision support. Module 4: Point-of-care technologies (p. 39-40).  Framework: NEHI framework, AACN Essentials, Knowlton’s conceptual framework for online education, TIGER competencies | Establishing an overarching framework for teaching informatics content into practice. | Formative & summative evaluation of content and delivery approach (focus groups and satisfaction survey) to identify gaps between students’ expectations and actual experience. | Both focus groups and gap analysis values indicated no identified gaps between students’ expectations and their actual experience in the course, high satisfaction with the course content and delivery. Using the frameworks in the course design increased success of the course. |

**Legend:** NP - Nurse Practitioner; FNP- Family Nurse Practitioner; APRN - Advanced Practice Registered Nursing; DNP - Doctor of Nursing Practice; OSCE - Objective structured Clinical Examination; PEARLS - Promoting Excellence and Reflective Learning through Simulation; SOAP - Subjective, Objective, Assessment, and Plan; NEHI - Nursing Education Healthcare Informatics; NONPF - National Organization of Nurse Practitioner Faculties; AACN – American Association of Colleges of Nursing; EHR – Electronic Health Record; NI – Nursing Informatics; DH - Digital Health; VSLV- Virtual Simulated Learning Environment; ANSAT - Australian Nursing Standardized Assessment Tools; INACSL *-* International Nursing Association of Clinical Simulation and Learning; Q/A – Question and Answer; SBE – Simulation Based Learning; N/A- Not Applicable; RAM- Roy’s Adaption Model; TAC-Technology Acceptance Model; PDSA - Plan-Do-Study-Act; SANIC - Self-Assessment of Nursing Informatics Competency Scale; SICS - Self-Assessment of Informatics Competency Scale for Health Professionals; EMR- Electronic Medical Record; SNCEQ – Staggers Nursing Computer Experience Questionnaire; TIGER - Technology Informatics Guiding Education Reform; Yr. – Year; LMS - Learning Management System; 4Ps: Planning, Preparing, Providing & Performance Improvement; SP- Standardized Patient; OUD - Opioid Use Disorder; NLN – National League for Nursing; RN- Registered Nurses; TAM - Technology Acceptance Model; IP – Intraprofessional; SOAP - Subjective, Objective, Assessment and Plan; SET-M - Simulated Effectiveness Tool-Modified; DIKW - Data, Information, Knowledge, & Wisdom; QI – Quality Improvement

(*): COVID Publication

**Table 3: Curricular status of integration and proposed strategies (n= 33)**

| **Author, Year, Country** | **Focus** | **Design/Pop.** | **Instructional Strategy/Approach** | **Outcomes measured or explored** | **Evaluation/Assessment strategies** | **Key Findings** |
| --- | --- | --- | --- | --- | --- | --- |
| **Determining Status of Integration** |  |  |  |  |  |  |
| 1. Hamilton, 2023—USA*   A Mixed-Method Pilot Project: The Evaluation of Telehealth Training in a College of Nursing. | Telehealth | Mixed-Method pilot study for course evaluation  NP students (n=139) | Asynchronous  web-based, module-based THC. The design framework included creating learning objectives based on the course goal, creating scaffolded modules with pretests/ posttests, interactive activities, videos, and linked resources, integrating THC into the DNP Health Information Technology class [2019 and 2020].  Topics: The topics for each module are: What is Telehealth? (telehealth and telemedicine terminology), Using Telehealth (peripherals, webcam placement, proper lighting, and meeting environments,  Patient engagement, telepresence strategies, and proper clothing), Patient Considerations (community awareness aspects for rural areas, proper etiquette, and social presence), and Legal/Ethical Considerations (state and federal laws). (P. 2)  Framework: Mezirow’s TLT. ADDIE Design Approach, | Efficacy of the course | Pre-posttest scores comparison  within each module and critical student reflections. | Statistically significant knowledge gain was noted for all modules. |
| 1. Eckhoff, Guido-Sanz et al., 2022—USA*   Telehealth across nursing education: Findings from a national study. | Telehealth | A national survey. To determine the current or future use of telehealth content and experiences  386 Pre-licensure and graduate nursing programs (n=82; 21%)  Graduate (n=55) & Undergraduate: (n=82) | Programs reported having classroom telehealth content (lecture, online modules, videos, hands-on-practice, simulation, or skill lab—variable % of integration. Telehealth simulation experiences included history-taking, communication (role-play, on-call, phone calls/triage), physical assessment, mental health assessment, review lab results, learning to use telehealth equipment). | Not Applicable | Not Applicable | Variable integration |
| 1. Chike-Harris 2021—USA   Telehealth Education of Nurse Practitioner Students. | Telehealth | Program evaluation.  NP (Post BSN-to DNP) curriculum. | Curriculum mapping and scaffolding of telehealth components within 10 of the 18 NP courses and assessing impact. Strategies used included didactic—self-paced online modules, narrated lectures, F2F lectures, guest speakers, case studies, and assignments. Experiential: hands-on equipment training tele-presenter-tele-provider mock visits, & telehealth simulations with standardized patients. Student projects, &  clinical rotations.  Topics: Content covered in narrated or live lectures: Telehealth definitions, modalities, equipment needs; telehealth etiquette and therapeutics; introduction to telehealth cart; State and federal laws and billing; NP telehealth roles (p. 311)  Framework: A multi-modal framework proposed by Rutledge (2017), NONPF. | Telehealth knowledge | Student feedback (Pretest and post-tests surveys, n=263) | Knowledge gain improved based on comparing pre-test-posttest results of the narrated lectures. Positive student feedback regarding experiential components. |
| 1. Ali et al 2015—USA   Telehealth Education in Nursing Curricula. | Telehealth | Cross-sectional Survey  Deans & Directors of nursing programs (n=43) Undergraduate and Graduate | Technological strategies: Online education, clinical simulation, telehealth, flipping the classroom.  Clinical/simulation experiences: Remote telehealth unit, telehealth tools, patient education, remote patient monitoring devices, distance education, store & forward telehealth transmission, real-time telehealth communication, grand rounds, administrative uses, research, & telephony (p. 268).  Topics: Telehealth content: Definitions of terms, evidence-based telehealth practice, telehealth policies, telehealth standards, ethical guidelines. (p. 267).  Framework: Not specified. | Perceptions of telehealth education, content, learning experiences in clinical and simulation, support to faculty, & barriers. | Not Applicable | Inadequate integration in classroom content, simulation, and clinical experiences. Interviewed 4 nurse leaders to identify recommendations for integration of telehealth. |
| 1. Park et al., 2022—South Korea   Development of a Standardized Curriculum for Nursing Informatics in Korea. | NI | Delphi Survey.    Out of 104 programs that offered NI courses, 60 agreed to participate (n=53, 88.3%). | NI curricular integration and curriculum development. The learning objectives identified were based on NI competency frameworks and professional practice standards. Two-round Delphi survey with 15 experts to determine demand for each objective.  Topics: Topics identified for graduate curriculum: Healthcare IT concepts, the use of IT in evidence-based practice, the use of IT in community nursing, and the use of genome information, AI, clinical information systems, administrative management systems, and IT in nursing education (p. 349).  Framework: The 2015 NI Practice Standards and Scope of the ANA, TIGER-based assessment on NI, QSEN and HIMSS, Healthcare Leaders Association, and CASN ICT competencies.  NI competencies were developed based on the ANA Competencies. | Developing a standardized NI curriculum. | Not Applicable | The standardized curriculum included 46 objectives for the undergraduate level and 26 objectives for graduate levels.  Key topics appropriate for each level were identified. |
| 1. Shobuzawa et al. 2021—Japan   Availability of Nursing Informatics  Education for Master’s Programs in Nursing Administration at Graduate Schools of Nursing in Japan. | NI | Internet search  Graduate schools of nursing providing MN programs in nursing administration. | Identify which programs teach NI based on published course syllabi. | Not Applicable | Not Applicable | Results showed half of the master’s programs in nursing administration at graduate schools of nursing do not have NI educational content. NI is offered as a separate subject or included in other nursing administration subjects. |
| 1. O'Connor & LaRue, 2021—UK   Integrating informatics into undergraduate nursing education: A case study using a spiral learning approach. | NI | Case study.  BSN curriculum. | A review of literature and a mapping exercise to outline core informatics competency domains and learning descriptors. Individual learning units based on 6 competency domains. A spiral learning framework for leveling competencies across existing courses. Developed and evaluated one unit focused on digital professionalism.  Topics: Individual learning units in health informatics based on six competency domains: (1) health service literacy, (2) information and communication technology literacy, (3) information management, (4) information systems literacy, (5) information systems management, and (6) patient/citizen digital health literacy (p.3).  Framework:  Spiral learning approach. Discussed different frameworks to be implemented for a wider evaluation of the new NI curricula integrated. | Students’ perspectives on the value of digital professionalism education. | Evaluation of the digital professionalism unit by 2^nd^ yr. students enrolled in an Adult Nursing Course (n= 18/325) using an online survey—very low response rate. Only qualitative results reported. | Students valued the learning experience. Themes identified: Boundaries that students were more aware of after receiving education on digital professionalism, the benefits of this type of training for their future nursing careers, & how to use technology to communicate appropriately. |
| 1. Bove, 2020—USA   Integration of Informatics Content in Baccalaureate and Graduate Nursing Education. | NI integration | Internet search. Updated Status Report.  Top 25 nursing schools offering  online nursing Programs (BSN, MS, DNP, & PhD). | Reviewed course titles for NI courses | Not Applicable | Not Applicable | 67% of the schools with master's programs and more than 80% of the DNP programs included at least one informatics course. Schools with online BSN courses had about the same number of programs with informatics courses as the 2013 report. |
| 1. Jeon et al., 2016—South Korea   Current Status of Nursing Informatics Education in Korea. | NI | Questionnaires  204 nursing schools (n=72; 35.3%).  Undergraduate and graduate nursing programs | Identified 14 core NI content domains with 41 subcategories based on reported NI competencies & reviewed NI courses and subjects.  Topics: Subjects taught based on analysis of 27 course syllabi: Computer technology, Information systems used in practice, Telehealth, Informatics, Nursing, Ethics, Information systems used for education, research, and administration, Information literacy, Basic computer competencies, Careers/roles in NI, Latest trends, Information management (p. 146).  Framework: Several NI competency frameworks and recommendations (TIGER, QSEN, and others) | Not Applicable | Not Applicable | Integration increased overall. In total, 38 schools reported having NI courses in their undergraduate nursing programs. |
| 1. Stephens-Lee et al., 2013—Canada   Preparing Students for an Electronic Workplace. | NI | A two-step iterative approach  BSN curriculum. | A review of program course blueprints and mapping against different NI frameworks to identify where NI concepts are integrated and where they could be further developed.  Topics: NI core concepts based on TIGER: Computer literacy, Information literacy, Information management. (p. NA).  Framework:  NI Frameworks: Hebert (1999), NI competency levels: entry-level, practicing nurse, and specialist; & Nagle (2001) NI competencies: basic NI concepts and operations, social and ethical human issues, productivity tools, communication tools, research tools and decision support systems; TIGER domains; DIKW. | Not Applicable | Email questionnaire and face-to-face meetings with faculty | The authors provided recommendations to further integrate informatics into the curriculum for each of the TIGER domains. |
| 1. Hunter et al., 2013—USA   The Integration of Informatics Content in Baccalaureate and Graduate Nursing Education: A Status Report. | NI | Internet search. Status report.  Top 24 nursing schools offering  online nursing Programs (BSN, MS, DNP, & PhD). | Reviewed course titles for NI courses.  Framework:  Not specified | Not Applicable | Not Applicable | Six schools had no informatics content in any level. The Web sites of the remaining 18 schools listed informatics content for 10 at the BSN level, 9 at the master’s level, and 4 at the doctoral level. One school had content in all 3 levels. Only 4 schools offered a focus on NI (p. 111). |
| 1. Nagle et al., 2020a—Canada   Digital health in Canadian Schools of Nursing Part A: Nurse Educators’ Perspectives. | NI & DH | Mixed methods:  Survey (n=360);  Focus Group (n=10) & one-on-one telephone interviews (n=10).    BSN curriculum | Variable % of integration and largely driven by individual faculty members—comprehensive integration examples were not provided.  Topics: CASN resources (NI competencies, Faculty Teaching Tool Kit, Consumer Health Resource Toolkit, Social media whiteboard animation video, Clinical data standards whiteboard animation video) (p. 4).  Framework:  NI entry-to-Practice Competencies for RNs in Canada | (1) current state of digital health content integration into nursing curricula, (2) nurse educators’ knowledge, experiences and needs in enhancing their development of digital health capacity now and in the future, (3) teaching and learning exemplars of digital health integration in nursing curricula, and (4) recommendations for advancing development of informatics and digital health in nursing education. | Not Applicable. | Only 4.7% teach a DH/NI course in the program, 44% teach aspects of same within another course, 17% invite a guest lecturer. A small number (n=20) identified as someone who helps colleagues by guest lecturing on DH/NI topics. Less than 20% use a simulated electronic health record. Limited use of CASN resources. Discussed strategies to build educators’ DH capacity. |
| 1. Nagle et al., 2020b—Canada   Digital health in Canadian Schools of Nursing Part B: Nurse Administrators’ Perspectives. | NI & DH | Mixed methods: Survey component  (Administrators, n=35)  BSN curriculum | Comparable findings as above. | Same as above | Not Applicable | Comparable findings as above. |
| 1. Raghunathan et al. 2022 –Australia   Utilisation of academic electronic medical records in pre-registration nurse education: A descriptive study. | EHR | Quantitative descriptive study (Survey)  Nursing programs (Australia and New Zealand, n=37) | Reported on the utilization of AEMR in curriculum to teach skills and in simulation laboratory settings to teach essential nursing practice skills and core clinical topics. | Not Applicable | Not Applicable | Not specified |
| **Proposed Strategies** |  |  |  |  |  |  |
| 1. Lattuca et al., 2023—Canada   Healthcare AI: A Revised Quebec Framework for Nursing  Education | AI competencies | Literature review, document analysis, & consultations with experts. Nursing curricula (BSN, MSN, & PhD levels) and practice (RN). | Development of AI competency framework.  Framework:  CASN’s domains of Nursing education (2014, 2022). | Not Applicable | Not Applicable | AI competencies:  1) Understand the foundation of informatics and digital health technology prior to working with AIHT, 2) Define AIHT capabilities and their associated risks and limitations, 3) Interpret and effectively communicate AIHT results in terms of patient care, 4) Explain the ethical, social, and legal implications of AIHT, & 5) Apply critical thinking to analyze AIHT data (p. 5-6). |
| 1. Subramanian & Kleib, 2023—Canada.   Leveraging Clinical Preceptorship to Enhance Nursing Students’ Readiness in Digital Health. | DH | Discussion paper.  Senior-level BSN Students | Overview of strategies to strengthen the role of clinical preceptors in supporting students’ learning about DH (p. 4-6). | Not Applicable | Not Applicable | Not applicable |
| 1. Honey et al., 2020—New Zealand   Education into Policy: Embedding Health Informatics to Prepare Future Nurses—New Zealand Case Study | HI & NI | Case study.  BSN curriculum. | Developed 5 HI guidelines for entry-to-practice based on a literature review and refinement using iterative process and advisory group.  Framework:  ANIS for Nurses and Midwives, TIGER, Royal College of Nursing in England (Every nurse an e-nurse: Digital capabilities for 21st century nursing), Canadian NI Entry-to-Practice Competencies for RNs. | Key knowledge, skills, and behaviors toward NI for nurses to inform undergraduate nursing education | Not Applicable | Not Applicable |
| 1. Bobek, 2022—USA   Teaching Strategies for Online Nurse Practitioner  Physical Assessment and Telehealth Education. | Telehealth | Discussion paper.  Graduate nursing students | Different strategies on telehealth education (didactic content, simulation, and clinical experiences guided by telehealth competencies) and an overview of resources for educators and providers regarding telehealth education.  Framework:  NONFA, 4P of telehealth competencies. | Not Applicable | Not  Applicable | Not  Applicable |
| 1. Dzioba et al., 2022—USA   Telehealth Competencies  Leveled for Continuous Advanced Practice Nurse Development. | Telehealth | An iterative, 3- process (applying the framework, leveling telehealth competencies, & refinement of competencies).  APNs curricula | Using telehealth competencies to inform curricular integration and continuous education.  Telehealth sub-competencies were leveled for preclinical and clinical rotations and for readiness for practice.  Examples of learning activities for evaluating competence within the Four Ps framework are provided.  Framework: Benner’s novice to expert theory, Four Ps of Telehealth Framework, Competency-based education. | Not Applicable | Not Applicable | Not Applicable |
| 1. Taylor & Fuller, 2021—USA   The expanding role of telehealth in nursing:  considerations for nursing education. | Telehealth | A discussion paper based on a literature review. | How telehealth can be integrated in nursing education (theory & clinical)? Proposed strategies: didactic lectures, webinars/seminars, projects, discussion boards, simulation, and examples of how these can be designed and delivered.  Framework:  Proposed: Constructivism - Knowles’ theory of andrology, Knowledge, skills, and attitudes based on telehealth competencies. | Not Applicable | Not Applicable | Not Applicable |
| 1. Fronczek et al., 2017—USA   Enhancing Telehealth Education in  Nursing: Applying King’s Conceptual  Framework and Theory of Goal  Attainment. | Telehealth | Discussion Paper.  Undergraduate and graduate nursing curricula. | A discussion of how and why telehealth integration is important, providing application of relevant theories and standards.  Framework:  King’s conceptual framework and theory of goal attainment.  AACN (2008) Essentials. | Not Applicable | Not Applicable | Not Applicable |
| 1. Guenther et al. 2021—USA*   Five Steps to Integrating Telehealth Into APRN Curricula. | Telehealth | Discussion paper.  APRN curriculum  Faculty | Provided components and steps in integrating the telehealth in the curriculum. | Not Applicable | Not Applicable | Not Applicable |
| 1. Vottero, 2017—USA   Teaching Informatics to Prelicensure, RN-to-BSN, and Graduate Level Students. | NI | Discussion paper.  Prelicensure, RN-to-BSN, & graduate level curricula | Best pedagogical practices for aligning the competency outcome with teaching and assessment strategies.  NI key course content & leveling approaches.  Recommended different teaching strategies such as sharing experiences, discussions, self-reflection, awareness of knowledge, skills, attitudes, simulation, etc. Examples of teaching strategies and leveling are provided.  Framework: QSEN, AACN Essentials, NI competencies | Not Applicable | Not Applicable | Not Applicable |
| 1. Weiner et al., 2016—USA   Integrating Informatics Content into the Nursing Curriculum. | NI | Discussion paper.  BSN, MSN, DNP, and PhD curricula. | Proposed a model for leveling/integrating NI with varying NI proficiencies according to curricular level of instruction & NI roles.  Framework: TIGER competencies | Not Applicable | Not Applicable | Not Applicable |
| 1. Jenkins, 2018—USA   Informatics Essentials for DNPs. | NI | Discussion paper.  DNP curricula. | A required online course was revised and updated to provide clinical informatics education. Course objectives and learning outcomes provided. Strategies included content (readings and multimedia), independent study assignments, a combination of questioning and collaborative learning in discussion boards, and evaluation (p.55).  Topics: Information technology and data management, Critical appraisal of standards for documentation, Modeling, and transmission of high-quality, reliable data for use in clinical care, Healthcare administration, & Quality improvement.  Framework: AACN (2006),  Picciano’s Multi-modal Model for Online Education | Not Applicable | Not Applicable | Not Applicable |
| 1. Wolf & Morouse, 2015—USA   Using Blogs to Support Informatics Nurses' Curriculum Needs. | NI | Process of developing an NI course assignment  MSN curricula (informatics track) | Using blogs in an online course. 7 learning outcomes and 14 weeks of curricular content relative to consumer health informatics.  Framework:  American Telemedicine Association white paper on telehealth, 2015 ANA Scope and Standards of Practice for Nursing Informatics. | Development of skills for using technology to promote health. | 4 required assignments challenging students to develop creative strategies in applying technologies. | Not Applicable |
| 1. Frisch & Borycki, 2013—Canada   A Framework for Leveling Informatics  Content Across Four Years of a Bachelor  of Science in Nursing (BSN) Curriculum. | NI | Discussion Paper  BSN curriculum | Proposed a framework for NI content integration and leveling (simple to complex) in existing courses across the four years with examples.  Framework: Content areas of the curriculum for organizing NI content; CASN and TIGER NI competency frameworks. | Not Applicable | The UVic Informatics Practice Appraisal Tool to track learning and outcomes. | Not Applicable |
| 1. Spencer, 2012—USA   Integrating Informatics in Undergraduate Nursing Curricula:  Using the QSEN Framework as a Guide. | NI | Discussion paper.  BSN curriculum. | Overview of teaching strategies and learning activities (e.g., Assignments, web site evaluation, and self-assessment) implemented in the classroom, simulation lab, and clinical.  Framework: QSEN. | Knowledge, skills, and attitudes in NI |  | Post three years of integration, 90%-97% of the comments made by students in the first-term course evaluation of learning activities were positive. |
| 1. Swenty & Titzer, 2014—USA   A Sense of Urgency: Integrating  Technology and Informatics in Advance Practice Nursing Education. | Technology & informatics | A hypothetical education program.  APN curricula | Provided a model on how faculty can integrate informatics in existing courses, outlining objectives, activities, and student outcomes for each course.  Framework: Kotter’s sense of urgency model, NI identified competencies. | Not applicable | Not Applicable | Not Applicable |
| 1. Titzer & Swenty, 2014—USA   Integrating an Academic Electronic Health Record in a Nursing Program: Creating a Sense of Urgency and  Sustaining Change. | Technology & informatics | Discussion paper.  BSN curriculum | Provided a strategic plan to integrate an AEHR within an existing BSN curriculum.  Framework: Kotter’s sense of urgency model, QSEN, TIGER, AACN essentials. | Integration of informatics in the curriculum. | Student focus groups, reflection, observation.  Faculty survey 2 years after implementation | Initial response by students is that the AEMR was an added work, but it improved later. Faculty (n=14) use varied according to courses, but generally adopted and championed change. |
| 1. Eardley et al. 2021—USA   Quality Improvement Project to Enhance Student Confidence Using an Electronic Health Record. | EHRs | A quality improvement project. Pilot study  BSN & graduate programs (n=11) | A framework to adopt, implement, and evaluate an AEHR in two prelicensure programs. A blueprint provided for using an AEHR in nursing education curriculum.  Framework: SLCM | Knowledge level, perceived confidence, and competence | Not applicable. | Significant improvement was noted in knowledge, competence, and confidence in using an EHR in a clinical setting. |
| 1. Sorensen & Campbell, 2016—USA   Curricular Path to Value: Integrating an Academic Electronic Health Record. | EHRs | Discussion paper.  BSN program.  Faculty and students | Provided teaching strategies that promoted the ease of integrating an AEHR across a cur­riculum. Highlighted foundational and advanced levels of threading throughout the curriculum.  Framework: Not specified. | Integration of AEHR in the courses increased form two courses into eight courses. |  | Student feedback and course evaluations were positive.  Faculty feedback stressed an appreciation for having an innovative way to educate nursing students. |
| 1. Kunkel et al. 2024—USA   Creating Case Studies for Digital Health and Technology Competency in Nursing. | Informatics, DH & Technology | Case study.  Nursing program. | Proposed a process to create case studies for including informatics, DH, and clinical reasoning competencies across the curriculum.  Topics: Quality improvement, nanotechnology in diabetes management, and unintended consequences of EHRs.  Framework: Not specified. |  | Not applicable. | Not Applicable |

**Legend.:** BSN – Bachelor of Science in Nursing; MS – Master of Science; MN/MSN – Masters in Nursing Science; NP- Nurse Practitioner; APRN – Advanced Practice Registered Nurse; ANP – Advanced Practice Nurse; Ph.D.- Doctoral; DNP – Doctor of Nursing Practice; D.H - Digital Health; NI- Nursing Informatics; HI – Health Informatics; TIGER -Technology Informatics Guiding Education Reform; QSEN - Quality and Safety Education for Nurse; HIMSS - Healthcare Information Management System Society; CASN - Canadian Association of Schools of Nursing; AI - Artificial Intelligence; AACN – American Association of Colleges of Nursing; ANA – American Nurses Association; NONPF – Nursing Organization of Nurse Practitioner Faculties; ICT – Information and Communication Technology; TLT – Transformative Learning Theory; DIKW—Data, Information, Knowledge, & Wisdom; RN – Registered Nurses; UVic – University of Victoria; SLCM - Systems Life Cycle Model; AEHR - Academic Electronic Health Record; ADDIE – Analysis, Design, Development, Implementation, and Evaluation; APN- Advanced Practicing Nursing; THC – Telehealth Course; AEMR – Academic Electronic Medical Record; 4Ps: Planning, Preparing, Providing & Performance Improvement; AIHT – Artificial Intelligence Health Technologies; ANIS - Australian National Informatics Standards; SP - Standardized Patients;

(*): COVID Publication
