# Supplementary material for: Digital Health Education and Training for Undergraduate and Graduate Nursing Students: Scoping Review
Source: JMIR Nurs. 2024 Jul 17;7:e58170. doi: 10.2196/58170 (PMC11292154; doi:10.2196/58170)
Supplement: Multimedia Appendix 4 [file nursing_v7i1e58170_app4.docx]

**Appendix III: Organizations providing digital health education**

| **Provider** | **Digital Health Education Offerings** |
| --- | --- |
| **Digital Health Canada** | [Virtual Care Certificate](https://digitalhealthcanada.com/learning-pathways/individual-learning/)   - Audience: Healthcare professionals, health IT professionals, and people interested in virtual care. - Online, Asynchronous   Objectives:   - Provide the definition and scope of virtual care and provide the advantages and disadvantages of different care delivery methods. - Identify the various virtual care applications across jurisdictions and provide considerations when planning or scaling virtual care delivery.   [Core Health Informatics Course](https://digitalhealthcanada.com/learning-pathways/individual-learning/)   - Audience: Individuals interested in digital health, health informatics, healthcare delivery, and Canadian health. - Online modules, asynchronous. - Each module has specific learning outcomes. |
| **HIMSS** | [Digital Health Transformation Course](https://www.himss.org/events/digital-health-transformation-course)   - Professional development course,10 learning modules, self-paced. - Online, asynchronous, approximately 10 hours for completion.   Objectives:   - Illustrate the ways digital infrastructure improves productivity, access, efficiency, and performance of health systems and explain ways healthcare systems can assess the value of new approaches and treatment modalities to unique populations. - Identify components required for digital connectivity to the formal healthcare system. - Demonstrate the importance of digital infrastructure as a key requirement for global health systems.   [HIMSS Data Analytics, Interpretation and Reporting Course](https://www.himss.org/events/himss-data-analytics-interpretation-and-reporting)   - Audience: Individuals interested in healthcare data analytics - Online, asynchronous, approximately 10 hours to complete course time.   Objectives:   - Identify and interpret data to inform business decisions. - Perform data preparation procedures. - Recognize trends, detect outliers, and summarize data sets. - Analyze relationships between variables. - Design data visualization using the most appropriate graphical methods and tools.   [Healthcare Information and Technology Foundation Course](https://www.himss.org/events/healthcare-it-foundations-course)   - Online, asynchronous. - Content covered includes Healthcare Environment, Technology Environment, Analysis and Design, Selection and Acquisition, Implementation and Management, Privacy and Security, Leadership and Planning, Professionalism and Communication Skills, and Clinical Informatics. |
| **Coursera** | [Digital Health Specialization](https://www.coursera.org/specializations/digitalhealth)   - Online modules, asynchronous - Each module has specific learning outcomes. - General course overview: The specialization introduces students to the emerging and multidisciplinary field of digital health and the role and application of digital health technologies, including mobile applications, wearable technologies, health information systems, telehealth, telemedicine, machine learning, artificial intelligence, and big data.   [Leading Change in Health Informatics](https://www.coursera.org/learn/leading-change-health-informatics)   - Online, asynchronous, offered in 4 modules, approximately 15 hours to complete.   Objectives:   - Describe essential steps in the process of successfully leading change in health IT organizations. - Utilize several common project management and strategic planning tools used by health informaticists. - Recognize the importance of multidisciplinary and interprofessional teamwork in health informatics. - Apply best practices for successful change management in health informatics.   [Nursing Informatics](https://www.coursera.org/learn/nursing-informatics)   - Online, asynchronous, offered in 6 modules, approximately 7 hours to complete. - Assessment/learning strategies quizzes and videos   [Using Clinical Health Data for Better Healthcare](https://www.coursera.org/learn/healthcare-data)   - Online, asynchronous, offered in modules, approximately 15 hours to complete (3 weeks at 5 hours a week). - Assessment/learning strategies include quizzes, readings, and discussion prompts.   Overall objectives:   - Identify digital health technologies, health data sources, and the evolving roles of the health workforce in digital health environments. - Understand key health data concepts and terminology, including the significance of data integrity and stakeholder roles in the data life cycle. - Use health data and basic data analysis to inform and improve decision making and practice. - Apply effective methods of communication of health data to facilitate safe and quality care.   [eHealth: More Than Just an Electronic Record](https://www.coursera.org/learn/ehealth)   - Audience: health clinicians, students, managers, administrators, and researchers. - Online, asynchronous, approximately 12 hours to complete all the 5 modules (3 weeks at 4 hours per week). - Each module has learning outcomes. - Assessment/learning strategies includes quizzes, peer review, discussion prompts, readings, and videos.   Overall Objectives:   - The fundamentals of eHealth and where it is heading. - What kind of health data we are currently collecting and how it will transform healthcare in the future. - How new technologies are helping health consumers participate in their own healthcare. - How eHealth can improve the coordination and efficiency of healthcare and what the barriers might be.   [Health Informatics for Healthcare Professionals](https://www.coursera.org/learn/health-informatics-for-healthcare-professionals)   - Audience: Individuals working in the healthcare sector, as a provider, payer, or administrator. - Online, asynchronous, approximately 13 hours to complete 4 modules in 3 weeks at 4 hours a week. - Each module has learning outcomes. - Assessment/learning strategies include quizzes, videos, discussion prompts, and readings.   Overall Objectives:   - Describe the history and current status of information systems in healthcare. - Identify and describe the functions and evolution of the electronic health record systems. - Interpret the relationship between information retrieval, data standards and decision support system.   [AI in Healthcare Specialization](https://www.coursera.org/specializations/ai-healthcare?utm_medium=sem&utm_source=gg&utm_campaign=B2C_EMEA_ai-healthcare_stanford_FTCOF_specializations_country-GB-country-UK&campaignid=20790965509&adgroupid=154406897503&device=c&keyword=&matchtype=&network=g&devicemodel=&adposition=&creativeid=681326628475&hide_mobile_promo&gclid=Cj0KCQiA4Y-sBhC6ARIsAGXF1g6AUzkXhQz2IuA7hrr_TjDwyhzPced0VmcrCnlX4J5dsdnPGR2jy5IaAiyeEALw_wcB)  Online, asynchronous, approximately 1 month at 10 hours a week to complete 4 modules.   - Audience: healthcare providers and computer science professionals. Each module has specific objectives.   Overall Objectives:   - Discuss the current and future applications of AI in healthcare with the goal of learning to bring AI technologies into the clinic safely and ethically. - Identify problems healthcare providers face that machine learning can solve. - Analyze how AI affects patient care safety, quality, and research. - Relate AI to the science, practice, and business of medicine. - Apply the building blocks of AI to help you innovate and understand emerging technologies |
| **CNIA** | [Digital Health Education Resources](https://cnia.ca/)   - Online, asynchronous. - Audience: Nursing students and nurses - Resources are available that support digital health education in the form of webinars, videos, and informational series. |
| **CASN** | [Digital Health Modules](https://digitalhealth.casn.ca/)   - Online, asynchronous, self-paced, offered in 4 modules. - Audience: Nursing students and nurses. - Each learning module has specific learning outcomes that address the overall objectives of the modules. - Extensive resources that support the digital health education of graduate and undergraduate nursing students. - Resources available include PowerPoint presentations, videos, webinars, and interest groups. |
| **OpenWHO** | [Ethics and Governance of Artificial Intelligence for Health](https://openwho.org/courses/ethics-ai)   - Online, asynchronous, self-paced, approximately 3.5 hours to complete 7 modules. - Audience: Policymakers, AI developers and designers, and healthcare providers - Assessment: Quiz, post-course survey   Overall objectives:   - Describe the opportunities and benefits of using AI in healthcare. - Outline the ethical considerations and explain the ethical challenges of its use. - Describe liability considerations associated with the design, development, and use of AI for health. - Summarize frameworks and regulatory considerations for the design, development, and use of such technology in the sector.   [Infodemic Management](https://openwho.org/courses/im-in-the-field)   - Online, asynchronous, self-paced - There are 7 series under this course; each series has modules with specific learning objectives, and the completed approximated time ranges from 1.5hrs to 4 hours. - Assessment includes pre-course assessment and quizzes. |
